# Push-Pull Chromophores Based on the Naphthalene Scaffold: Potential Candidates for Optoelectronic Applications

**DOI:** 10.3390/ma12081342

**Published:** 2019-04-24

**Authors:** Corentin Pigot, Guillaume Noirbent, Thanh-Tuân Bui, Sébastien Péralta, Didier Gigmes, Malek Nechab, Frédéric Dumur

**Affiliations:** 1 Aix Marseille Univ, CNRS, ICR UMR7273, F-13397 Marseille, France; didier.gigmes@univ-amu.fr (D.G.); malek.nechab@univ-amu.fr (M.N.); 2 Laboratoire de Physicochimie des Polymères et des Interfaces (LPPI), Université de Cergy Pontoise, 5 mail Gay Lussac, F-95000 Neuville-sur-Oise, France; tbui@u-cergy.fr (T.-T.B.); sebastien.Peralta@u-cergy.fr (S.P.)

**Keywords:** push-pull dyes, chromophore, naphthalene, solvatochromism

## Abstract

A series of ten push-pull chromophores comprising 1*H*-cyclopenta[*b*]naphthalene-1,3(2*H*)-dione as the electron-withdrawing group have been designed, synthesized, and characterized by UV-visible absorption and fluorescence spectroscopy, cyclic voltammetry and theoretical calculations. The solvatochromic behavior of the different dyes has been examined in 23 solvents and a positive solvatochromism has been found for all dyes using the Kamlet-Taft solvatochromic relationship, demonstrating the polar form to be stabilized in polar solvents. To establish the interest of this polyaromatic electron acceptor only synthesizable in a multistep procedure, a comparison with the analog series based on the benchmark indane-1,3-dione (1*H*-indene-1,3(2*H*)-dione) has been done. A significant red-shift of the intramolecular charge transfer band has been found for all dyes, at a comparable electron-donating group. Parallel to the examination of the photophysical properties of the different chromophores, a major improvement of the synthetic procedure giving access to 1*H*-cyclopenta[*b*]naphthalene-1,3(2*H*)-dione has been achieved.

## 1. Introduction

**Figure 1:**
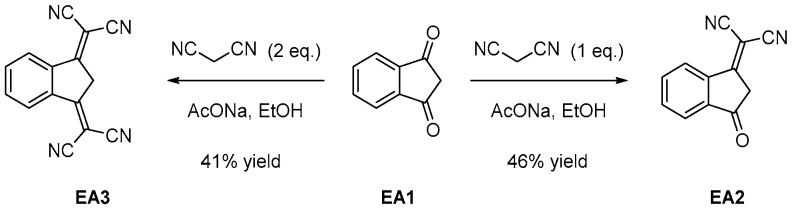
Electron acceptors **EA1–EA3**.

During the past decades, push-pull molecules have attracted much attention due to their numerous applications ranging from non-linear optical (NLO) applications [1,2] to organic photovoltaics (OPVs) [3,4], organic field effect transistors (OFETs) [5], organic light-emitting diodes (OLEDs) [6], photorefractive applications [7], colorimetric pH sensors [8], ions detection [9], biosensors [10], gas sensors [11], or photoinitiators of polymerization [12,13,14,15,16]. Typically, push-pull chromophores are based on an electron donor and an electron acceptor connected to each other by mean of a saturated or a conjugated spacer [17]. As the first manifestation of the mutual interaction between the two partners, a broad absorption band corresponding to the intramolecular charge transfer (ICT) interaction can be detected in the visible to the near-infrared region. Position of this absorption band typically depends on the electron-donating ability of the donor and the electron-releasing ability of the acceptor, and a bathochromic shift of this transition is observed upon improvement of the strength of the donor and/or the acceptor. This ICT band can even be detected in the near and far infrared region for electron-acceptors based on poly(nitrofluorenes) [18]. Among electron acceptors, indane-1,3-dione (1*H*-indene-1,3(2*H*)-dione) **EA1** has been extensively studied due to its commercial availability, its low cost and the possibility to design push-pull dyes by a mean of one of the simplest reaction of Organic Chemistry, namely the Knoevenagel reaction [19,20,21,22,23]. By chemical engineering, its electron-withdrawing ability can be drastically improved by condensation of one or two malononitrile units under basic conditions, furnishing 2-(3-oxo-2,3-dihydro-1*H*-inden-1-ylidene)malononitrile **EA2** [24] and 2,2’-(1*H*-indene-1,3 (2*H*)-diylidene)dimalononitrile **EA3 [25]** (see Figure 1). Based on the strong electron-withdrawing abilities of **EA2** and **EA3**, push-pull dyes with non-fullerene acceptors and small energy gaps have been developed [26,27]. As a possible alternative to improve the electron-accepting ability, extension of the aromaticity of the acceptor can be envisioned. Using this strategy, a red-shift of the ICT band combined with an enhancement of the molar extinction coefficient can be both obtained for these new push-pull derivatives, relative to that obtained with the parent electron acceptor.

**Figure 2:**
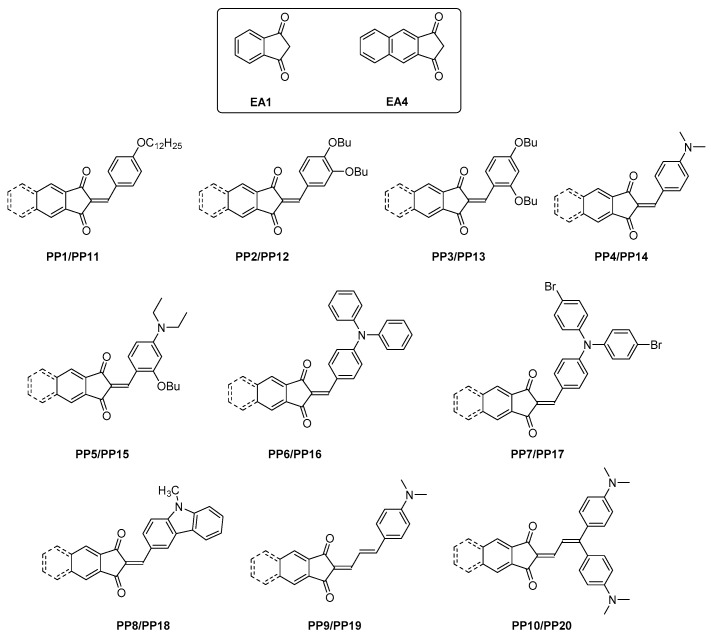
Chemical structures of the 20 dyes **PP1–PP20** examined in this study. **PP1–PP10** were made from **EA4**, while **PP11–PP20** were made from **EA1**.

In this article, an extended version of the well-known indane-1,3-dione, i.e., 1*H*-cyclopenta[*b*]naphthalene-1,3(2*H*)-dione **EA4**, has been used for the design of ten push-pull dyes. It has to be noticed that even if the synthesis of **EA4** is reported in the literature since 2006 [28], only one report mentions the development of anion sensors with **EA4** in 2013 [29] and the first push-pull dyes have been developed in 2017 for photovoltaics applications [30,31]. Considering the scarcity of studies devoted to this electron acceptor, a series of 10 push-pull chromophores **PP1**–**PP10** have been developed with **EA4**. To evidence the contribution of the additional aromatic ring in **EA4** relative to that of **EA1**, 10 dyes **PP11**–**PP20** comprising **EA1** as the electron acceptor have been synthesized for comparison (see Figure 2).

The photophysical properties of the series of 20 dyes as well as their electrochemical properties have been investigated. To support the experimental results, theoretical calculations have been carried out. Finally, the solvatochromic properties have also been examined.

## 2. Materials and Methods

All reagents and solvents were purchased from Aldrich, Alfa Aesar or TCI Europe and used as received without further purification. Mass spectroscopy was performed by the Spectropole of Aix-Marseille University. Electron spray ionization (ESI) mass spectral analyses were recorded with a 3200 QTRAP (Applied Biosystems SCIEX) mass spectrometer. The HRMS mass spectral analysis was performed with a QStar Elite (Applied Biosystems SCIEX) mass spectrometer. Elemental analyses were recorded with a Thermo Finnigan EA 1112 elemental analysis apparatus driven by the Eager 300 software. ^1^H and ^13^C nuclear magnetic resonance (NMR) spectra were determined at room temperature in 5 mm outer diameter (o.d.) tubes on a Bruker Avance 400 spectrometer of the Spectropole: ^1^H (400 MHz) and ^13^C (100 MHz). The ^1^H chemical shifts were referenced to the solvent peak CDCl_3_ (7.26 ppm) and the ^13^C chemical shifts were referenced to the solvent peak CDCl_3_ (77 ppm). UV-visible absorption spectra were recorded on a Varian Cary 50 Scan UV Visible Spectrophotometer, with concentration of 5 × 10^−3^ M, corresponding to diluted solutions. Fluorescence spectra were recorded using a Jasco FP 6200 spectrometer. The electrochemical properties of the investigated compounds were measured in acetonitrile by cyclic voltammetry, scan rate 100 mV·s^−1^, with tetrabutylammonium tetrafluoroborate (0.1 M) as a supporting electrolyte in a standard one-compartment, three-electrode electrochemical cell under an argon stream using a VSP BioLogic potentiostat. The working, pseudo-reference and counter electrodes were platinum disk (Ø = 1 mm), Ag wire, and Au wire gauze, respectively. Ferrocene was used as an internal standard, and the potentials are referred to the reversible formal potential of this compound. Computational details: All quantum mechanical calculations were computed using Gaussian Package [32]. All geometry optimizations were performed using the density functional theory (DFT) with the global hybrid exchange-correlation functional B3LYP [33] and all minima on the potential energy surface were verified via a calculation of vibrational frequencies, ensuring no imaginary frequencies were present. The Pople double-zeta basis set with a double set of polarization functions on non-hydrogen atoms (6-3111G(d,p))[34,35] was used throughout. This computational approach was chosen in consistency with previous works, as it provides good agreement with experimental data. Excited states were probed using the time dependent density functional theory (TD-DFT) using the same function. All transitions (singlet-singlet) were calculated vertically with respect to the singlet ground state geometry. Solvent effects were taken into account by using the implicit polarizable continuum model (PCM) [36,37]. Dichloromethane (DCM) where chosen in analogy with the experiments. Computed spectra were simulated by convoluting each transition with Gaussians functions-centered on each absorption maximum using a constant full width at half maximum (FWHM) value of 0.2 eV. The assignment of electronic transitions for λ_max_ has been determined with GaussSum 3.0 software [38]. 4-(Dodecyloxy)benzaldehyde **D1 [39]**, 3,4-dibutoxy-benzaldehyde **D2 [40]**, 2,4-dibutoxy-benzaldehyde **D3** [41], 4-(diphenylamino)benzaldehyde **D6 [42]**, 4-(*bis*(4-bromophenyl)-amino)benzaldehyde **D7** [43], and 9-methyl-9*H*-carbazole-3-carbaldehyde **D8 [44],** 3-(4-(dimethylamino)phenyl)acrylaldehyde **D9** [45], and 3,3-*bis*(4-(dimethylamino)phenyl)-acrylaldehyde) **D10** [46] were synthesized, as previously reported, without modifications and in similar yields.

### 2.1. Synthesis of the Dyes

#### 2.1.1. 2-Butoxy-4-(Diethylamino)Benzaldehyde **D5**

A mixture of 4-(diethylamino)salicylaldehyde (26.5 g, 137 mmol, M = 193.24 g/mol), 1-bromobutane (21.8 g, 159 mmol) and potassium carbonate (18.9 g, 152.2 mmol) were heated in *N,N*-dimethylformamide (DMF) (250 mL) at reflux overnight. The solution was concentrated before addition of water. Extraction of the product was carried out with diethyl ether. The ethereal solution was washed with several portions of water to remove remaining DMF, dried over MgSO_4_ and concentrated to yield the product as an orange oil (32.1 g, 128.73 mmol, 94% yield). ^1^H NMR (CDCl_3_) δ: 0.91 (t, 3*H*, J = 7.4 Hz), 1.14 (t, 6*H*, J = 7.1 Hz), 1.42–1.48 (m, 2*H*), 1.71–1.78 (m, 2*H*), 3.34 (q, 4*H*, J = 7.1 Hz), 3.96 (t, 2*H*, J = 6.3 Hz), 5.95 (d, 1*H*, J = 2.3 Hz), 6.19 (dd, 1*H*, J = 9.0 Hz, J = 2.3 Hz), 7.63 (d, 1*H*, J = 9.0 Hz), 10.1 (s, 1*H*, CHO); ^13^C NMR (CDCl_3_) δ: 12.6, 13.8, 19.3, 31.2, 44.7, 67.8, 93.2, 104.2, 114.4, 130.1, 153.8, 163.9, and 187.1; HRMS (ESI MS) m/z: theor: 249.1729 found: 249.1733 ([M]^+.^ detected).

#### 2.1.2. 1*H*-Cyclopenta[*b*]Naphthalene-1,3(2*H*)-Dione **EA4**

Diethyl naphthalene-2,3-dicarboxylate (10 g, 38.5 mmol, 1 eq., M = 272.30 g/mol) was suspended in extra dry EtOAc (24 mL) and NaH 95% in oil (2.44 g, 96.4 mmol, 2.5 eq.) was added. The reaction media was refluxed at 105 °C for 5 h. After cooling, the yellow solid was filtered off and thoroughly washed with a mixture of EtOH-Et_2_O 50/50. Treatment of this solid with 200 mL of a 1 M HCl solution under reflux for 1.5 h furnished a new solid. After cooling, the solid was filtered off, washed with water and recrystallized in toluene (200 mL). The product was obtained as a brown solid (6.9 g, 35.27 mmol, 91% yield). ^1^H NMR (CDCl_3_) δ: 3.38 (s, 2H), 7.66–7.75 (m, 2*H*), 8.10–8.13 (m, 2*H*), 8.52 (s, 2*H*); ^13^C NMR (CDCl_3_) δ: 46.7, 124.3, 129.7, 130.6, 136.4, 138.2, and 197.6; HRMS (ESI MS) m/z: theor: 196.0524 found: 196.0526 ([M]^+.^ detected).

### 2.2. General Procedure for the Synthesis of **PP1**–**PP10**:

1*H*-Cyclopenta[b]naphthalene-1,3(2*H*)-dione **EA4** (0.5 g, 2.55 mmol) and the appropriate substituted benzaldehyde (2.55 mmol, 1. eq.) were dissolved in absolute ethanol (50 mL) and a few drops of piperidine were added. The reaction mixture was refluxed and progress of the reaction was followed by thin layer chromatography (TLC). After cooling, a precipitate formed. It was filtered off, washed several times with ethanol and dried under vacuum.

#### 2.2.1. 2-(4-(Dodecyloxy)Benzylidene)-1*H*-Cyclopenta[b]Naphthalene-1,3(2*H*)-Dione **PP1**

4-(Dodecyloxy)benzaldehyde (0.74 g, 2.55 mmol), m_exp_ = 1.05 g, 2.24 mmol, 88% yield. ^1^H NMR (CDCl_3_) δ: 0.88 (t, 3*H*, J = 6.2 Hz), 1.27–1.40 (m, 16*H*), 1.46 (qt, 2*H*, J = 7.5 Hz), 1.83 (qt, 2*H*, J = 6.7 Hz), 4.09 (t, 2*H*, J = 6.5 Hz), 7.02 (d, 2*H*, J = 8.7 Hz), 7.67–7.69 (m, 2*H*), 7.94 (s, 1*H*), 8.09–8.10 (m, 2*H*), 8.49 (d, 1*H*, J = 3.0 Hz), 8.62 (d, 2*H*, J = 8.7 Hz); ^13^C NMR (CDCl_3_) δ: 14.1, 22.7, 26.0, 29.1, 29.3, 29.55, 29.59, 29.63, 29.66, 31.92, 114.9, 123.8, 123.9, 126.5, 128.2, 129.0, 129.1, 130.4, 130.5, 135.6, 136.3, 136.5, 137.6, 137.7, 148.0, 164.2, 189.4, and 190.7; HRMS (ESI MS) m/z: theor: 468.2664 found: 468.2665 ([M]^+.^ detected).

#### 2.2.2. 2-(3,4-Dibutoxybenzylidene)-1*H*-Cyclopenta[*b*]Naphthalene-1,3(2*H*)-Dione **PP2**

3,4-Dibutoxybenzaldehyde (0.64 g, 2.55 mmol), m_exp_ = 0.92 g, 2.15 mmol, 84% yield. ^1^H NMR (CDCl_3_) δ: 1.01 (t, 3*H*, J = 7.6 Hz), 1.04 (t, 3*H*, J = 7.5 Hz), 1.50–1.64 (m, 4*H*), 1.82–1.94 (m, 4*H*), 4.14 (t, 2*H*, J = 6.5 Hz), 4.26 (t, 2*H*, J = 6.4 Hz), 6.97 (d, 1*H*, J = 8.4 Hz), 7.66–7.69 (m, 2*H*), 7.85 (d, 1*H*, J = 7.1 Hz), 7.92 (s, 1*H*), 8.07–8.10 (m, 2*H*), 8.49 (s, 2*H*), 8.83 (s, 1*H*); ^13^C NMR (CDCl_3_) δ: 13.8, 13.9, 19.2, 19.3, 31.0, 31.2, 68.8, 68.9, 112.1, 117.8, 123.8, 123.9, 126.9, 128.0, 129.0, 129.1, 130.4, 130.5, 131.7, 135.6, 136.3, 136.5, 137.6, 148.7, 148.8, 154.7, 189.5, and 190.7; HRMS (ESI MS) m/z: theor: 428.1988 found: 428.1987 ([M]^+.^ detected).

#### 2.2.3. 2-(2,4-Dibutoxybenzylidene)-1*H*-Cyclopenta[*b*]Naphthalene-1,3(2*H*)-Dione **PP3**

2,4-Dibutoxybenzaldehyde (0.64 g, 2.55 mmol), m_exp_ = 0.95 g, 2.22 mmol, 87% yield. ^1^H NMR (CDCl_3_) δ: 1.00 (t, 3H, J = 7.4 Hz), 1.04 (t, 3H, J = 7.4 Hz), 1.49–1.63 (m, 4*H*), 1.82 (qt, 2*H*, J = 7.7 Hz), 1.91 (qt, 2*H*, J = 6.4 Hz), 4.09 (t, 4*H*, J = 6.2 Hz), 6.44 (d, 1*H*, J = 0.9 Hz), 6.64 (dd, 1*H*, J = 11.0 Hz, J = 0.9 Hz), 7.64–7.67 (m, 2*H*), 8.06–8.09 (m, 2*H*), 8.44 (s, 2*H*), 8.62 (s, 1*H*), 9.37 (d, 1*H*, J = 9.0 Hz); ^13^C NMR (CDCl_3_) δ: 13.77, 13.83, 19.17, 19.32, 31.04-31.13, 68.2, 68.7, 98.8, 106.2, 116.5, 123.44, 123.48, 127.1, 128.7, 128.8, 130.3, 130.4, 135.8, 136.3, 136.4, 136.9, 137.7, 142.4, 163.1, 166.6, 189.5, and 191.0; HRMS (ESI MS) m/z: theor: 428.1988 found: 428.1986 ([M]^+.^ detected).

#### 2.2.4. 2-(4-(Dimethylamino)Benzylidene)-1*H*-Cyclopenta[*b*]Naphthalene-1,3(2*H*)-Dione **PP4**

4-Dimethylaminobenzaldehyde (0.38 g, 2.55 mmol), m_exp_ = 0.62 g, 1.89 mmol, 74% yield. ^1^H NMR (CDCl_3_) δ: 3.16 (s, 6*H*), 6.74 (d, 2*H*, J = 9.0 Hz), 7.62–7.64 (m, 2*H*), 7.87 (s, 1*H*), 8.05–8.06 (m, 2*H*), 8.39 (d, 2*H*, J = 2.6 Hz), 8.61 (d, 2*H*, J = 8.6 Hz); ^13^C NMR (CDCl_3_) δ: 40.1, 111.5, 122.4, 122.9, 123.1, 125.1, 128.5, 128.6, 130.2, 130.3, 135.9, 136.1, 136.3, 137.8, 138.5, 148.5, 154.3, 189.6, and 191.4; HRMS (ESI MS) m/z: theor: 327.1259 found: 327.1260 ([M]^+.^ detected).

#### 2.2.5. 2-(2-Butoxy-4-(Diethylamino)Benzylidene)-1*H*-Cyclopenta[b]Naphthalene-1,3(2*H*)-Dione **PP5**

2-Butoxy-4-(diethylamino)benzaldehyde (0.63 g, 2.55 mmol), m_exp_ = 1.02 g, 2.38 mmol, 94% yield. ^1^H NMR (CDCl_3_) δ: 1.04 (t, 3*H*, J = 7.4 Hz), 1.28 (t, 6*H*, J = 7.1 Hz), 1.60 (qt, 2*H*, J = 7.4 Hz), 1.92 (qt, 2*H*, J = 7.8 Hz), 3.50 (q, 4*H*, J = 7.1 Hz), 4.08 (t, 2*H*, J = 6.4 Hz), 6.03 (d, 1*H*, J = 2.0 Hz), 6.43 (dd, 1*H*, J = 9.3 Hz, J = 2.0 Hz), 7.59–7.62 (m, 2H), 8.03–8.05 (m, 2*H*), 8.34 (s, 2*H*), 8.58 (s, 1*H*), 9.50 (d, 1*H*, J = 9.3 Hz); ^13^C NMR (CDCl_3_) δ: 12.8, 13.9, 19.4, 31.1, 45.1, 68.2, 93.1, 105.1, 113.2, 122.37, 122.40, 123.1, 128.1, 128.2, 130.1, 130.2, 136.08, 136.11, 136.3, 137.8, 138.0, 155.2, 164.1, 189.8, and 191.9; HRMS (ESI MS) m/z: theor: 427.2147 found: 427.2149 ([M]^+.^ detected).

#### 2.2.6. 2-(4-(Diphenylamino)Benzylidene)-1*H*-Cyclopenta[*b*]Naphthalene-1,3(2*H*)-Dione **PP6**

4-(Diphenylamino)benzaldehyde (0.70 g, 2.55 mmol), m_exp_ = 1.02 g, 2.26 mmol, 89% yield. ^1^H NMR (CDCl_3_) δ: 7.02 (d, 2*H*, J = 8.9 Hz), 7.20–7.24 (m, 6*H*), 7.38 (t, 4*H*, J = 8.2 Hz), 7.65–7.67 (m, 2*H*), 7.88 (s, 1*H*), 8.07–8.09 (m, 2*H*), 8.44 (d, 2*H*, J = 7.7 Hz), 8.50 (d, 2*H*, J = 8.9 Hz); ^13^C NMR (CDCl_3_) δ: 118.7, 123.50, 123.56, 125.7, 126.0, 126.7, 127.2, 128.8, 128.9, 129.8, 130.3, 130.4, 135.8, 136.3, 136.4, 137.3, 137.7, 145.6, 147.6, 153.1, 189.4, and 191.0; HRMS (ESI MS) m/z: theor: 451.1572 found: 451.1574 ([M]^+.^ detected).

#### 2.2.7. 2-(2-Butoxy-4-(Diethylamino)Benzylidene)-1*H*-Cyclopenta[*b*]Naphthalene-1,3(2*H*)-Dione **PP7**

4-(*Bis*(4-bromophenyl)amino)benzaldehyde (1.1 g, 2.55 mmol, M = 431.13 g/mol), m_exp_ = 1.42 g, 2.33 mmol, 92% yield. ^1^H NMR (CDCl_3_) δ: 7.03–7.08 (m, 6*H*), 7.48 (d, 4*H*, J = 8.7 Hz), 7.66–7.69 (m, 2*H*), 7.88 (s, 1*H*), 8.07–8.11 (m, 2*H*), 8.46–8.52 (m, 4*H*); ^13^C NMR (CDCl_3_) δ: 118.6, 119.8, 123.78, 123.82, 127.1, 127.7, 128.2, 128.98, 129.08, 133.0, 135.7, 136.4, 136.5, 137.0, 137.6, 144.7, 147.0, 151.9, 189.3, and 190.7; HRMS (ESI MS) m/z: theor: 606.9783 found: 606.9787 ([M]^+.^ detected).

#### 2.2.8. 2-((9-Methyl-9H-Carbazol-3-Yl)Methylene)-1*H*-Cyclopenta[*b*]Naphthalene-1,3(2*H*)-Dione **PP8**

9-Methyl-9*H*-carbazole-3-carbaldehyde (0.53 g, 2.55 mmol), m_exp_ = 0.84 g, 2.17 mmol, 85% yield. ^1^H NMR (CDCl_3_) δ: 3.91 (s, 3*H*), 7.37 (t, 1*H*, J = 6.7 Hz), 7.43 (d, 1*H*, J = 7.8 Hz), 7.48 (d, 1*H*, J = 8.7 Hz), 7.53 (t, 1*H*, J = 7.3 Hz), 7.66–7.69 (m, 2*H*), 8.05–8.12 (m, 2*H*), 8.20 (s, 1*H*), 8.28 (d, 1*H*, J = 7.3 Hz), 8.48 (d, 2*H*, J = 7.6 Hz), 8.76 (d, 1*H*, J = 8.0 Hz); ^13^C NMR (CDCl_3_) δ: 29.4, 108.8, 109.2, 120.8, 121.0, 123.2, 123.66, 123.68, 123.7, 125.3, 126.8, 127.5, 128.8, 128.9, 129.0, 130.38, 130.40, 133.9, 135.7, 136.3, 136.5, 137.8, 141.7, 144.5, 149.9, 189.5, and 190.9; HRMS (ESI MS) m/z: theor: 387.1259 found: 387.1263 ([M]^+.^ detected).

#### 2.2.9. 2-(3-(4-(Dimethylamino)Phenyl)Allylidene)-1*H*-Cyclopenta[*b*]Naphthalene-1,3(2*H*)-Dione **PP9**

3-(4-(Dimethylamino)phenyl)acrylaldehyde (0.45 g, 2.55 mmol), m_exp_ = 756 mg, 2.14 mmol, 84% yield. ^1^H NMR (CDCl_3_) δ: 3.10 (s, 6*H*), 6.70 (d, 2H, J = 8.4 Hz), 7.36 (d, 1*H*, J = 15.1 Hz), 7.61–7.75 (m, 4*H*), 7.73 (d, 1*H*, J = 12.3 Hz), 8.03–8.07 (m, 2*H*), 8.34–8.39 (m, 3*H*); ^13^C NMR (CDCl_3_) δ: 40.1, 111.9, 119.8, 122.9, 123.3, 123.8, 126.3, 128.66, 128.72, 130.30, 130.35, 131.7, 136.2, 136.3, 136.5, 137.6, 147.6, 152.8, 154.8, 190.6, and 190.9; HRMS (ESI MS) m/z: theor: 353.1416 found: 353.1414 ([M]^+.^ detected).

#### 2.2.10. 2-(3,3-Bis(4-(Dimethylamino)Phenyl)Allylidene)-1*H*-Cyclopenta[*b*]Naphthalene-1,3(2*H*)-Dione **PP10**

3,3-*Bis*(4-(dimethylamino)phenyl)acrylaldehyde (0.75 g, 2.55 mmol), m_exp_ = 1.06 g, 2.24 mmol, 88% yield. ^1^H NMR (CDCl_3_) δ: 3.08 (s, 6*H*), 3.09 (s, 6*H*), 6.68 (d, 2*H*, J = 9.0 Hz), 6.78 (d, 2*H*, J = 8.7 Hz), 7.24 (d, 2*H*, J = 10.2 Hz), 7.50 (d, 2*H*, J = 8.9 Hz), 7.60–7.63 (m, 2*H*), 7.78 (d, 1*H*, J = 12.9 Hz), 8.01–8.06 (m, 2*H*), 8.33 (d, 2*H*, J = 5.2 Hz), 8.42 (d, 2*H*, J = 12.9 Hz); ^13^C NMR (CDCl_3_) δ: 40.1, 40.2, 111.4, 111.5, 119.4, 122.4, 122.7, 125.8, 125.9, 128.3, 128.4, 128.8, 130.2, 132.5, 133.7, 136.1, 136.3, 136.7, 137.8, 146.6, 151.9, 152.4, 167.0, 191.0, and 191.1; HRMS (ESI MS) m/z: theor: 472.2151 found: 472.2148 ([M]^+.^ detected).

### 2.3. General Procedure for the Synthesis of **PP11**–**PP20**

Indane-1,3-dione (1*H*-indene-1,3(2*H*)-dione) **EA1** (0.37 g, 2.55 mmol) and the appropriate substituted benzaldehyde (2.55 mmol, 1 eq.) were dissolved in absolute ethanol (50 mL) and a few drops of piperidine were added. The reaction mixture was refluxed and progress of the reaction was followed by TLC. After cooling, a precipitate formed in most of the case. This latter was filtered off, washed several times with ethanol and dried under vacuum. For several chromophores (**PP11**, **PP12**, **PP13** or **PP20**), a purification by column chromatography on SiO_2_ was required.

#### 2.3.1. 2-(4-(Dodecyloxy)Benzylidene)-1*H*-Indene-1,3(2*H*)-Dione **PP11**

4-(Dodecyloxy)benzaldehyde (0.74 g, 2.55 mmol), m_exp_ = 790 mg, 1.89 mmol, 74% yield. ^1^H NMR (CDCl_3_) δ: 0.88 (t, 3*H*, J = 6.4 Hz), 1.21–1.54 (m, 18*H*), 1.83 (qt, 2*H*, J = 7.8 Hz), 4.07 (t, 2*H*, J = 6.5 Hz), 7.01 (d, 2*H*, J = 8.9 Hz), 7.77–7.85 (m, 2*H*), 7.85 (s, 1*H*), 7.97–8.00 (m, 2*H*), 8.54 (d, 2*H*, J = 8.9 Hz); ^13^C NMR (CDCl_3_) δ: 14.1, 22.7, 26.0, 29.1, 29.3, 29.54, 29.58, 29.62, 29.64, 31.9, 68.5, 114.9, 123.0, 126.3, 126.4, 134.8, 135.0, 137.3, 140.0, 142.4, 147.0, 163.8, 189.6, and 190.9; HRMS (ESI MS) m/z: theor: 418.2508 found: 418.2503 ([M]^+.^ detected).

#### 2.3.2. 2-(3,4-Dibutoxybenzylidene)-1*H*-Indene-1,3(2*H*)-Dione **PP12**

3,4-Dibutoxybenzaldehyde (0.64 g, 2.55 mmol), m_exp_ = 820 mg, 2.17 mmol, 85% yield. ^1^H NMR (CDCl_3_) δ: 1.00 (t, 3*H*, J = 7.5 Hz), 1.02 (t, 3*H*, J = 7.5 Hz), 1.48–1.63 (m, 4*H*), 1.83–1.93 (m, 4*H*), 4.12 (t, 2*H*, J = 6.6 Hz), 4.23 (t, 2*H*, J = 6.4 Hz), 6.95 (d, 1*H*, J = 8.5 Hz), 7.77–7.82 (m, 4*H*), 7.96–8.00 (m, 2*H*), 8.72 (d, 1*H*, J = 1.7 Hz); ^13^C NMR (CDCl_3_) δ: 13.8, 13.9, 19.2, 19.3, 31.0, 31.2, 68.8, 68.9, 112.1, 117.6, 123.0, 126.2, 126.6, 131.2, 134.7, 134.9, 140.0, 142.5, 147.6, 148.8, 154.4, 189.7, and 190.9; HRMS (ESI MS) m/z: theor: 378.1831 found: 378.1832 ([M]^+.^ detected).

#### 2.3.3. 2-(2,4-Dibutoxybenzylidene)-1*H*-Indene-1,3(2*H*)-Dione **PP13**

2,4-Dibutoxybenzaldehyde (0.64 g, 2.55 mmol), m_exp_ = 723 mg, 1.91 mmol, 75% yield. ^1^H NMR (CDCl_3_) δ: 1.00 (t, 3*H*, J = 7.4 Hz), 1.02 (t, 3*H*, J = 7.3 Hz), 1.46–1.64 (m, 6*H*), 1.76–1.94 (m, 4*H*), 4.07 (t, 2*H*, J = 6.4 Hz), 4.08 (t, 2*H*, J = 6.4 Hz), 6.42 (d, 1*H*, J = 2.2 Hz), 6.61 (dd, 1*H*, J = 9.0 Hz, J = 2.2 Hz), 7.73–7.76 (m, 2*H*), 7.94–7.97 (m, 2*H*), 8.51 (s, 1*H*), 9.22 (d, 1*H*, J = 9.0 Hz); ^13^C NMR (CDCl_3_) δ: 13.7, 13.8, 19.2, 31.1, 31.2, 68.2, 68.6, 98.8, 106.1, 116.1, 122.7, 122.8, 125.2, 134.5, 124.6, 136.5, 140.0, 141.3, 142.3162.7, 166.1, 189.8, and 191.2; HRMS (ESI MS) m/z: theor: 378.1831 found: 378.1834 ([M]^+.^ detected)

#### 2.3.4. 2-(4-(Dimethylamino)Benzylidene)-1*H*-Indene-1,3(2*H*)-Dione **PP14**

4-Dimethylaminobenzaldehyde (0.38 g, 2.55 mmol), m_exp_ = 580 mg, 2.09 mmol, 82% yield). ^1^H NMR (CDCl_3_) δ: 3.14 (s, 6*H*), 6.75 (d, 2*H*, J = 9.2 Hz), 7.70–7.75 (m, 2*H*), 7.78 (s, 1*H*), 7.90–7.94 (m, 2*H*), 8.54 (d, 2*H*, J = 9.2 Hz); ^13^C NMR (CDCl_3_) δ: 40.1, 111.4, 122.1, 122.48, 122.49, 123.1, 134.1, 134.3, 137.9, 139.9, 142.3, 147.5, 154.0, 190.0, and 191.7; HRMS (ESI MS) m/z: theor: 277.1103 found: 277.1105 ([M]^+.^ detected).

#### 2.3.5. 2-(2-Butoxy-4-(Diethylamino)Benzylidene)-1*H*-Indene-1,3(2*H*)-Dione **PP15**

2-Butoxy-4-(diethylamino)-benzaldehyde (0.64 g, 2.55 mmol), m_exp_ = 886 mg, 2.35 mmol, 92% yield. ^1^H NMR (CDCl_3_) δ: 1.03 (t, 3*H*, J = 7.4 Hz), 1.26 (t, 6*H*, J = 7.1 Hz), 1.60 (qt, 2*H*, J = 7.4 Hz), 1.90 (qt, 2*H*, J = 8.0 Hz), 3.48 (q, 4*H*, J = 7.1 Hz), 4.06 (t, 2*H*, J = 6.4 Hz), 6.03 (d, 1*H*, J = 1.7 Hz), 6.40 (dd, 1*H*, J = 9.3 Hz, J= 1.7 Hz), 7.66–7.70 (m, 2H), 7.85–7.89 (m, 2*H*), 8.48 (s, 1*H*), 9.35 (d, 1*H*, J = 9.3 Hz); ^13^C NMR (CDCl_3_) δ: 12.8, 13.9, 19.4, 31.1, 45.0, 68.2, 93.2, 104.9, 112.5, 121.1, 122.1, 122.2, 133.6, 133.9, 137.3, 139.9, 141.1, 142.2, 154.7, 163.7, 190.3, and 192.2; HRMS (ESI MS) m/z: theor: 377.1991 found: 377.1992 ([M]^+.^ detected).

#### 2.3.6. 2-(4-(Diphenylamino)Benzylidene)-1*H*-Indene-1,3(2*H*)-Dione **PP16**

4-(Diphenylamino)benzaldehyde (0.70 g, 2.55 mmol) m_exp_ = 890 mg, 2.22 mmol, 87% yield. ^1^H NMR (CDCl_3_) δ: 7.01 (d, 2*H*, J = 8.9 Hz), 7.18-7.22 (m, 6*H*), 7.34–7.38 (m, 4*H*), 7.74–7.76 (m, 2*H*), 7.78 (s, 1*H*), 7.94–7.96 (m, 2*H*), 8.41 (d, 2*H*, J = 8.9 Hz); ^13^C NMR (CDCl_3_) δ: 118.9, 122.81, 122.84, 125.4, 125.5, 125.8, 126.6, 129.7, 134.5, 134.7, 136.8, 140.0, 142.4, 145.8, 146.5, 152.7, 189.6, and 191.2; HRMS (ESI MS) m/z: theor: 401.1416 found: 401.1418 ([M]^+.^ detected).

#### 2.3.7. 2-(4-(Bis(4-Bromophenyl)Amino)Benzylidene)-1*H*-Indene-1,3(2*H*)-Dione **PP17**

4-(bis(4-bromophenyl)amino)benzaldehyde (1.1 g, 2.55 mmol), m_exp_ = 1.15 g, 2.06 mmol, 81% yield. ^1^H NMR (CDCl_3_) δ: 7.02–7.07 (m, 6*H*), 7.46 (d, 4*H*, J = 8.8 Hz), 7.76–7.78 (m, 3*H*), 7.96–7.98 (m, 2*H*), 8.42 (d, 2*H*, J = 8.9 Hz); ^13^C NMR (CDCl_3_) δ: 118.4, 120.0, 122.98, 123.01, 126.4, 126.9, 127.6, 133.0, 134.7, 135.0, 136.6, 140.1, 142.4, 144.8, 146.0, 151.5, 189.5, and 190.9; HRMS (ESI MS) m/z: theor: 556.9626 found: 556.9628 ([M]^+.^ detected).

#### 2.3.8. 2-((9-Methyl-9H-Carbazol-3-Yl)Methylene)-1*H*-Indene-1,3(2*H*)-Dione **PP18**

9-Methyl-9*H*-carbazole-3-carbaldehyde (0.54 g, 2.55 mmol), m_exp_ = 671 mg, 1.99 mmol, 78% yield; ^1^H NMR (CDCl_3_) δ: 3.91 (s, 3*H*), 7.35 (t, 1*H*, J = 7.5 Hz), 7.43–7.48 (m, 2*H*), 7.54 (t, 1*H*, J = 7.3 Hz), 7.76–7.81 (m, 2*H*), 7.97–8.03 (m, 2*H*), 8.25 (d, 2*H*, J = 7.7 Hz), 8.69 (d, 1*H*, J = 8.7 Hz); ^13^C NMR (CDCl_3_) δ: 29.4, 108.7, 109.1, 120.7, 121.0, 122.9, 123.2, 123.6, 125.0, 125.6, 126.8, 128.6, 133.4, 134.6, 134.8, 140.0, 141.7, 142.5, 144.2, 148.8, 189.8, and 191.2; HRMS (ESI MS) m/z: theor: 337.1103 found: 337.1101 ([M]^+.^ detected).

#### 2.3.9. 2-(3-(4-(Dimethylamino)Phenyl)Allylidene)-1*H*-Indene-1,3(2*H*)-Dione **PP19**

3-(4-(Dimethylamino)-phenyl)acrylaldehyde (0.45 g, 2.55 mmol), m_exp_ = 688 mg, 2.27 mmol, 89% yield. ^1^H NMR (CDCl_3_) δ: 3.09 (s, 6*H*), 6.69 (d, 2*H*, J = 8.9 Hz), 7.31 (d, 1*H*, J = 15.1 Hz), 7.59 (d, 2*H*, J = 8.4 Hz), 7.64 (d, 1*H*, J = 12.3 Hz), 7.71–7.74 (m, 2*H*), 7.90–7.92 (m, 2*H*), 8.28 (dd, 1*H*, J = 15.1 Hz, J = 12.3 Hz); ^13^C NMR (CDCl_3_) δ: 40.1, 111.9, 119.4, 122.4, 122.6, 123.8, 124.5, 131.3, 134.3, 134.4, 140.8, 142.1, 146.5, 152.5, 153.6, 190.8, and 191.2; HRMS (ESI MS) m/z: theor: 303.1259 found: 303.1255 ([M]^+.^ detected).

#### 2.3.10. 2-(3,3-*Bis*(4-(Dimethylamino)Phenyl)Allylidene)-1*H*-Indene-1,3(2*H*)-Dione **PP20**

3-(4-(Dimethylamino)phenyl)-acrylaldehyde (0.75 g, 2.55 mmol), m_exp_ = 915 mg, 2.16 mmol, 85% yield. ^1^H NMR (CDCl_3_) δ: 3.06 (s, 6*H*), 3.07 (s, 6*H*), 6.67 (d, 2*H*, J = 9.0 Hz), 6.76 (d, 2*H*, J = 8.8 Hz), 7.20 (d, 2*H*, J = 8.7 Hz), 7.45 (d, 2*H*, J = 9.0 Hz), 7.67–7.71 (m, 3*H*), 7.84–7.89 (m, 2*H*), 8.28 (d, 1*H*, J = 12.8 Hz); ^13^C NMR (CDCl_3_) δ: 40.1, 40.2, 111.4, 111.5, 118.8, 122.1, 122.3, 123.9, 125.7, 128.8, 130.7, 132.1, 132.7, 133.4, 133.9, 134.1, 140.7, 142.0, 145.5, 151.7, 152.1, 165.5, 191.3, and 191.5; HRMS (ESI MS) m/z: theor: 422.1994 found: 422.1998 ([M]^+.^ detected).

## 3. Results and Discussion

### 3.1. Synthesis of the Dyes and Electron Acceptors

**Figure 3:**
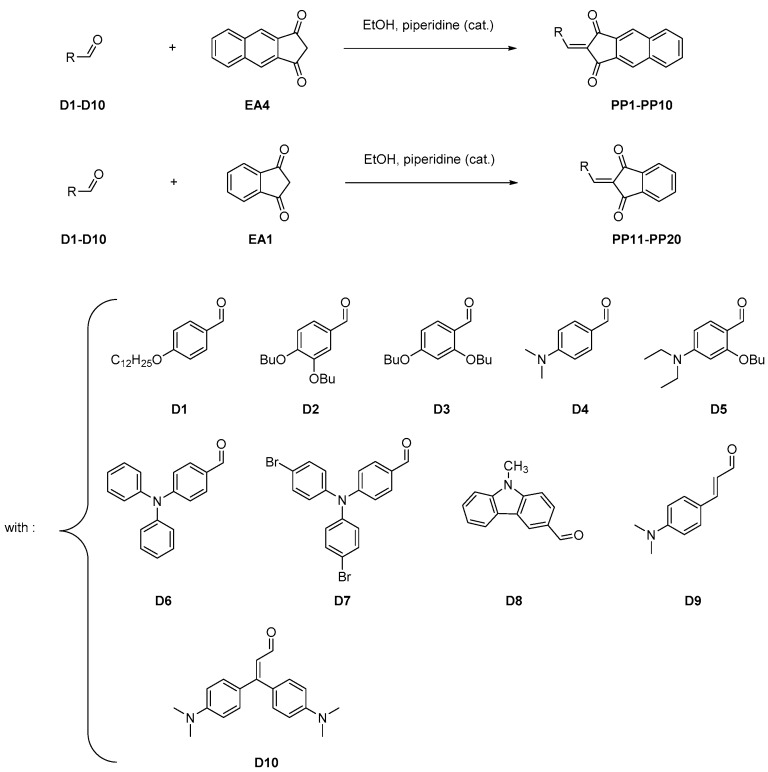
Synthetic pathways to **PP1**–**PP20** and the ten aldehydes used in this study.

All dyes **PP1**–**PP20** presented in this work have been synthesized by a Knoevenagel reaction involving the aldehydes **D1**–**D10** and the two electron acceptors **EA4** and **EA1**, respectively (See Figure 3). The different reactions were performed in ethanol using piperidine as the catalyst. **PP1**–**PP20** were obtained with reaction yields ranging from 74% yield for **PP4** and **PP11** to 94% for **PP5** (see Table 1). All compounds were obtained as solids and they were characterized by ^1^H, ^13^C NMR spectroscopies, and HRMS spectrometry (see Supplementary Materials). It has to be noticed that the synthetic procedure to **EA4** has been greatly improved compared to that reported in the literature [47], enabling to reach a reaction yield of 91%. Indeed, a common method to synthesize 1,3-indanedione derivatives consists in a Claisen condensation of the corresponding diesters with ethyl acetate under basic conditions (generally sodium hydride).

**Figure 4:**
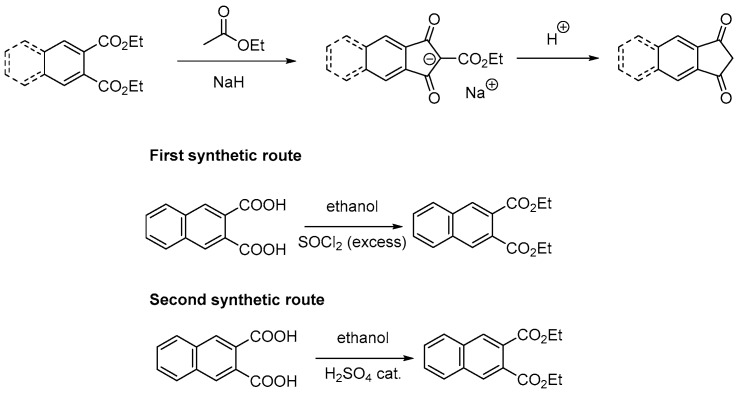
Synthetic route to 1,3-indanedione derivatives by the Claisen condensation and the two possible esterification procedures for naphthalene-2,3-dicarboxylic acid.

The final product is obtained after the condensation step by decarboxylation of the intermediate salt under hot acidic conditions (see Figure 4). To access the starting compound, i.e., diethyl naphthalene-2,3-dicarboxylate, two different reaction pathways were examined, by esterification of naphthalene-2,3-dicarboxylic acid in ethanol in the presence of an excess of thionyl chloride, or by the classical esterification conditions consisting in refluxing the acid in ethanol in the presence of a catalytic amount of H_2_SO_4_ (see Figure 4). If diethyl naphthalene-2,3-dicarboxylate could be obtained in almost quantitative yields with the two procedures, our attempt to convert the diester obtained by the first procedure were unfruitful to form **EA4**. This is attributable to remaining traces of SOCl_2_ in the diester, despites the numerous washings in basic conditions. Due to the presence of water traces in ethyl acetate, SOCl_2_ could react with water to form HCl, neutralizing part of the NaH introduced. Conversely, in the second procedure, H_2_SO_4_ can be easily removed from the diester due to its catalytic use, avoiding this drawback. Using the oil obtained by this second procedure, a major improvement was obtained by replacing NaH 60% dispersion in oil by NaH 95% dispersion in oil for the first reaction step.

By using this more concentrated dispersion, the reaction yield of the crude materials for the two steps (the intermediate salt was not isolated) could be greatly increased. By modifying the decarboxylation time (increased from 1.5 h instead of 20 min), the quantity of NaH (2.5 eq. instead of 1.45 eq.) and the recrystallization solvent [47] (benzene replaced by the less toxic toluene) compared to that reported in the literature, the overall reaction yield after purification could be increased from 65% (literature) up to 91% in our optimized conditions.

### 3.2. Theoretical Calculations

**Figure 5:**
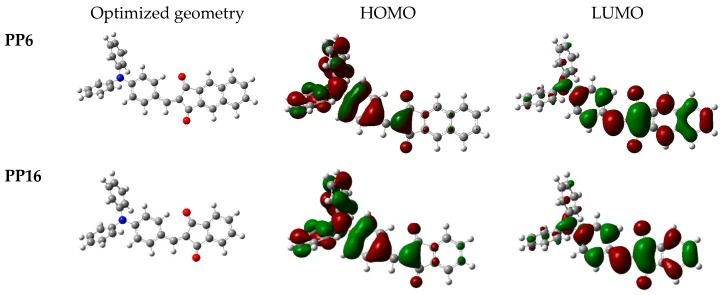
Optimized geometries and HOMO/LUMO electronic distributions of **PP6** and **PP16**.

**Figure 6:**
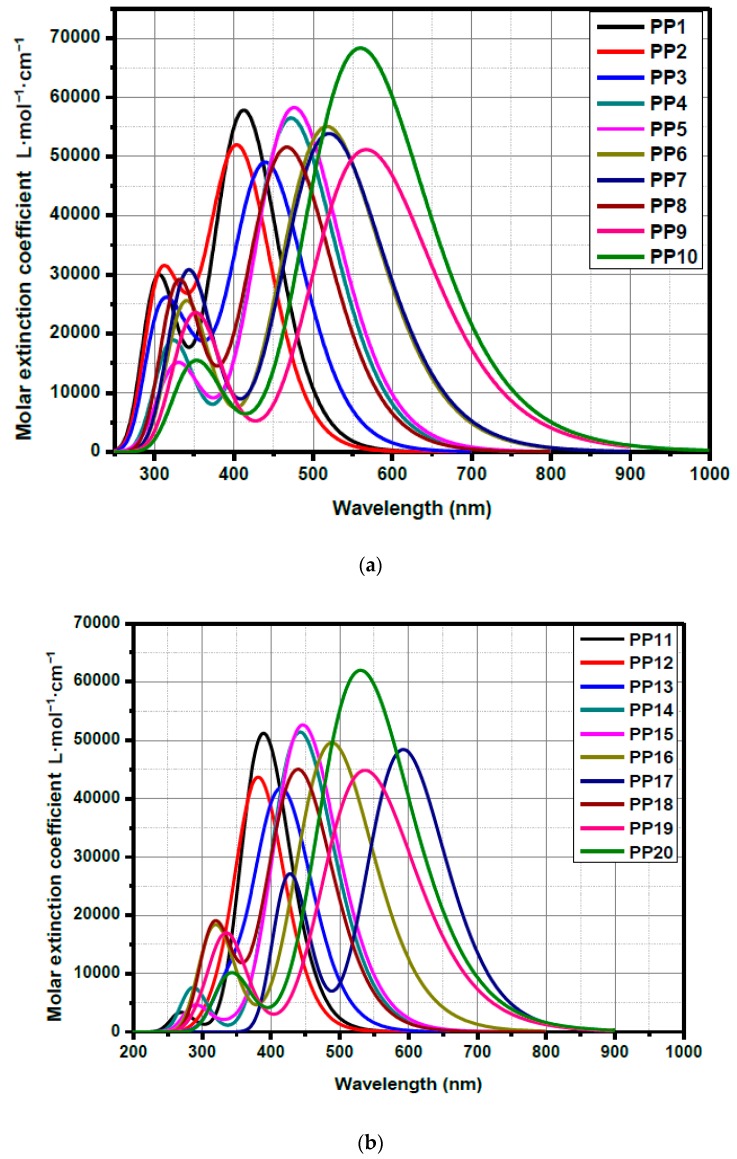
Simulated absorption spectra in dilute dichloromethane (5 × 10^−3^ M) of synthetized compounds **PP1**–**PP10** (**a**) and **PP11**–**PP20** (**b**).

Theoretical studies were realized in order to investigate the energy levels as well as the molecular orbitals (M.O.) compositions of the different dyes. DFT calculations of all synthetized compounds were performed by the B3LYP/6-311G(d,p) level of theory using Gaussian 09 programs. Dichloromethane as the solvent and the polarizable continuum model (PCM) as the solvent model were used for the TD-DFT calculations. The optimized geometries as well as the highest occupied molecular orbitals (HOMO) and the lowest unoccupied molecular orbital (LUMO), i.e., the frontier orbitals’ electronic distributions of selected compounds (**PP6** and **PP16**) are given in Figure 5. The data for all compounds are supplied in the Supplementary Materials. The simulated absorption spectra are shown in the Figure 6 while the Table 2 summarizes all HOMO and LUMO energy levels and electronic transitions associated with the intramolecular charge transfer (ITC) absorption peaks. The assignment of the electronic transitions for λ_max_ reported in the Table 2 has been determined with GaussSum 3.0 software, and especially, the contribution of the different transitions.

As can be observed for all compounds, clear common trends are well observed for the two series:

● ICT Absorption:

The absorption peaks corresponded to the ICT are well observed in the Figure 6 and the values of maximal ICT absorption wavelengths are given in the Table 2. A clear trend is observed in both series: The ICT transition is mainly associated with the HOMO-LUMO transition. When the electron donor properties of the donor part increase, a clear redshift is obtained for the ICT transition. When we compare the two molecules in the two series with the same donor part, we observe that the ICT of the **EA4**-based molecule is redshift compared to that of **EA1**-based counterpart (for instance: λ_max_ (nm) = 518 and 490 for **PP6** and **PP16**, respectively, both compounds have triphenylamine as donor part).

● HOMO and LUMO Energy Levels: 

Within the same series, the values of E_HOMO_ importantly vary as a function of the electron donating capacity of the donor part while the values of E_LUMO_ slightly change. When we compare cross the two series: two molecules with the same donor part, we observe that they have nearly the same E_HOMO_ while the E_LUMO_ of the **EA4**-based molecule is deeper compared to that of **EA1**-based counterpart, which is in good accordance with the more electron deficient nature of **EA4** (for instance: E_HOMO_ (eV) = −5.633 and −5.635 for **PP6** and **PP16**, respectively, while E_LUMO_ (eV) = −2.836 and −2.694, respectively. Both compounds have triphenylamine as donor part). 

● HOMO and LUMO Orbitals Distribution:

The HOMO orbitals are well developed over the electron donor part while the LUMO orbitals are on the electron deficient moiety. Only a small overlap between the HOMO and LUMO orbitals is detected (see Figure 5).

### 3.3. Optical Properties

**Figure 7:**
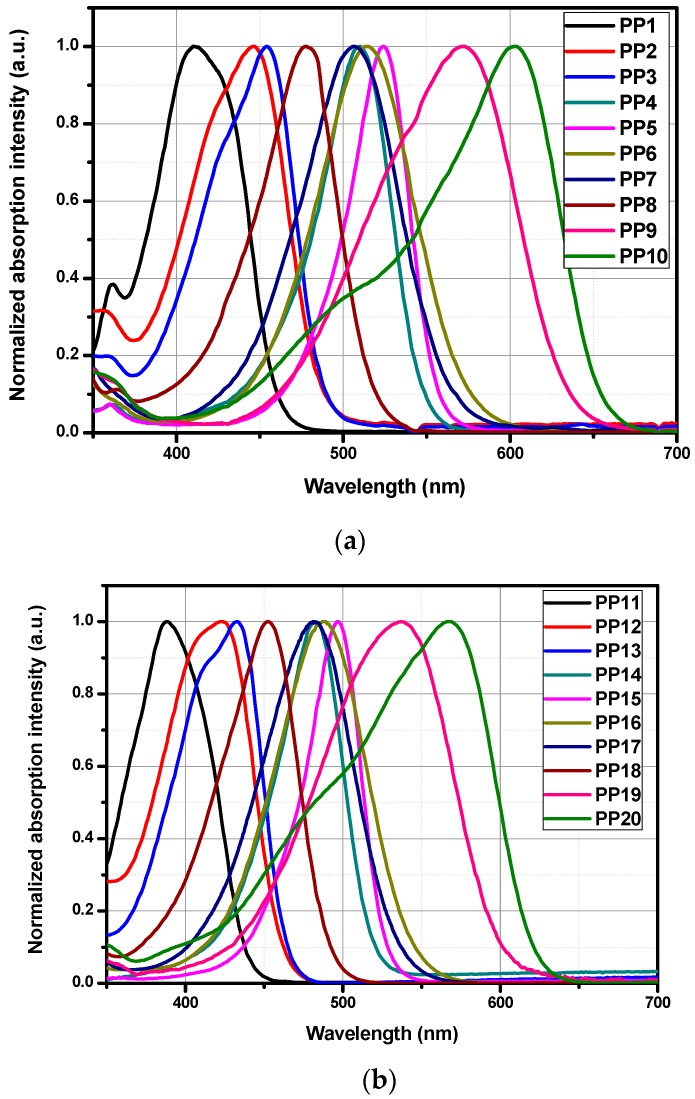
UV-visible absorption spectra of **PP1**–**PP10** (**a**) and **PP11**–**PP20** (**b**) in dichloromethane.

All compounds were characterized by UV-visible spectroscopy and their absorption spectra in dichloromethane are provided in the Figure 7. **PP1**–**PP20** are push-pull compounds that possess an electron donating group and an electron accepting group which interact by mean of a π-conjugated system. The position of the charge transfer band will depend of parameters such as the strength of the electron donating/accepting groups, but also of the length of the π-conjugated system. By combining both effects, a decrease in the energy difference between the HOMO and the LUMO can be obtained, resulting in a shift of the absorption spectrum towards longer wavelengths.

**Figure 8:**
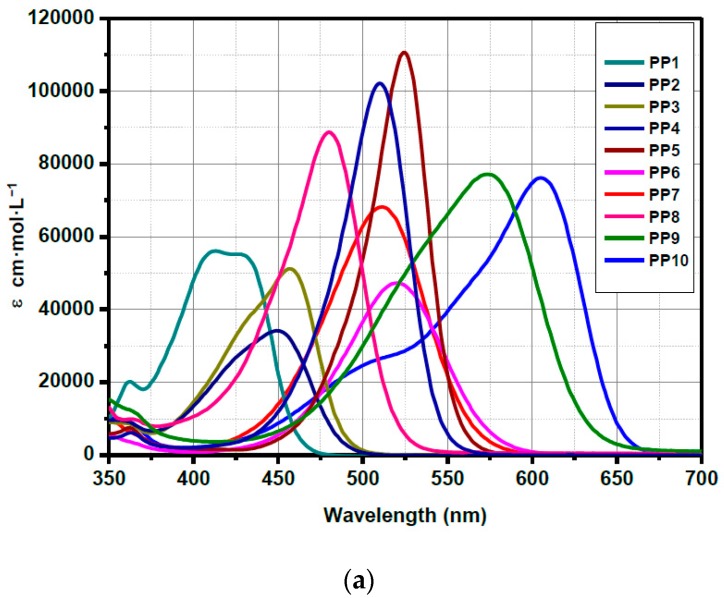

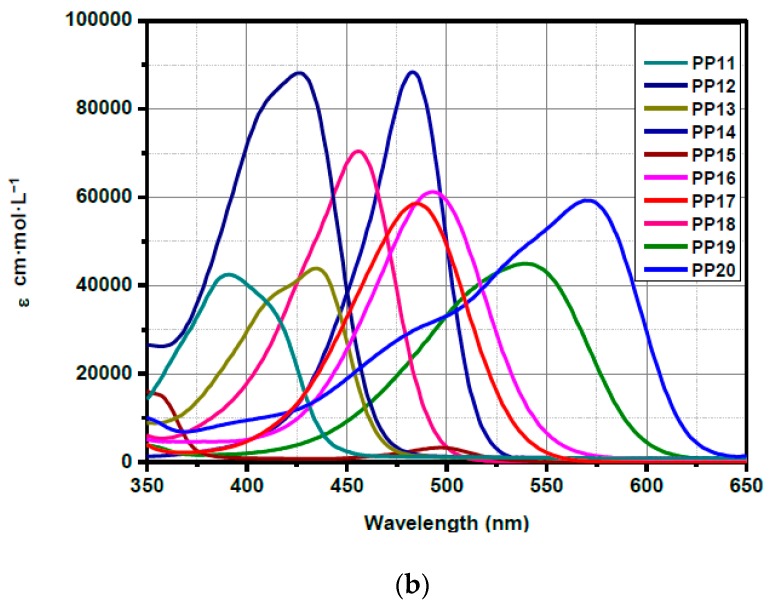
UV-visible absorption spectra of **PP1**–**PP10** (**a**) and **PP11**–**PP20** (**b**) in chloroform.

While examining the first series **PP1**–**PP10**, absorption maxima ranging from 412 nm for **PP1** to 524 nm for **PP5** were found in dichloromethane for dyes exhibiting a short spacer. Elongation of the π-conjugated system resulted in a significant red-shift of the absorption maximum, shifting from 471 nm for **PP1** to 571 nm for **PP9**. The most red-shifted absorption was found for **PP10**, peaking at 603 nm. While comparing the **PP1**–**PP10** series with **PP11**–**PP20** based on 1,3-indanedione, absorption spectra of **PP11**–**PP20** were found to follow the same order than that of the **PP1**–**PP10** series, but with an absorption blue-shifted by about 30 nm (see Figure 7). Examination of the molar extinction coefficients for the two series also revealed the **PP1**–**PP10** series to exhibit higher molar extinction coefficients than that of the **PP11**–**PP20** series, consistent with an improvement of the molar absorptivity with the oscillator strength and the conjugation extension (see Figure 8) [48].

The experimental absorption spectra recorded in dichloromethane of all compounds are in good accordance with predicted properties obtained by DFT calculation. Notably, a good accordance between the theoretical absorption maxima can be found for all dyes (See Table 2, Table 3 and Table 4). Second, considering that the main absorption band was theoretically determined for all dyes originating from a HOMO->LUMO transition, this latter can thus be confidently assigned to the intramolecular charge transfer bands for all dyes. A contribution of the HOMO->LUMO transition to the ICT band ranging between 85% (for **PP20**) to 100% for **PP9** and **PP19** could be calculated. 

### 3.4. Solvatochromism

All dyes **PP1**–**PP20** exhibited a good solubility in most of the common organic solvents so that examination of the solvatochromism could be carried out in 23 solvents of different polarities. It has to be noticed that alcohols such as methanol, ethanol, propan-2-ol, butan-1-ol and pentan-1-ol were initially considered as solvents for the solvatochromic study, but the absorption maxima obtained with these solvents were irregular compared to that obtained with the 23 other solvents. This specific behavior can be assigned to the fact that all dyes precipitated in alcohols such as in ethanol which was the solvent of reaction. Even if the absorption spectra could be recorded in alcohols for all dyes, presence of free molecules and aggregates in solution certainly modify the position of the absorption maxima. A summary of the absorption maxima for the twenty dyes are provided in Table 3 and Table 4. 

As evidenced in the Table 3 and Table 4, analysis of the solvatochromism in solvents of different polarities confirmed the presence of an intramolecular charge transfer in all dyes. Intramolecular nature of the charge transfer was demonstrated by performing successive dilutions, intensity of the charge transfer band linearly decreasing with the dye concentrations. Various empirical polarity scales have been developed over the years to interpret the solvent-solute interaction and the Kamlet-Taft’s [49], Dimroth-Reichardt’s [50], Lippert-Mataga’s [51], Catalan’s [52], Kawski-Chamma-Viallet’s [53], McRae’s [54], Suppan’s [55], and Bakhshiev’s [56] scales can be cited as the most popular ones. Among all scales, the Kamlet-Taft solvent polarity scale proved to be the most adapted one, linear correlations being obtained for all dyes by plotting the absorption maximum vs. the empirical Taft parameters (see linear regression in Supplementary Materials). For all the other polarity scales based on the dielectric constant or the refractive index of solvents, no reasonable correlations could be established. The Kamlet and Taft equation is also a multiparametric equation that can take into account the dipolarity-polarizability (π*), the hydrogen-donating and accepting ability (α and β) of the solvents, modelizing more precisely the interactions between the solvent and the solute. However, multiple linear regression analyses carried out on the triparametric Kamlet-Taft equation using the three solvent descriptors (α, β, π*) did not improve the correlation coefficients, as demonstrated in the Table in SI. It can therefore be concluded that the dipolarity-polarizability of the solvent is the primary cause influencing the position of the ICT band.

**Figure 9:**
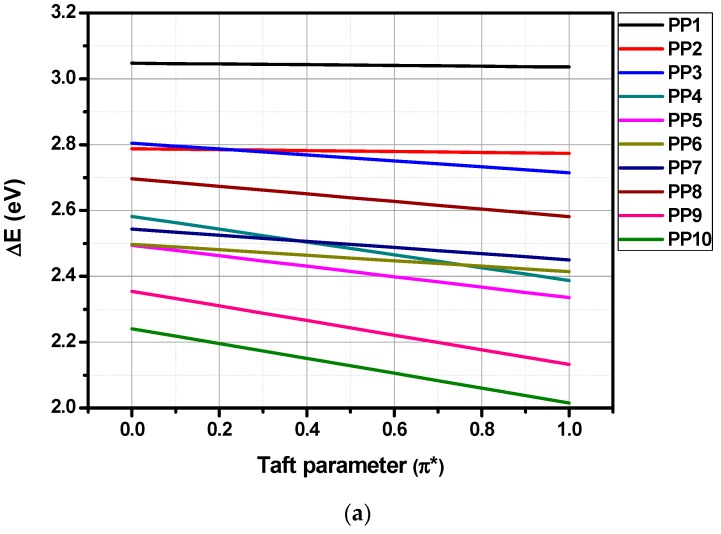

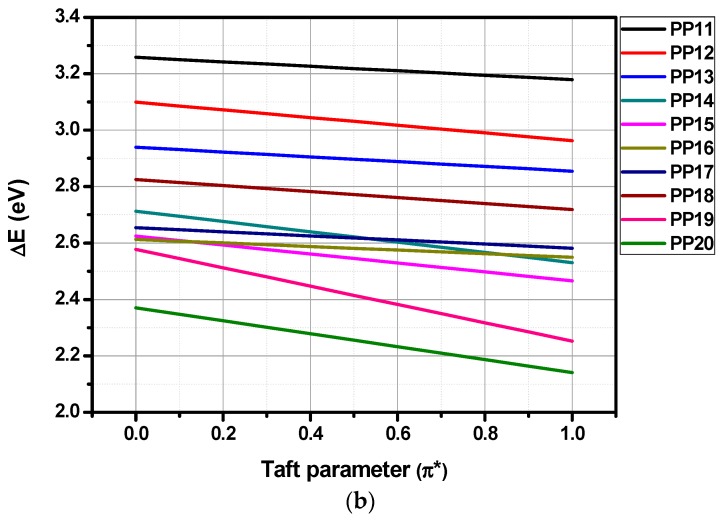
Variation of the positions of the charge transfer band with Kamlet-Taft empirical parameters for **PP1**–**PP10** (**a**) and **PP11**–**PP20** (**b**).

As evidenced in the Figure 9 and Figures in Supplementary Materials, **PP1**–**PP20** show negative slopes with good linear correlations, indicative of a positive solvatochromism. Excepted for **PP1**, **PP2**, **PP11**, and **PP12** that possess weak electron donors, all dyes displayed strong negative slopes, indicative of a significant charge redistribution upon excitation. The most important solvatochromism was found for **PP9**, **PP10**, **PP19**, and **PP20** that exhibit the longest conjugated spacers.

### 3.5. Fluorescence Spectroscopy

**Figure 10:**
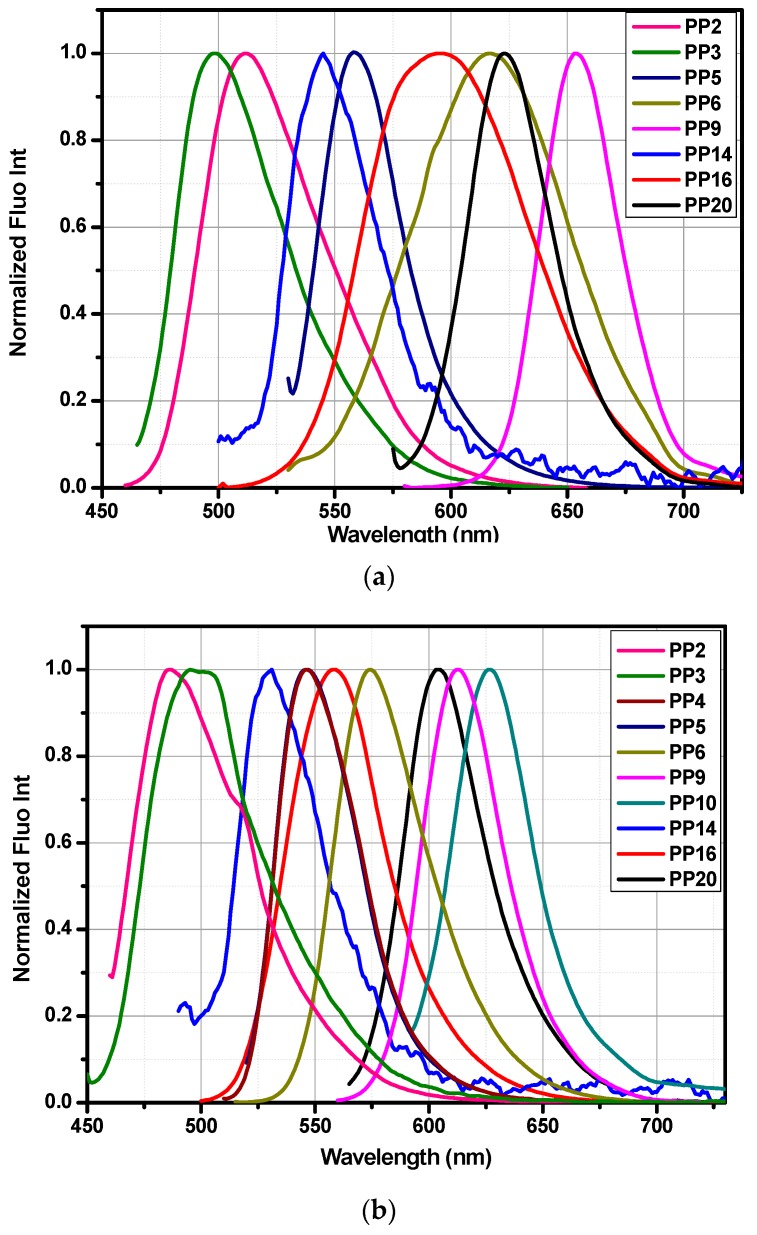
Fluorescence spectra of studied compounds in dichloromethane (**a**) and toluene (**b**).

The fluorescent properties of all compounds were investigated in dichloromethane and toluene, in diluted solutions and the results are summarized in the Figure 10 and the Table 5. Interestingly, most of the chromophores were not emissive whatever the solvent is and this behavior is consistent with results classically reported in the literature for indane-1,3-dione derivatives [57,58,59,60,61]. However, it has to be noticed that a series of indane-1,3-dione derivatives was reported as being highly emissive, in a specific context, by use of oligo(phenylene)vinylene as electron donors [62]. Only eleven of these compounds displayed a weak emission in toluene so that the luminescence lifetime as well as the photoluminescence quantum yield were not determined for these compounds. While examining the emission maxima, the most red-shifted emissions were found for **PP9** and **PP10** displaying the most extended conjugated spacer but also the most extended electron acceptor. As attended, a blue-shifted emission was found for **PP20** (λ_em_ = 604 nm) relative to that of **PP10** (λ_em_ = 626 nm), directly resulting from its blue-shifted absorption maxima. While comparing the results obtained in dichloromethane and toluene, absorption maxima were found to be blue-shifted by *ca.* 20 nm for all dyes in toluene relative to that determined in dichloromethane. Based on the Taft parameters for both solvents (0.54 for toluene and 0.82 for dichloromethane), it can be concluded that a positive solvatochromism can also be observed in emission. This point is notably confirmed by the Stokes shifts determined in both dichloromethane and toluene, which are almost identical.

### 3.6. Electrochemical Properties

**Figure 11:**
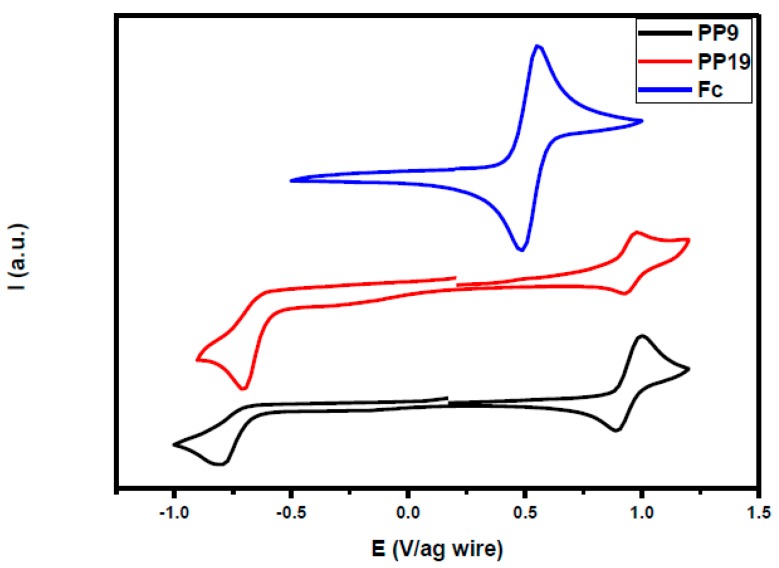
Selected examples of cyclic voltammograms of studied compounds (**PP9**, **PP19**) and Ferrocene (Fc).

The electrochemical properties of all compounds have been investigated by cyclic voltammetry (CV) in dilute solutions, either in acetonitrile or in dichloromethane. The selected voltammograms are shown in the Figure 11 and CV curves of all compounds are given in the Supplementary Materials. The redox potentials of all compounds are summarized in the Table 6 in which redox potentials are given against the half wave oxidation potential of the ferrocene/ferrocenium cation couple.

As shown in Figure 11, **PP9** and **PP19** differ from each other only in the nature of the accepting moiety. As expected, both compounds have quasi-reversible oxidation processes with identical oxidation potentials (Figure 11, Table 6). Indeed, the oxidation process for the two chromophores is centered onto the electron-donating part and this latter is the same for the two dyes. Conversely, the reduction potential of **PP9** comprising 1*H*-cyclopenta[*b*]naphthalene-1,3(2*H*)-dione (**EA4**) as the electron-accepting moiety is slightly lower than that of **PP19** (comprising **EA1** as the acceptor), leading to narrower electrochemical bandgap. This is in good accordance with the optical bandgap determined by UV-visible absorption spectroscopy, where **PP9** showed a red-shifted ICT band in comparison to **PP19** (See Figure 7 and Figure 8). 

The redox potentials of all other compounds are gathered in the Table 6. As can be seen in the same series (**PP1**–**PP10** and **PP11**–**PP20**) where the nature of the acceptor moiety is identical, their reduction potentials changed very slightly while the oxidation potential importantly vary as a function of the donor moiety.

The number and the substitution position of the alkoxy chains on the phenyl ring slightly influence the redox potentials of the targeted molecules (**PP1**–**3** and **PP11**–**13**). However, important variations were determined when the electron-donating ability of the electron donor was increased by the presence of diakylamino groups on the phenyl ring (see **PP4**–**5** and **PP14**–**15**). This phenomenon was even much more pronounced when a double bond was inserted between the donor and the acceptor moiety leading to more conjugated push pull molecules (see **PP9**–**10** & **PP19**–**20**). The presence of two 4-(*N*,*N*-methylamino)phenyl groups such as in **PP10** and **PP20** has only a negligible impact on the electrochemical property. In fact, while the second 4-(*N*,*N*-methylamino)phenyl group could increase the electron donating ability, examination of the mesomeric forms in **PP10** and **PP20** clearly evidences the two groups not to be able to contribute simultaneously to the electronic delocalization, as previously mentioned in the literature [46]. While comparing the dyes at identical electron donating groups, push pull compounds prepared with **EA4** (**PP1**–**PP10**) have lower reduction potentials than their counterparts **PP11**–**PP20** comprising **EA1** as the electron withdrawing groups. This is in perfect accordance with the higher electron accepting capacity of **EA4**.

**Figure 12:**
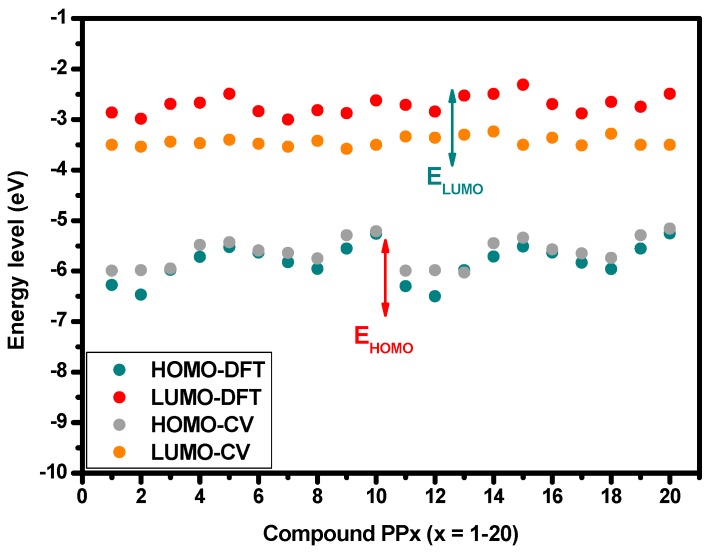
Comparison of frontier orbitals’ energy levels obtained from cyclic voltammetry and DFT calculations.

The redox behaviors of synthesized molecules were then used to estimate their HOMO and LUMO energy levels by using the ferrocene (Fc) ionization potential value (4.8 eV vs. vacuum) as the standard. The correcting factor of 4.8 eV is based on calculations obtained by Pommerehne et al. [63]. It is also important to note that some other correcting factors have also been used in the literature [64]. The obtained values of E_HOMO_ and E_LUMO_ issued from electrochemical characterizations are summarized in the Table 6 for comparison, the optical bandgaps of all dyes in acetonitrile have been added. The Figure 12 shows a comparative presentation of the frontier orbitals’ energy levels experimentally and theoretically obtained. We can see that the experimental findings fit well with the trend predicted by DFT calculation.

## 4. Conclusions

In this work, a series of twenty dyes comprising indane-1,3-dione or 1*H*-cyclopenta[*b*]naphthalene-1,3(2*H*)-dione have been synthesized and examined for their photophysical properties. Introduction of a naphthalene moiety in **EA4** greatly contributed to improve the electron-withdrawing ability of the group. Notably, a red-shift of the intramolecular charge transfer band of ca. 30 nm was observed for all dyes prepared with this acceptor, compared to the parent series comprising **EA1**. A linear and positive solvatochromism was found for all dyes, demonstrating an important charge redistribution upon excitation. A good correlation between the experimental HOMO-LUMO gaps and the theoretical ones could be found. Examination of their electrochemical properties revealed these dyes to be reversibly oxidized whereas an irreversible reduction monoelectronic process was determined for all dyes. By extending the aromaticity of **EA4**, a significant red-shift of the absorption maximum could be obtained for all dyes compared their analogues comprising **EA1**. Future works will consist of further improving the electron accepting ability of **EA4**, which is achievable by functionalizing **EA4** with malononitrile. Based on the present work, the tetracyano derivatives of **EA4** will certainly allow the design of near-infrared dyes that can find applications in research fields such as telecommunications and defense. 

## Figures and Tables

**Table 1:** Reaction yields obtained for the synthesis of **PP1**–**PP20**.

**Compounds**	**PP1**	**PP2**	**PP3**	**PP4**	**PP5**	**PP6**	**PP7**	**PP8**	**PP9**	**PP10**
Reaction yields (%)	88	84	88	74	94	89	92	85	84	88
**Compounds**	**PP11**	**PP12**	**PP13**	**PP14**	**PP15**	**PP16**	**PP17**	**PP18**	**PP19**	**PP20**
Reaction yields (%)	74	85	75	82	92	87	81	78	89	85

**Table 2:** Summary of simulated absorption characteristics in dilute dichloromethane of synthetized compounds. Data were obtained in dichloromethane solution.

Compounds	E_HOMO_ (eV)	E_LUMO_ (eV)	λ_max_ (nm)	Transitions
**PP1**	−6.279	−2.865	414	HOMO->LUMO (95%)
**PP2**	−6.468	−2.983	406	HOMO->LUMO (72%) HOMO-1->LUMO (22%) HOMO-2->LUMO (5%)
**PP3**	−5.974	−2.691	441	HOMO->LUMO (98%)
**PP4**	−5.717	−2.668	473	HOMO->LUMO (99%)
**PP5**	−5.522	−2.489	477	HOMO->LUMO (99%)
**PP6**	−5.633	−2.836	518	HOMO->LUMO (99%)
**PP7**	−5.826	−3.003	520	HOMO->LUMO (99%)
**PP8**	−5.953	−2.816	471	HOMO->LUMO (99%)
**PP9**	−5.554	−2.874	567	HOMO->LUMO (100%)
**PP10**	−5.262	−2.62	574	HOMO->LUMO (90%) HOMO-1->LUMO (10%)
**PP11**	−6.3	−2.71	390	HOMO->LUMO (97%)
**PP12**	−6.498	−2.842	385	HOMO->LUMO (94%) HOMO-1->LUMO (4%)
**PP13**	−5.982	−2.526	417	HOMO->LUMO (97%)
**PP14**	−5.715	−2.498	444	HOMO->LUMO (95%) HOMO->L+1 (4%)
**PP15**	−5.513	−2.309	448	HOMO->LUMO (95%) HOMO->LUMO+1 (4%)
**PP16**	−5.635	−2.694	490	HOMO->LUMO (99%)
**PP17**	−5.835	−2.878	493	HOMO->LUMO (99%)
**PP18**	−5.962	−2.656	443	HOMO->LUMO (98%)
**PP19**	−5.555	−2.748	537	HOMO->LUMO (100%)
**PP20**	−5.253	−2.489	545	HOMO->LUMO (85%) HOMO-1->LUMO (15%)

**Table 3:** Summary of the optical properties of **PP1**–**PP10** in twenty-three solvents and Kamlet and Taft parameters π*.

Compounds	π*^1^	PP1^2^	PP2^2^	PP3^2^	PP4^2^	PP5^2^	PP6^2^	PP7^2^	PP8^2^	PP9^2^	PP10^2^
diethyl ether	0.27	403	442	445	490	505	499	489	464	534	569
toluene	0.54	405	450	441	501	512	509	501	473	553	582
chloroform	0.78	412	450	457	520	524	520	510	480	574	606
THF	0.58	405	444	450	502	517	504	497	471	555	587
1,4-dioxane	0.55	402	443	446	498	512	503	496	468	545	581
acetone	0.71	405	441	449	505	520	501	496	471	562	595
dichloromethane	0.82	411	448	456	510	524	514	505	476	571	603
DMSO	1.00	414	447	458	522	534	512	506	482	590	620
heptane	-0.08	410	445	443	479	497	497	486	461	529	551
acetonitrile	0.75	404	439	450	507	523	505	496	471	565	597
dimethylformamide	0.87	409	447	454	514	528	509	503	478	579	609
ethyl acetate	0.54	404	444	447	498	513	500	491	468	546	579
*p*-xylene	0.43	406	448	448	496	509	504	497	470	547	580
1,2-dichloroethane	0.81	410	447	454	509	524	515	504	474	567	603
dimethylacetamide	0.88	410	447	456	516	529	508	502	477	577	608
diglyme	0.64	406	445	450	506	518	504	497	474	567	593
cyclohexane	0.00	412	446	445	482	499	502	492	462	532	556
triethylamine	0.14	439	454	450	486	501	503	507	463	533	559
hexane	-0.08	409	444	442	477	495	497	488	457	525	551
anisole	0.73	413	450	454	508	520	514	504	476	567	599
pentane	-0.09	408	442	440	476	495	495	485	458	524	548
nitrobenzene	1.01	433	452	461	518	532	519	509	484	585	613
diethyl carbonate	0.45	402	444	447	495	510	500	493	466	542	575

^1^ Kamlet and Taft parameters; ^2^ Position of the ICT bands are given in nm.

**Table 4:** Summary of the optical properties of **PP11–PP20** in twenty-three solvents and Kamlet and Taft parameters π*.

Compounds	π*^1^	PP11^3^	PP12^3^	PP13^3^	PP14^3^	PP15^3^	PP16^3^	PP17^3^	PP18^3^	PP19^3^	PP20^3^
diethyl ether	0.27	384	404	424	462	480	474	467	442	502	531
toluene	0.54	388	408	429	474	486	485	476	451	520	552
chloroform	0.78	391	416	436	483	497	493	485	456	539	571
THF	0.58	387	411	428	476	490	479	472	448	518	553
1,4-dioxane	0.55	385	406	426	471	486	474	471	444	512	549
acetone	0.71	385	410	429	479	493	477	471	447	526	555
dichloromethane	0.82	388	417	433	482	497	489	482	452	537	567
DMSO	1.00	391	425	435	492	506	484	481	458	554	584
heptane	−0.08	379	400	422	457	473	476	468	439	478	523
acetonitrile	0.75	383	410	428	480	494	477	471	446	528	556
DMF	0.87	388	415	433	488	501	482	479	453	541	579
ethyl acetate	0.54	385	409	426	474	486	474	470	447	520	544
*p*-xylene	0.43	388	407	429	471	484	481	474	449	522	548
1,2-dichloroethane	0.81	388	421	432	481	496	487	480	453	536	567
dimethylacetamide	0.88	388	412	432	489	500	485	478	454	543	573
diglyme	0.64	386	412	429	479	490	480	472	450	524	559
cyclohexane	0.00	382	402	423	459	474	478	471	441	481	527
triethylamine	0.14	384	403	424	461	476	478	471	443	489	530
hexane	−0.08	378	400	422	455	471	474	467	437	476	521
anisole	0.73	389	415	432	481	495	487	482	454	530	570
pentane	−0.09	378	399	420	454	470	474	465	436	474	520
nitrobenzene	1.01	n.d.^2^	n.d. ^2^	437	491	504	493	482	459	552	582
diethyl carbonate	0.45	384	405	426	471	484	475	470	445	510	541

^1^ Kamlet and Taft parameters; ^2^ not determined. ^3^ Position of the ICT bands are given in nm.

**Table 5:** Fluorescence properties of the different compounds in dichloromethane and toluene solutions.

**Dichloromethane**										
**Compounds**	**PP1**	**PP2**	**PP3**	**PP4**	**PP5**	**PP6**	**PP7**	**PP8**	**PP9**	**PP10**
excitation (nm)	411	448	456	510	524	524	505	476	571	603
emission (nm)	-	512	499	-	558	617	-	-	654	-
Stokes shift (nm)	-	64	43	-	34	103	-	-	-	-
**Compounds**	**PP11**	**PP12**	**PP13**	**PP14**	**PP15**	**PP16**	**PP17**	**PP18**	**PP19**	**PP20**
excitation (nm)	388	417	433	482	497	489	482	452	537	567
emission (nm)	-	-	-	545	-	596	-	-	-	623
Stokes shift (nm)	-	-	-	63	-	107	-	-	-	56
**Toluene**										
**Compounds**	**PP1**	**PP2**	**PP3**	**PP4**	**PP5**	**PP6**	**PP7**	**PP8**	**PP9**	**PP10**
excitation (nm)	405	450	441	501	512	509	501	473	553	582
emission (nm)	-	487	495	546	546	574	-	-	613	626
Stokes shift (nm)	-	37	54	45	34	65	-	-	60	44
**Compounds**	**PP11**	**PP12**	**PP13**	**PP14**	**PP15**	**PP16**	**PP17**	**PP18**	**PP19**	**PP20**
excitation (nm)	388	408	429	474	486	485	476	451	520	552
emission (nm)	-	467	-	530	-	558	-	-	-	604
Stokes shift (nm)	-	59	-	56	-	73	-	-	-	52

**Table 6:** Electrochemical redox potentials of the studied compounds **PP1–PP20**.

Compounds	E_red_ (V/Fc)	E_Ox_ (V/Fc)	E_HOMO_ (eV)	E_LUMO_ (eV)	ΔE_el_ (eV)^1^	ΔE_opt_ (eV)^2^
**PP1**	−1.30	1.19	−5.99	−3.50	2.49	3.07
**PP2**	−1.26	1.18	−5.98	−3.54	2.44	2.82
**PP3**	−1.36	1.15	−5.95	−3.44	2.51	2.76
**PP4**	−1.33	0.68	−5.48	−3.47	2.01	2.45
**PP5**	−1.40	0.63	−5.43	−3.40	2.03	2.37
**PP6**	−1.32	0.79	−5.59	−3.48	2.11	2.46
**PP7**	−1.26	0.84	−5.64	−3.54	2.10	2.50
**PP8**	−1.38	0.95	−5.75	−3.42	2.33	2.63
**PP9**	−1.22	0.49	−5.29	−3.58	1.71	2.19
**PP10**	−1.30	0.41	−5.21	−3.50	1.71	2.08
**PP11**	−1.46	1.19	−5.99	−3.34	2.65	3.24
**PP12**	−1.44	1.18	−5.98	−3.36	2.62	3.02
**PP13**	−1.50	1.23	−6.03	−3.30	2.73	2.90
**PP14**	−1.56	0.65	−5.45	−3.24	2.21	2.58
**PP15**	−1.30	0.54	−5.34	−3.50	1.84	2.51
**PP16**	−1.44	0.77	−5.57	−3.36	2.21	2.60
**PP17**	−1.29	0.85	−5.65	−3.51	2.14	2.63
**PP18**	−1.52	0.94	−5.74	−3.28	2.46	2.78
**PP19**	−1.30	0.49	−5.29	−3.50	1.79	2.35
**PP20**	−1.30	0.36	−5.16	−3.50	1.66	2.23

^1^ All potentials are recorded in 0.1 M TBABF_4_/CH_3_CN, except for **PP15** and **PP20** for which electrochemistry was carried out in 0.1 M TBAClO_4_/CH_2_Cl_2_. E_HOMO_ (eV) = −4.8 − E_ox_ and **E_LUMO_** (eV) = −4.8 − E_red_; ^2^ Optical bandgaps determined in acetonitrile.

## Supplementary Materials

The following are available online at https://www.mdpi.com/1996-1944/12/8/1342/s1. ^1^H and ^13^C NMR spectra of all chromophores; Solvatochromism analyses in 23 solvents; contour plots of HOMO and LUMO energy levels of all dyes; cyclic voltammograms of all chromophores.
