# Supplementary material for: Push-Pull Chromophores Based on the Naphthalene Scaffold: Potential Candidates for Optoelectronic Applications
Source: Materials (Basel). 2019 Apr 24;12(8):1342. doi: 10.3390/ma12081342 (PMC6515425; doi:10.3390/ma12081342)
Supplement: Supplementary file 1 [file materials-12-01342-s001.pdf]

materials

MDPI

Supplementary Materials: Push-pull chromophores based on the naphthalene scaffold: Potential candidates for optoelectronic applications

Corentin Pigot [1,*], Guillaume Noirbent [1,*], Thanh-Tuân Bui [2], Sébastien Péralta [2], Didier Gigmes [1], Malek Nechab [1] and Frédéric Dumur [1,*]

1. H and ^{13}C NMR Spectra of All Chromophores

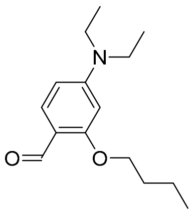

Figure S1. Chemical structure of 2-butoxy-4-diethylaminobenzaldehyde.

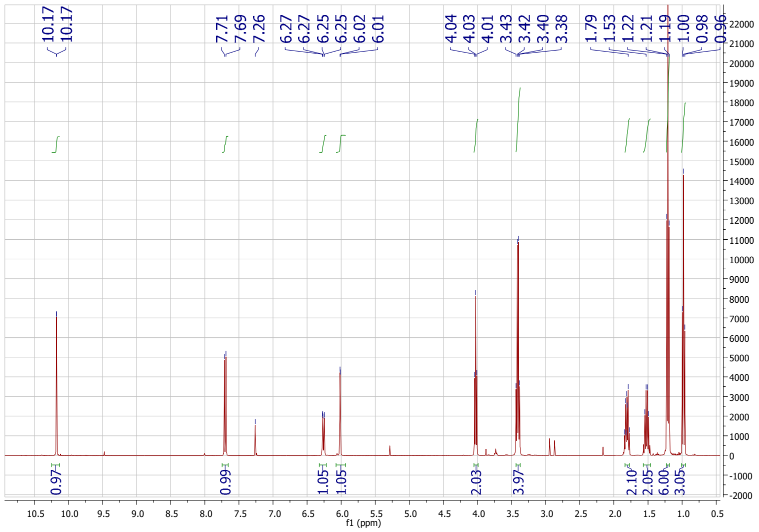

Figure S2. ^{1}H NMR spectrum of 2-butoxy-4-diethylaminobenzaldehyde in $CDCl_3$.

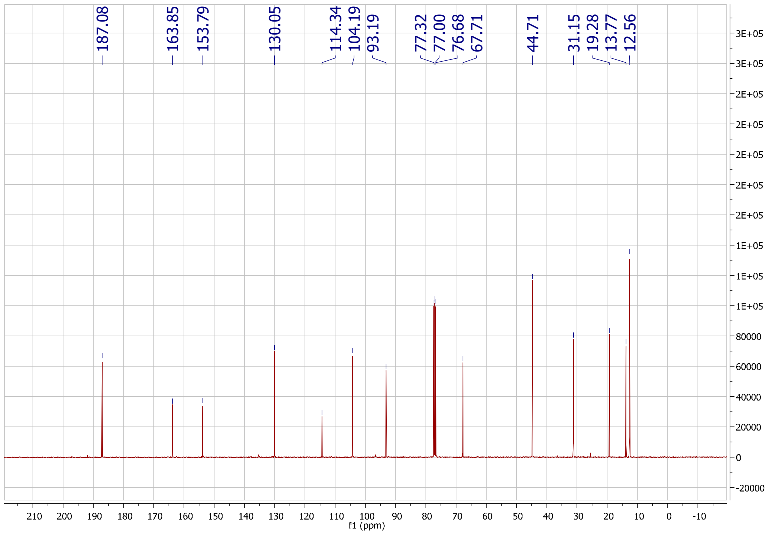

Figure S3. ^{13}C NMR spectrum of 2-butoxy-4-diethylaminobenzaldehyde in $CDCl_3$.

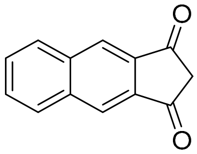

Figure S4. Chemical structure of **EA4.**

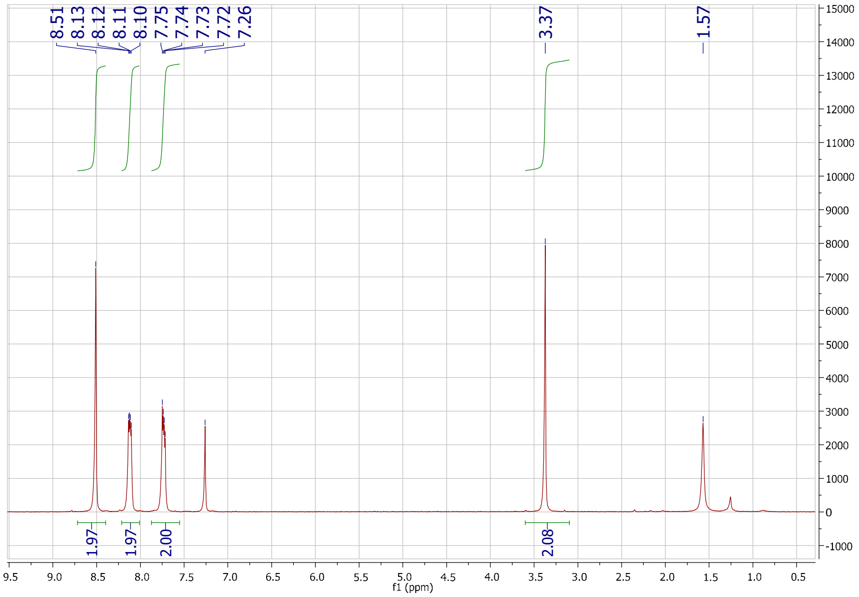

Figure S5. ^{1}H NMR spectrum of **EA4** in $CDCl_3$.

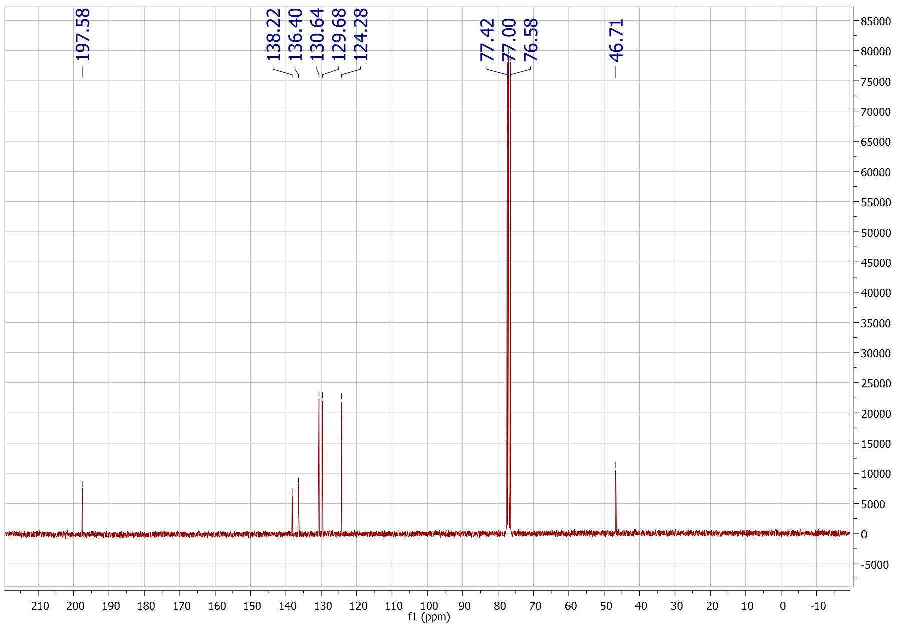

Figure S6. ^{13}C NMR spectrum of **EA4** in $CDCl_3$.

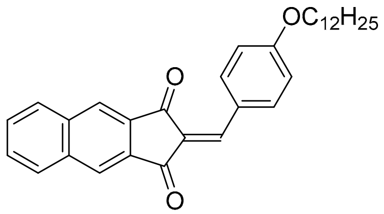

Figure S7. Chemical structure of PP1.

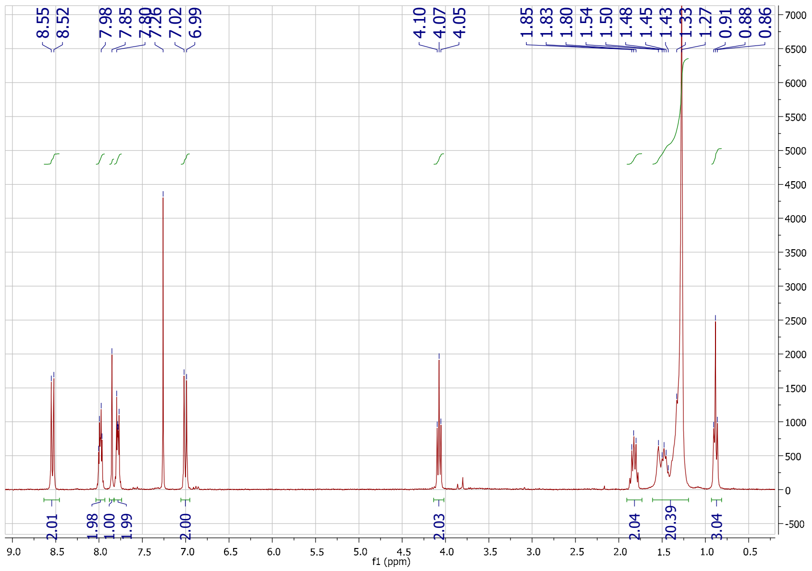

Figure S8. ^{1}H NMR spectrum of PP1 in $CDCl_3$.

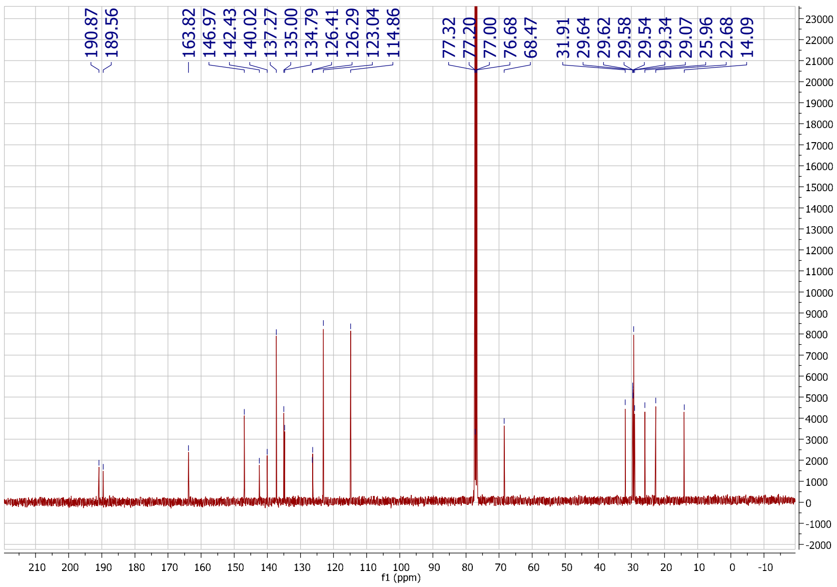

Figure S9. ^{13}C NMR spectrum of PP1 in $CDCl_3$.

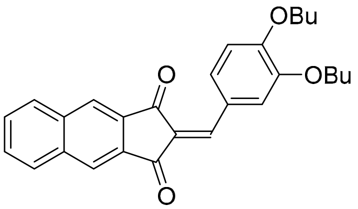

Figure S10. Chemical structure of PP2.

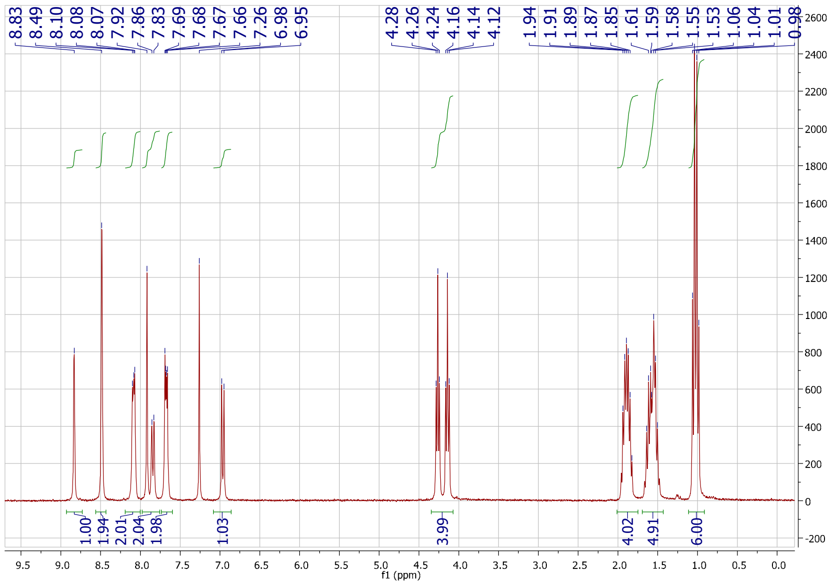

Figure S11. ^{1}H NMR spectrum of PP2 in $CDCl_3$.

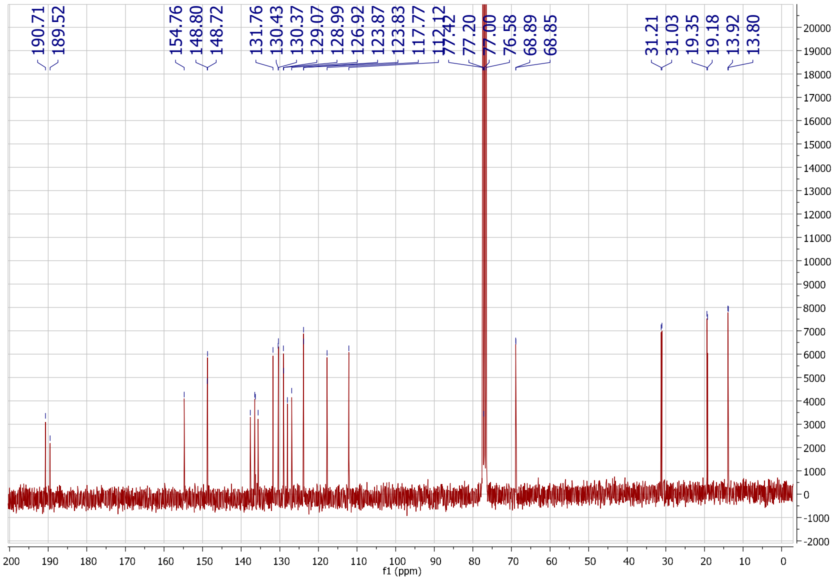

Figure S12. ^{13}C NMR spectrum of PP2 in $CDCl_3$.

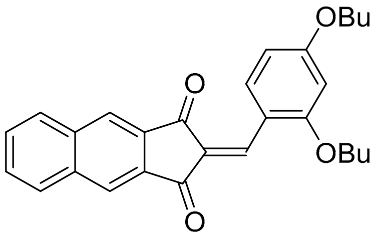

Figure S13. Chemical structure of PP3.

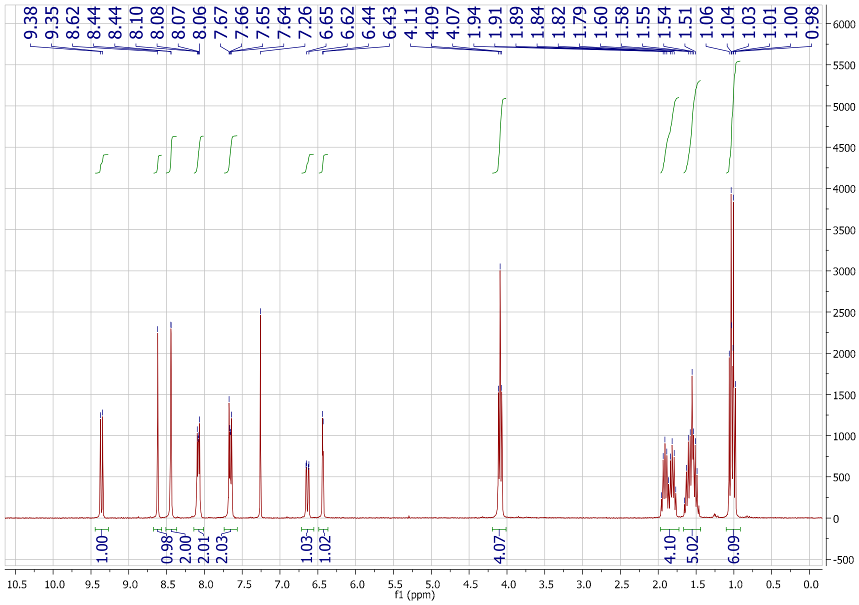

Figure S14. ^{1}H NMR spectrum of PP3 in $CDCl_3$.

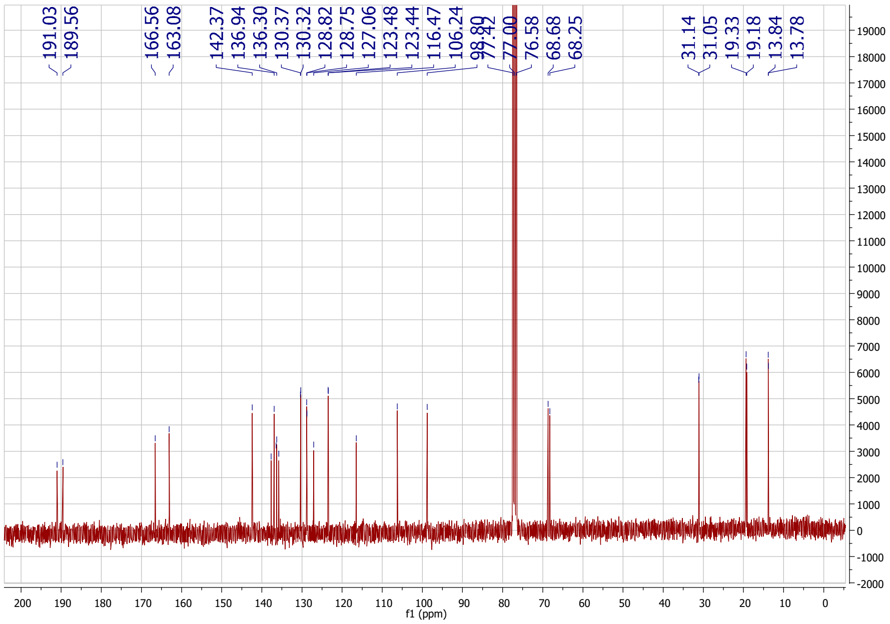

Figure S15. ^{13}C NMR spectrum of PP3 in $CDCl_3$.

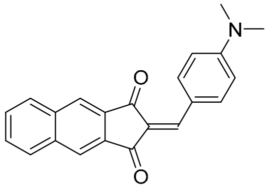

Figure S16. Chemical structure of PP4.

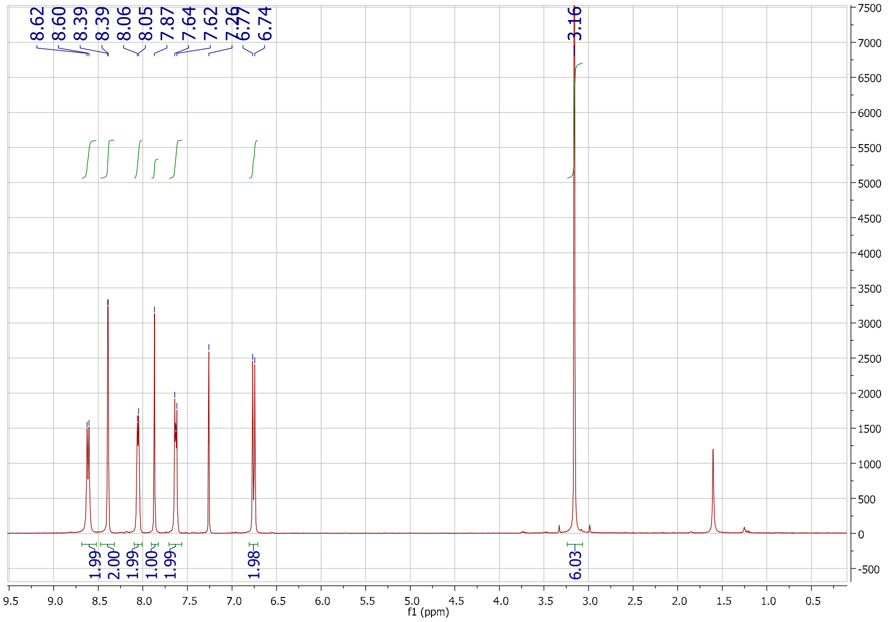

Figure S17. ^{1}H NMR spectrum of PP4 in $CDCl_3$.

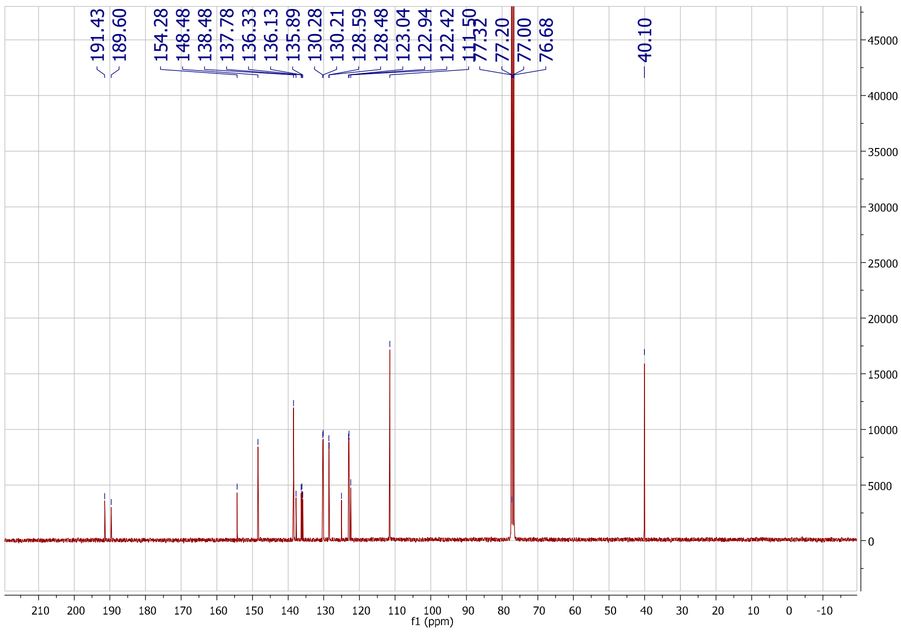

Figure S18. ^{13}C NMR spectrum of PP4 in $CDCl_3$.

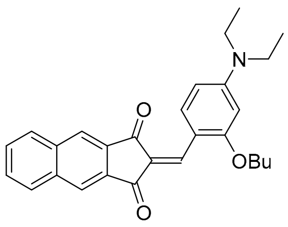

Figure S19. Chemical structure of PP5.

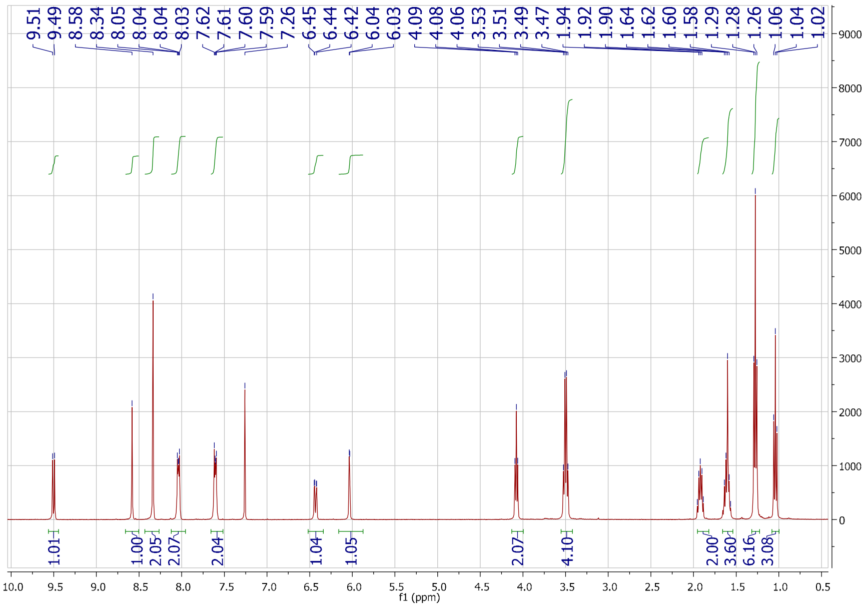

Figure S20. ^{1}H NMR spectrum of PP5 in $CDCl_3$.

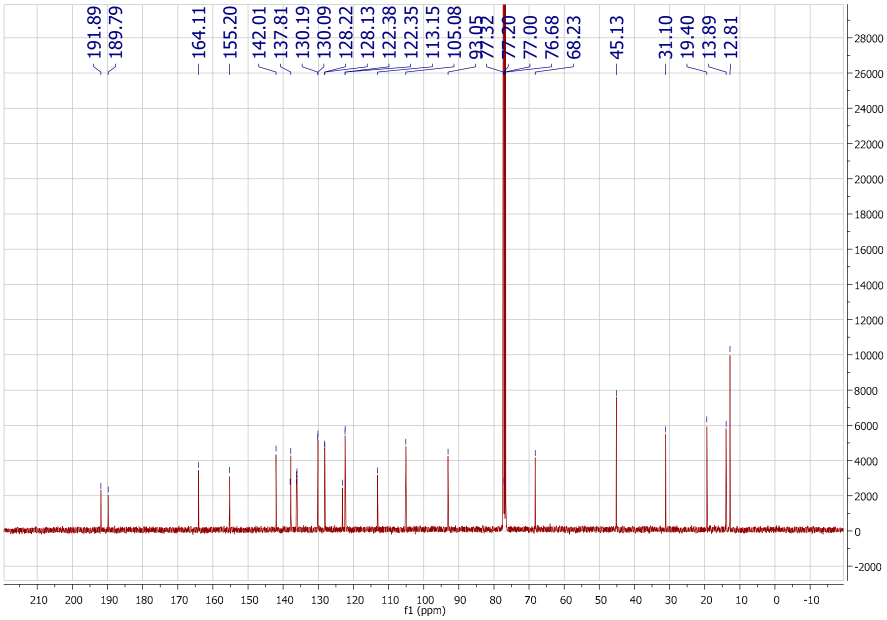

Figure S21. ^{13}C NMR spectrum of PP5 in $CDCl_3$.

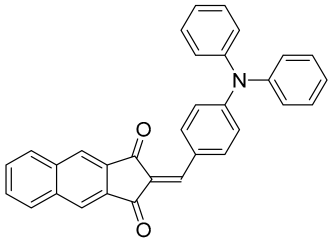

Figure S22. Chemical structure of PP6.

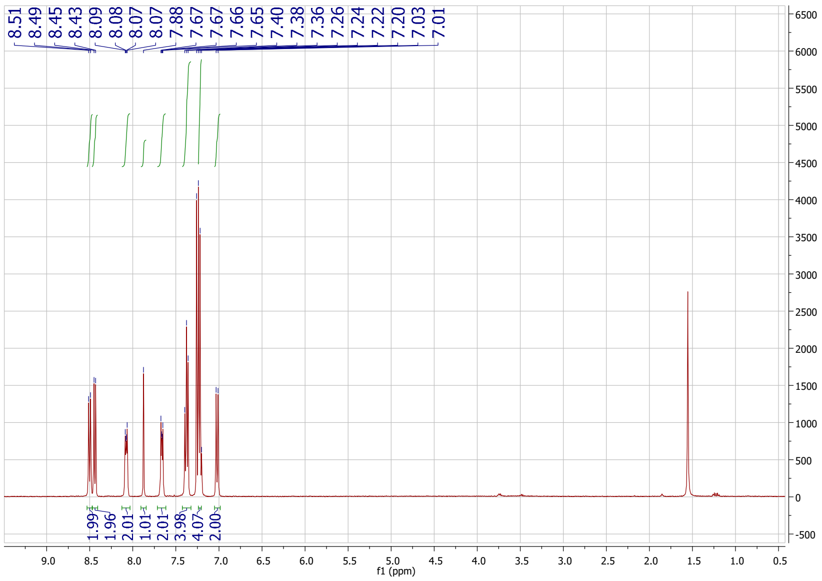

Figure S23. ^{1}H NMR spectrum of PP6 in $CDCl_3$.

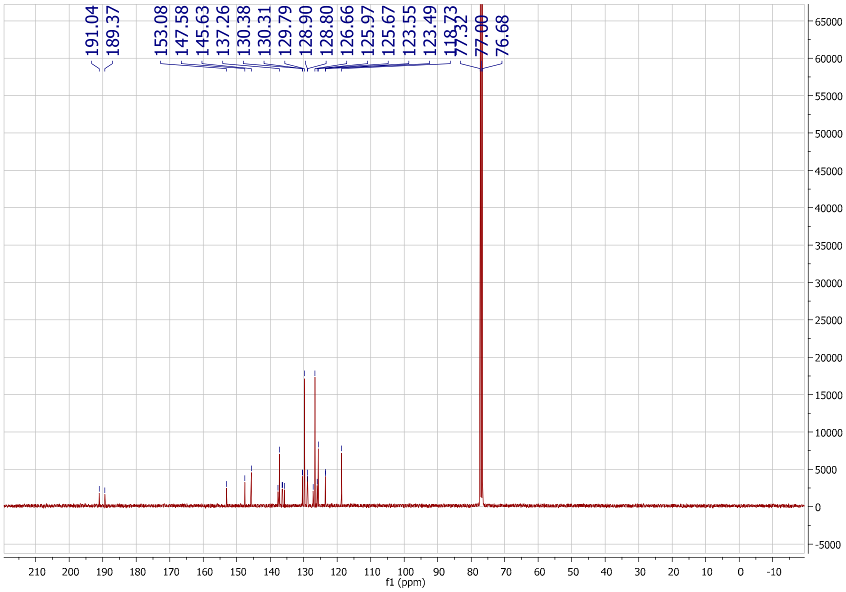

Figure S24. ^{13}C NMR spectrum of PP6 in $CDCl_3$.

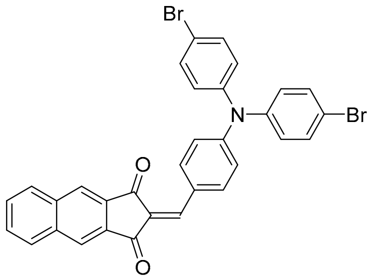

Figure S25. Chemical structure of PP7.

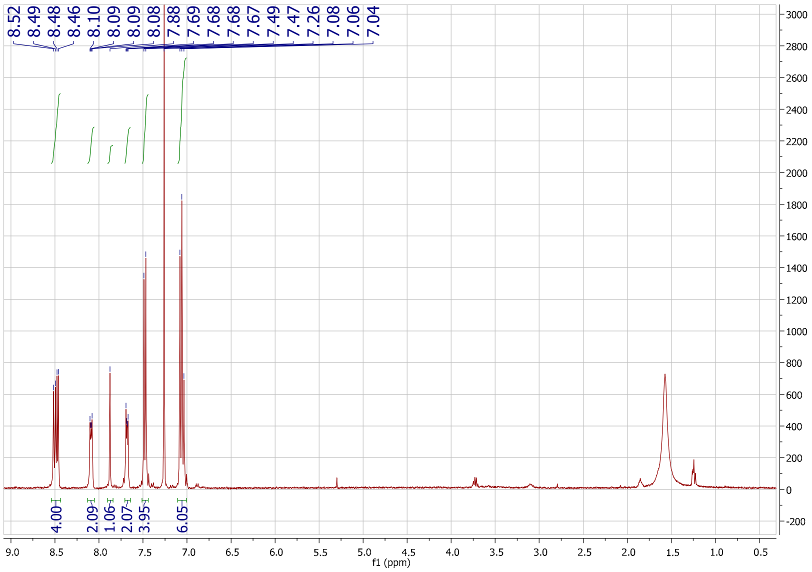

Figure S26. ^{1}H NMR spectrum of PP7 in $CDCl_3$.

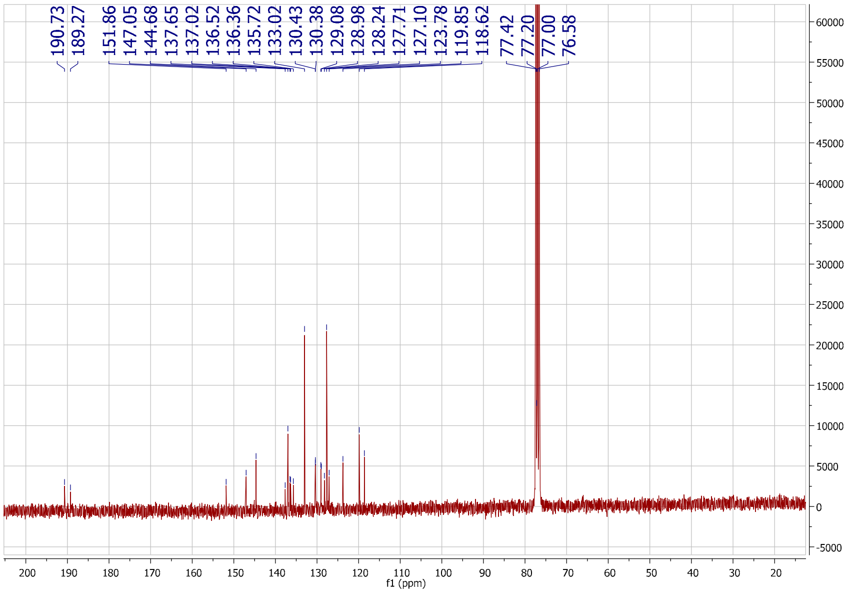

Figure S27. ^{13}C NMR spectrum of PP7 in $CDCl_3$.

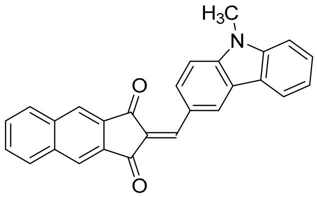

Figure S28. Chemical structure of PP8.

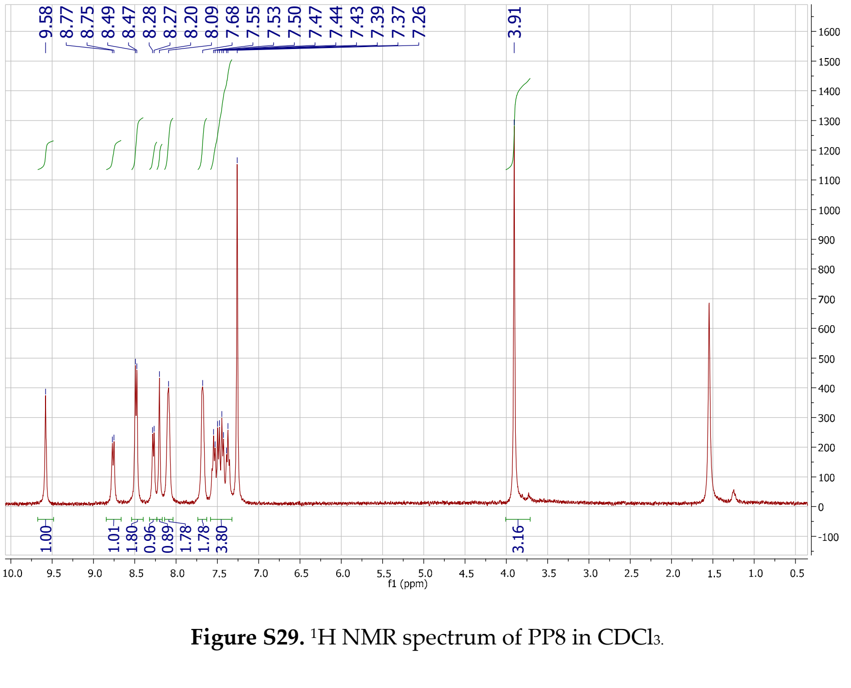

Figure S29. ^{1}H NMR spectrum of PP8 in $CDCl_3$.

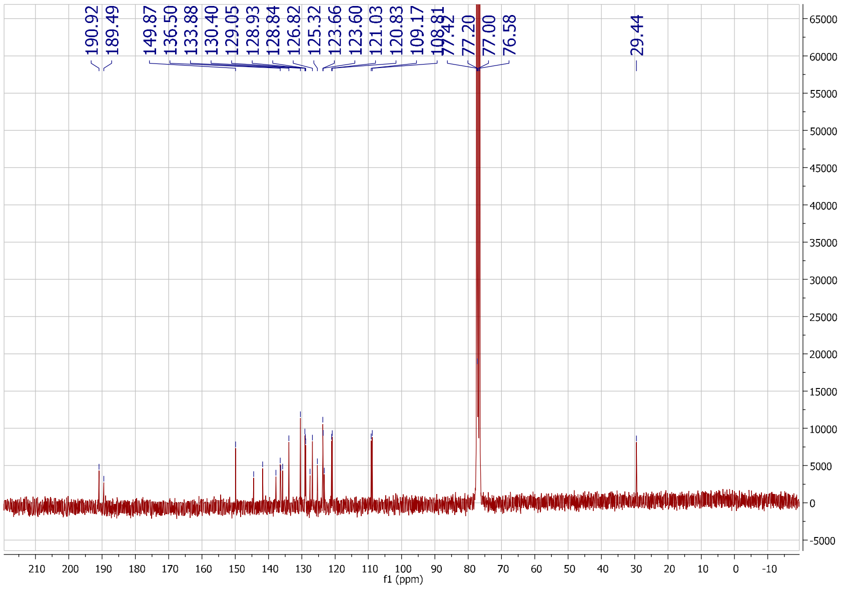

Figure S30. ^{13}C NMR spectrum of PP8 in $CDCl_3$.

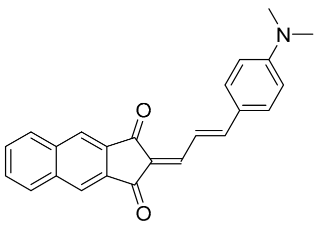

Figure S31. Chemical structure of PP9.

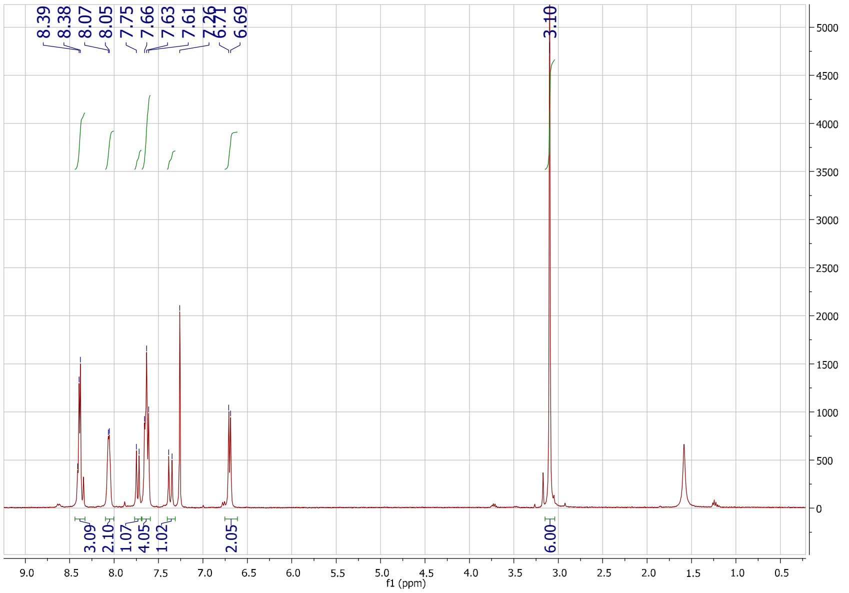

Figure S32. ^{1}H NMR spectrum of PP9 in $CDCl_3$.

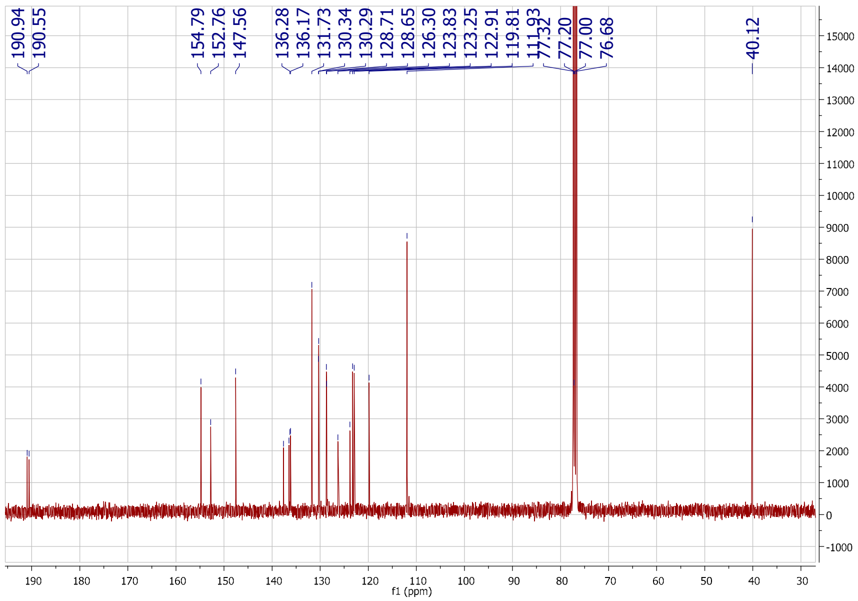

Figure S33. ^{13}C NMR spectrum of PP9 in $CDCl_3$.

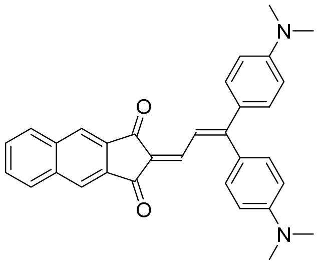

Figure S34. Chemical structure of PP10.

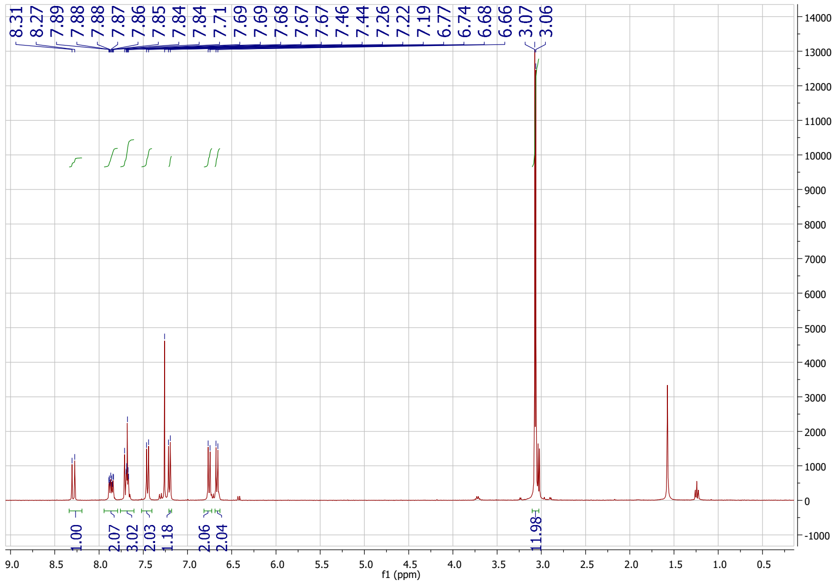

Figure S35. ^{1}H NMR spectrum of PP10 in $CDCl_3$.

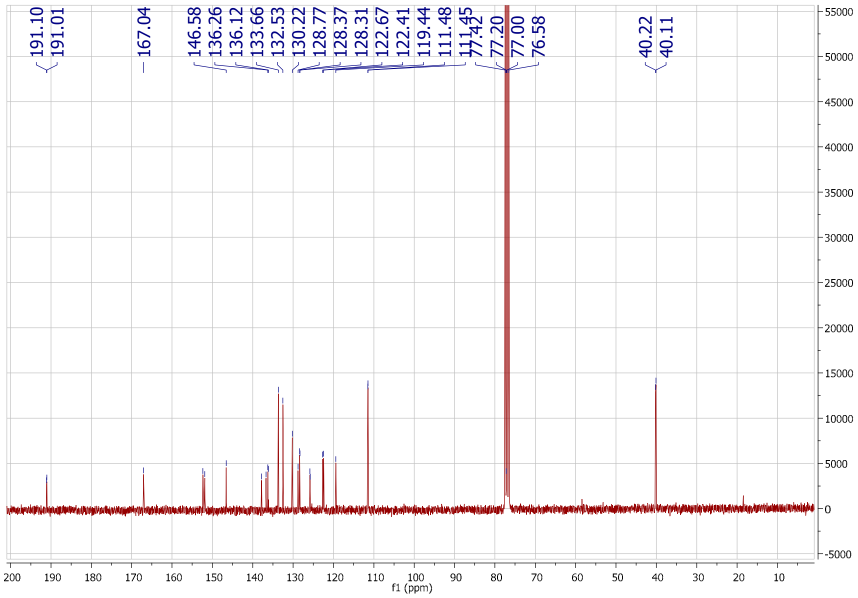

Figure S36. ^{13}C NMR spectrum of PP10 in $CDCl_3$.

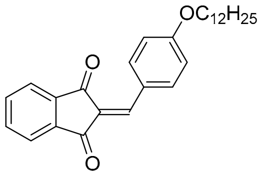

Figure S37. Chemical structure of PP11.

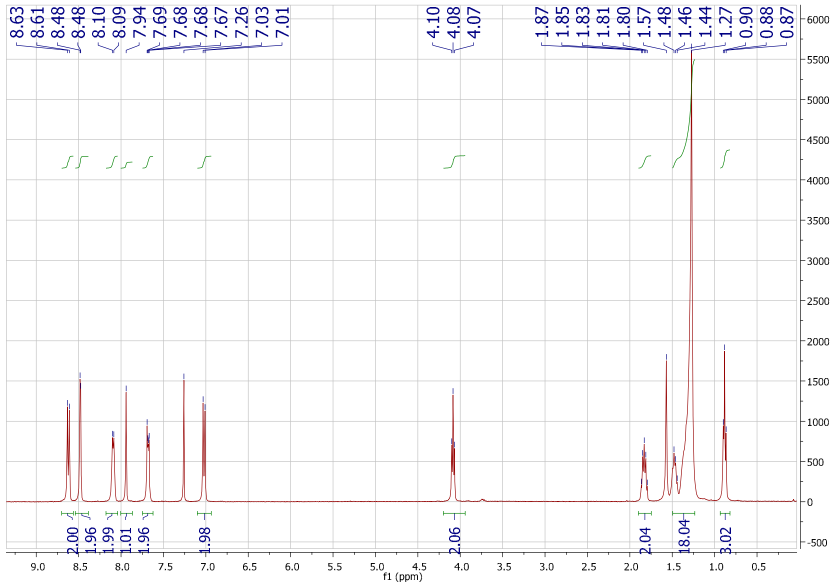

Figure S38. ^{1}H NMR spectrum of PP11 in $CDCl_3$.

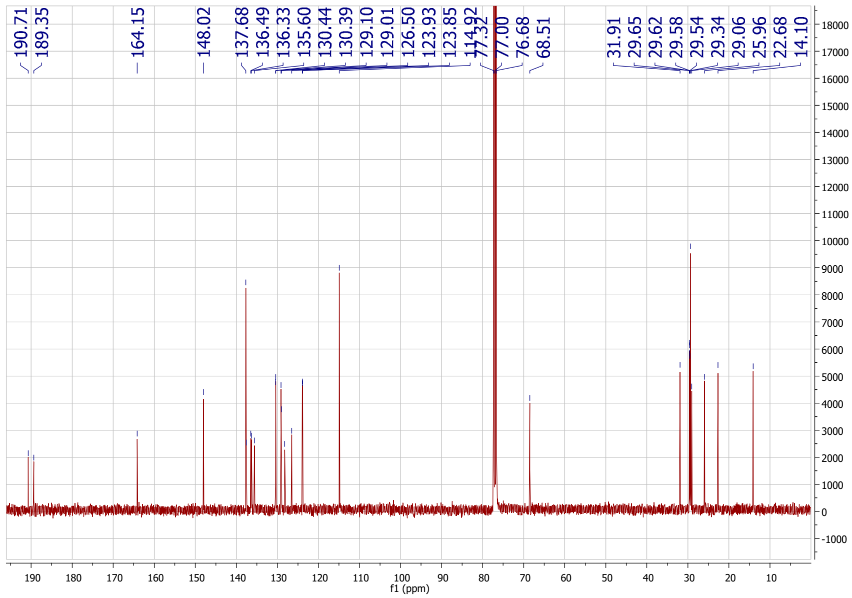

Figure S39. ^{13}C NMR spectrum of PP11 in $CDCl_3$.

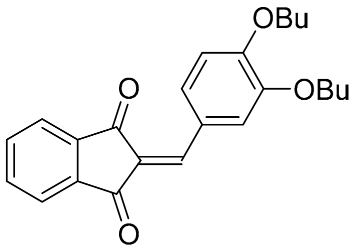

Figure S40. Chemical structure of PP12.

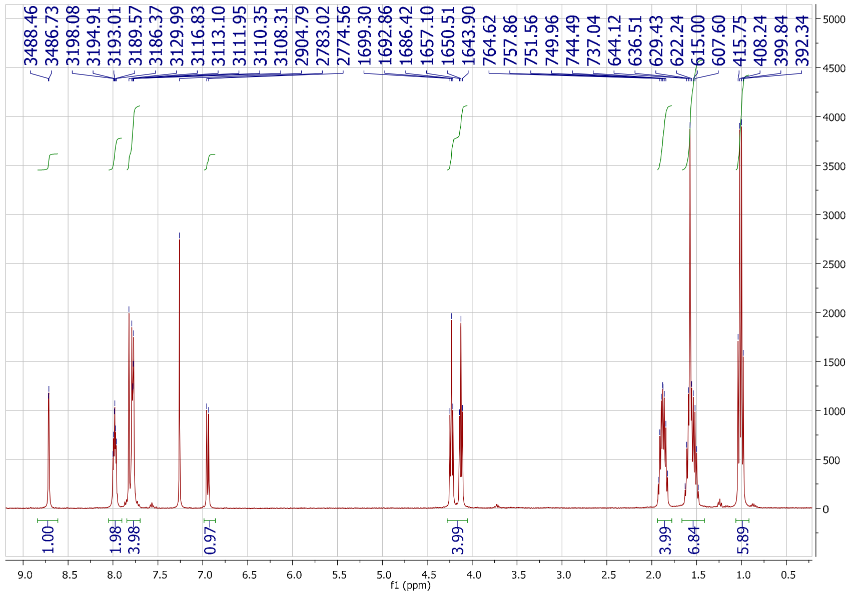

Figure S41. ^{1}H NMR spectrum of PP12 in $CDCl_3$.

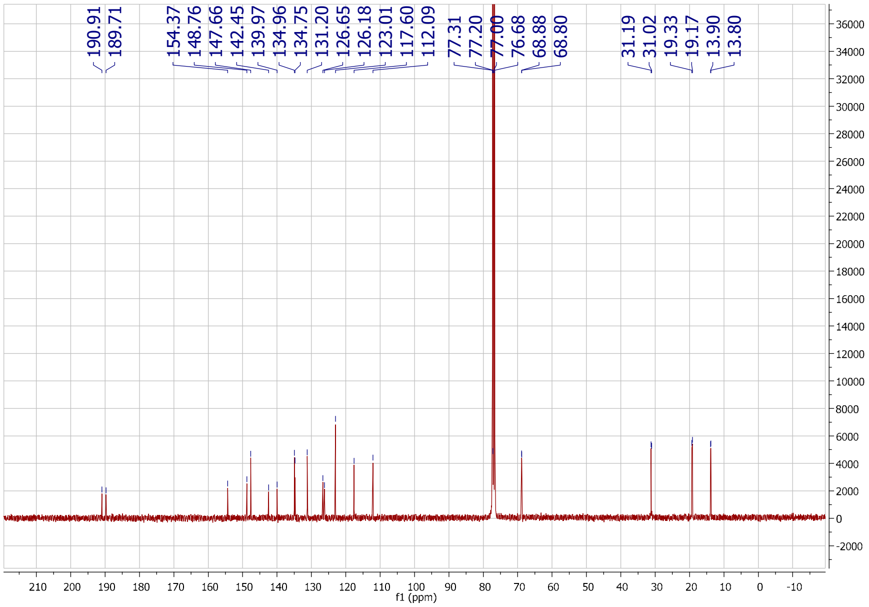

Figure S42. ^{13}C NMR spectrum of PP12 in $CDCl_3$.

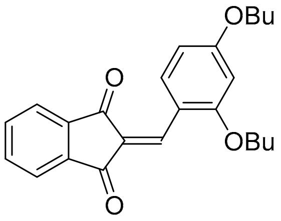

Figure S43. Chemical structure of PP13.

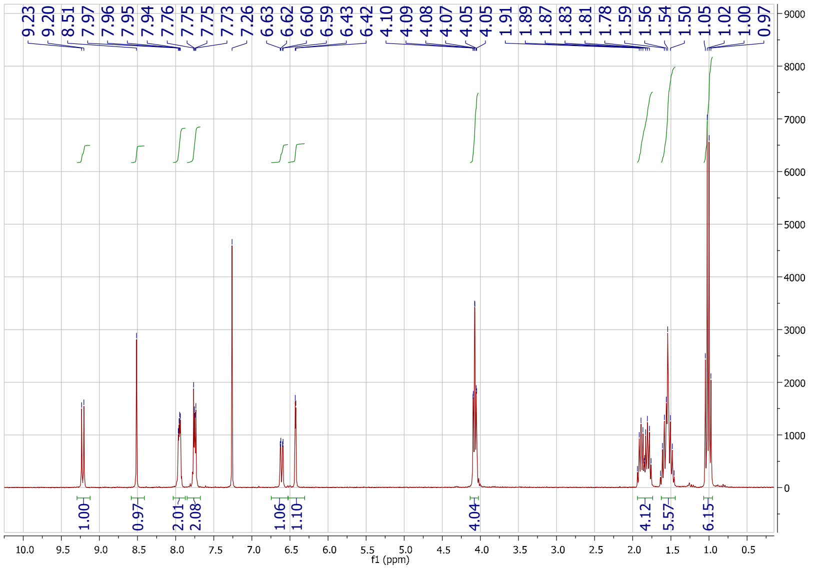

Figure S44. ^{1}H NMR spectrum of PP13 in $CDCl_3$.

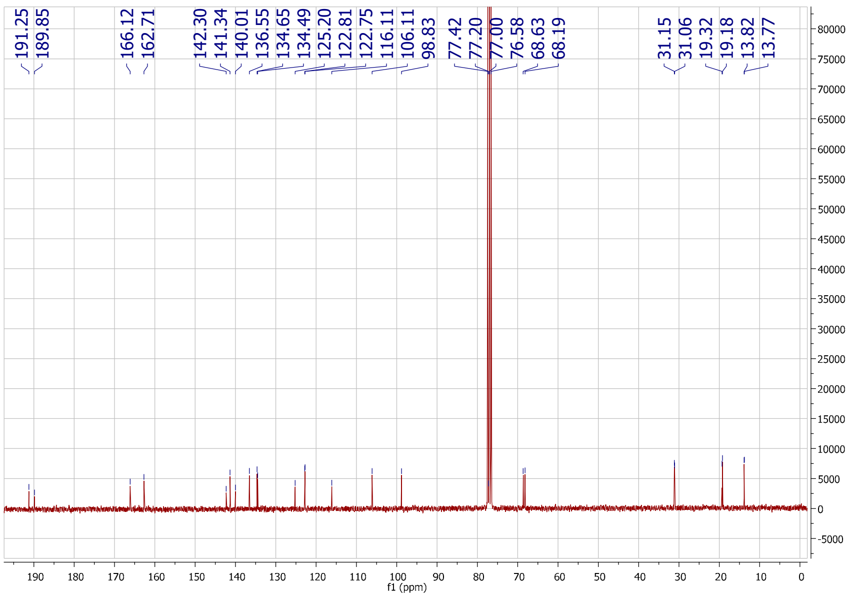

Figure S45. ^{13}C NMR spectrum of PP13 in $CDCl_3$.

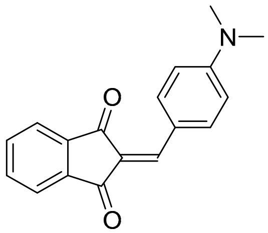

Figure S46. Chemical structure of PP14.

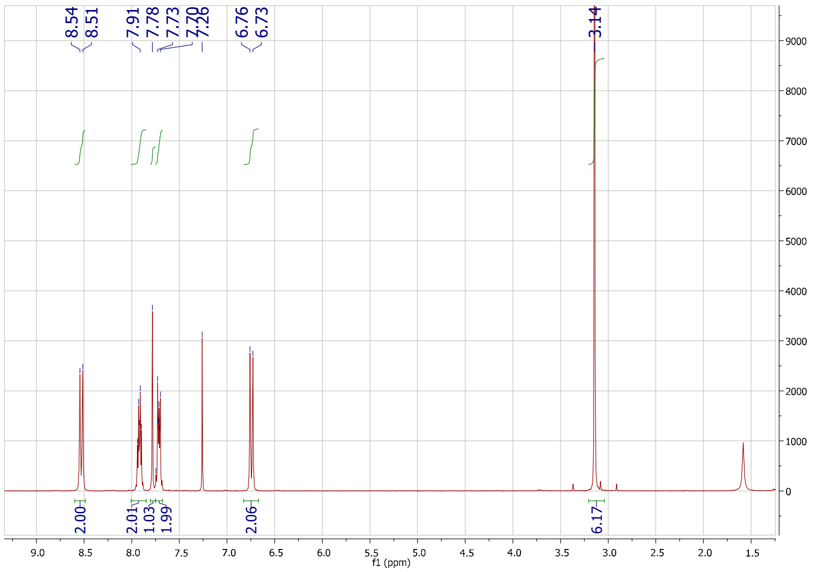

Figure S47. ^{1}H NMR spectrum of PP14 in $CDCl_3$.

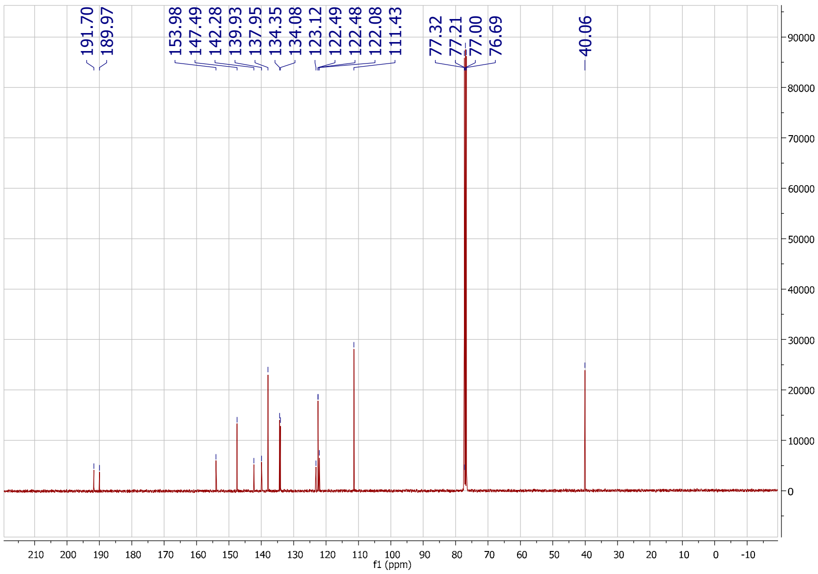

Figure S48. ^{13}C NMR spectrum of PP14 in $CDCl_3$.

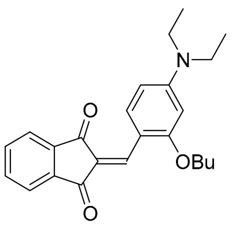

Figure S49. Chemical structure of PP15.

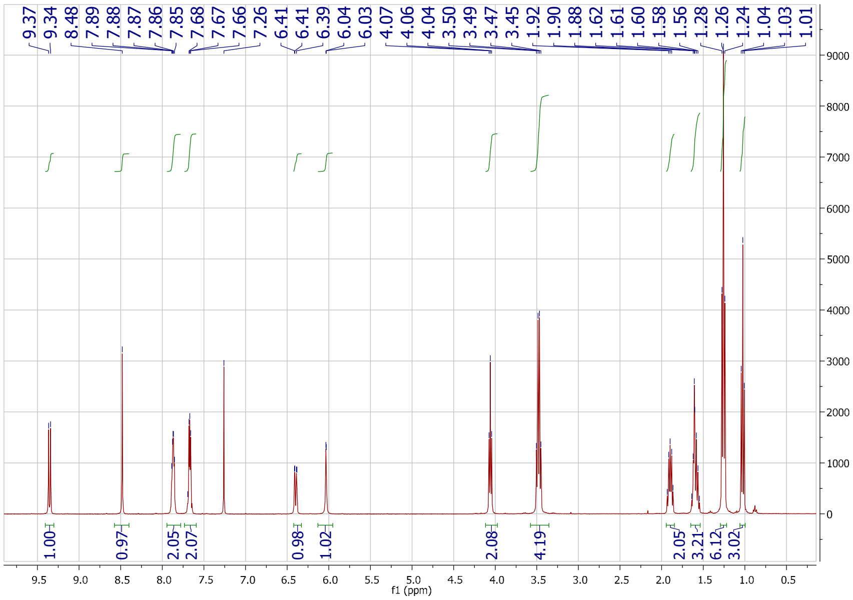

Figure S50. ^{1}H NMR spectrum of PP15 in $CDCl_3$.

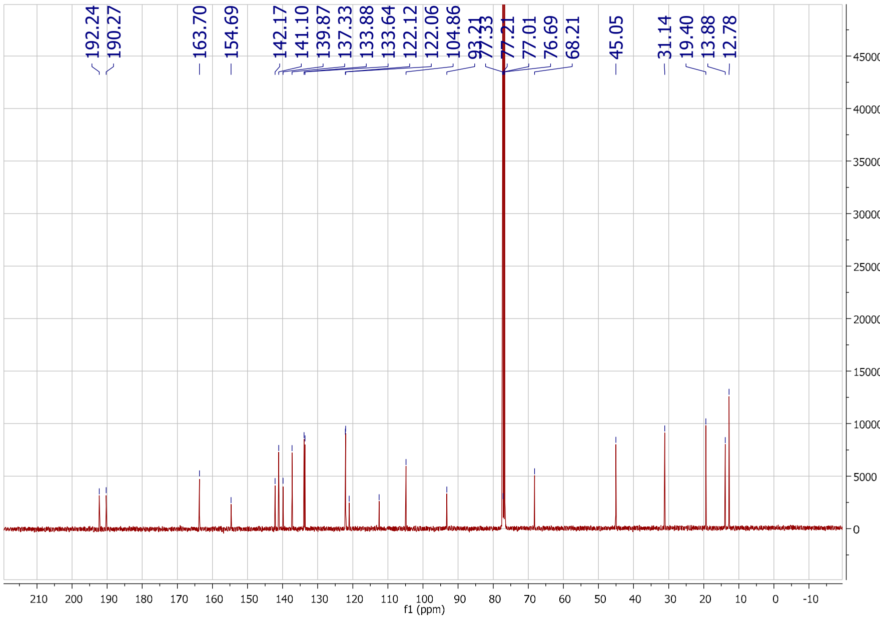

Figure S51. ^{13}C NMR spectrum of PP15 in $CDCl_3$.

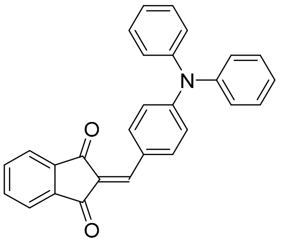

Figure S52. Chemical structure of PP16.

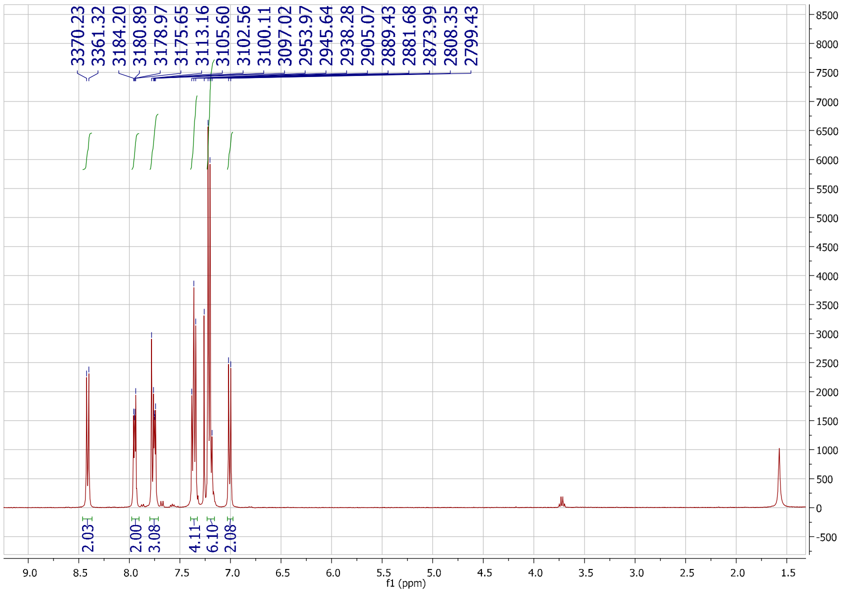

Figure S53. ^{1}H NMR spectrum of PP16 in $CDCl_3$.

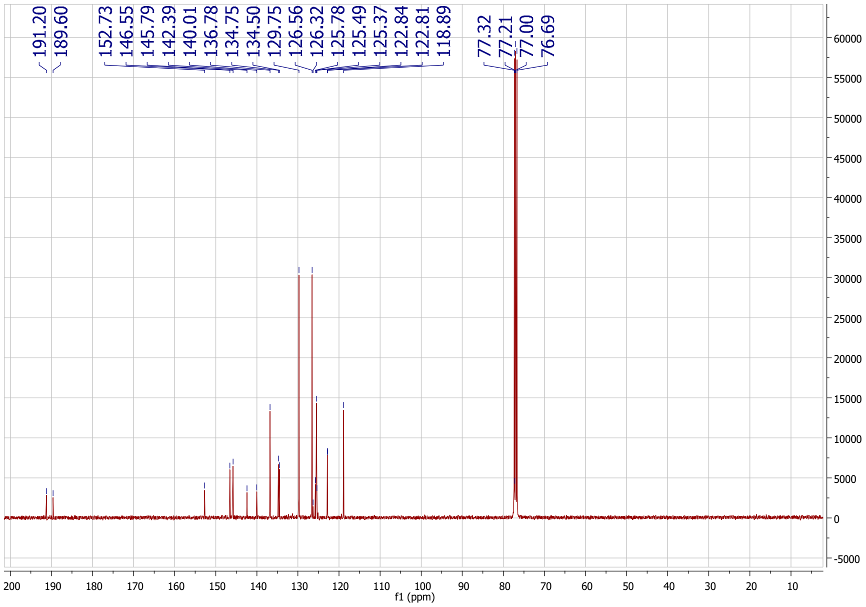

Figure S54. ^{13}C NMR spectrum of PP16 in $CDCl_3$.

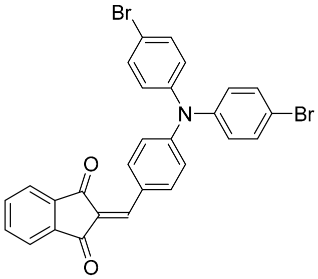

Figure S55. Chemical structure of PP17.

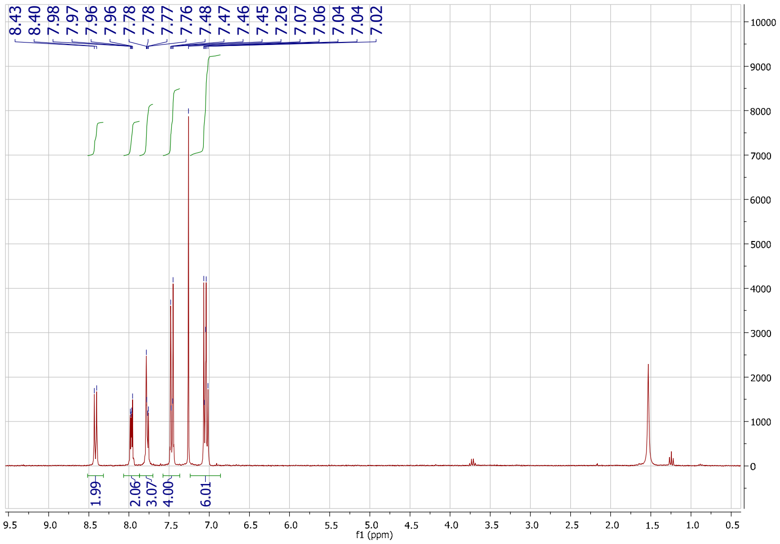

Figure S56. ^{1}H NMR spectrum of PP17 in $CDCl_3$.

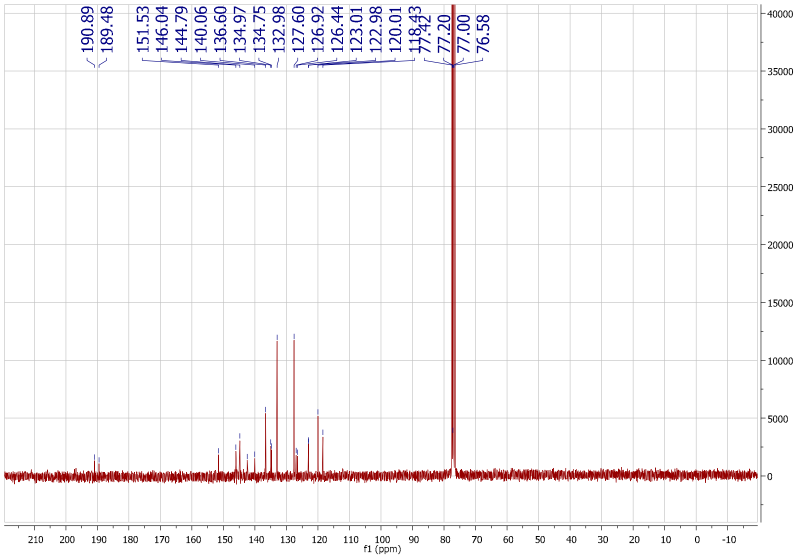

Figure S57. ^{13}C NMR spectrum of PP17 in $CDCl_3$.

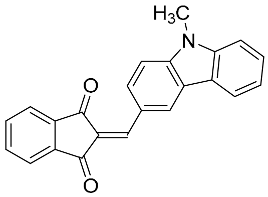

Figure S58. Chemical structure of PP18.

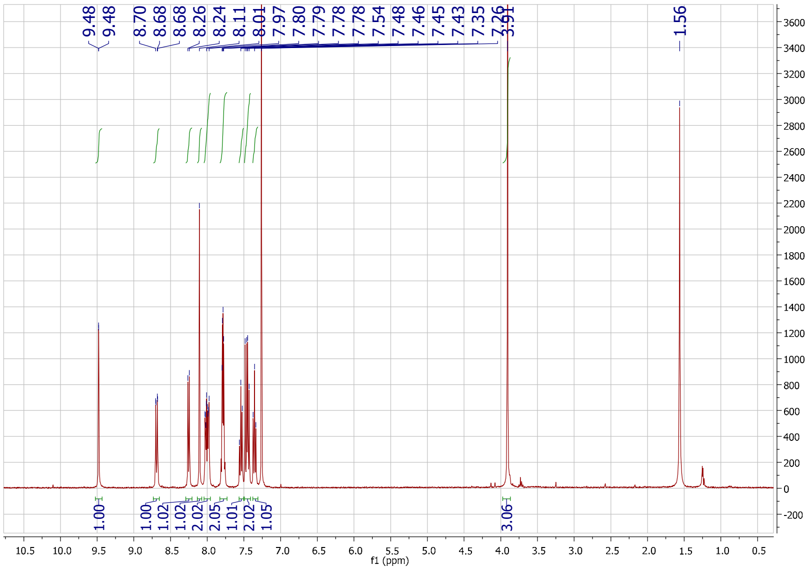

Figure S59. ^1H NMR spectrum of PP18 in $CDCl_3$.

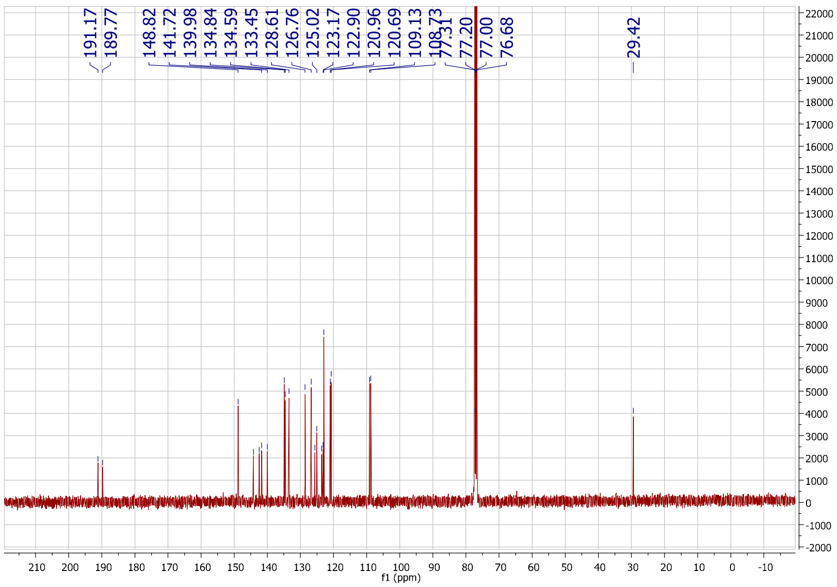

Figure S60. ^{13}C NMR spectrum of PP18 in $CDCl_3$.

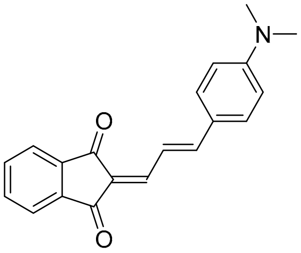

Figure S61. Chemical structure of PP19.

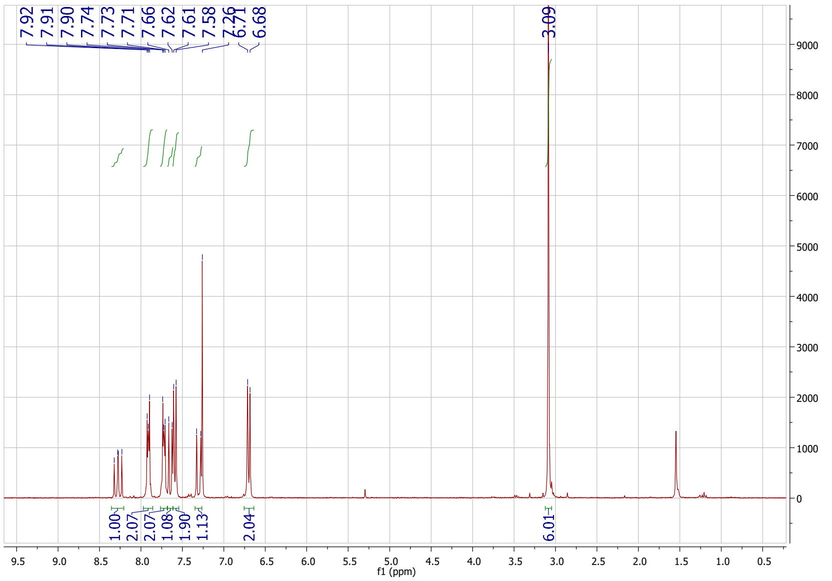

Figure S62. ^{1}H NMR spectrum of PP19 in $CDCl_3$.

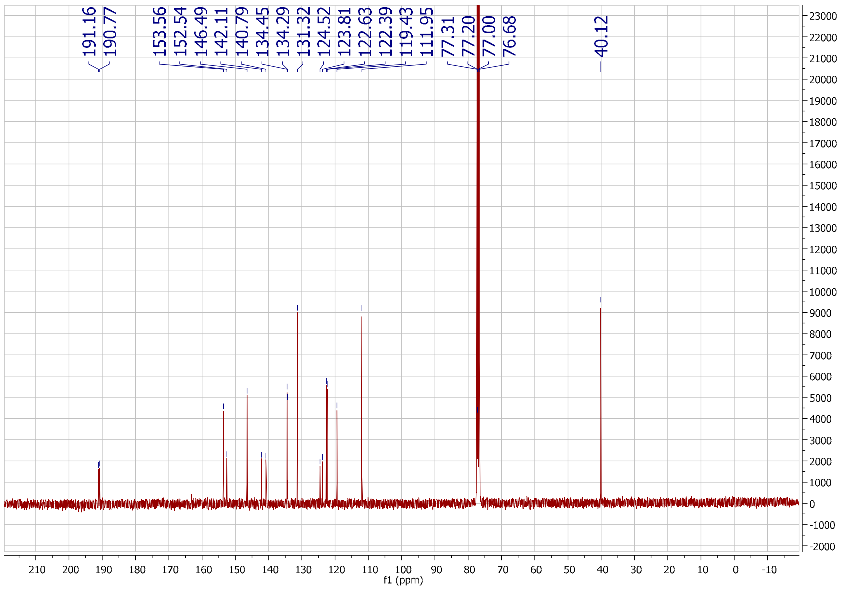

Figure S63. ^{13}C NMR spectrum of PP19 in $CDCl_3$.

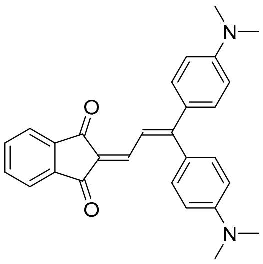

Figure S64. Chemical structure of PP20.

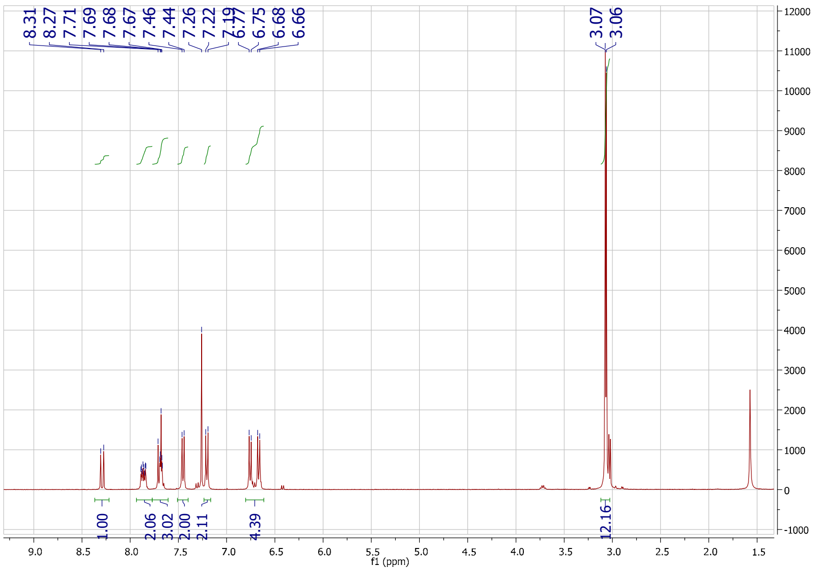

Figure S65. ^{1}H NMR spectrum of PP20 in $CDCl_3$.

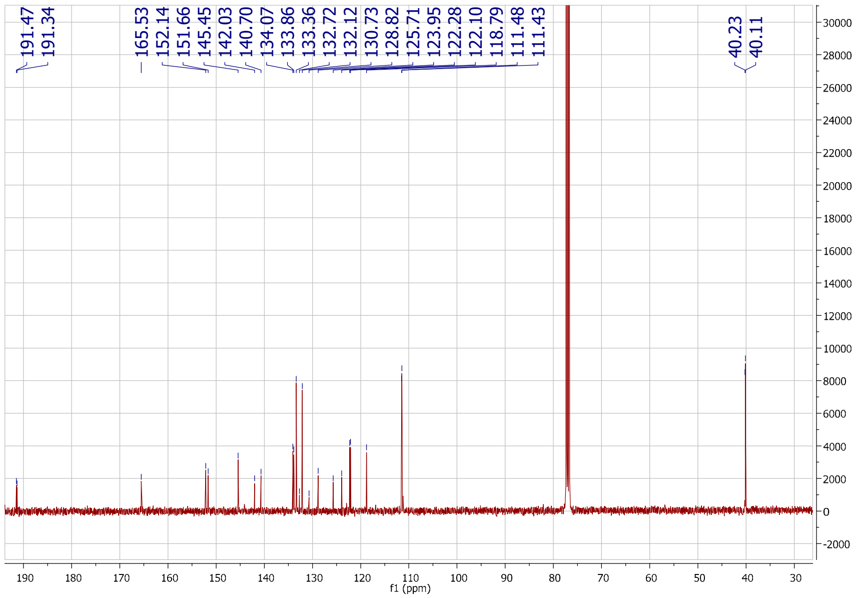

Figure S66. ^{13}C NMR spectrum of PP20 in $CDCl_3$.

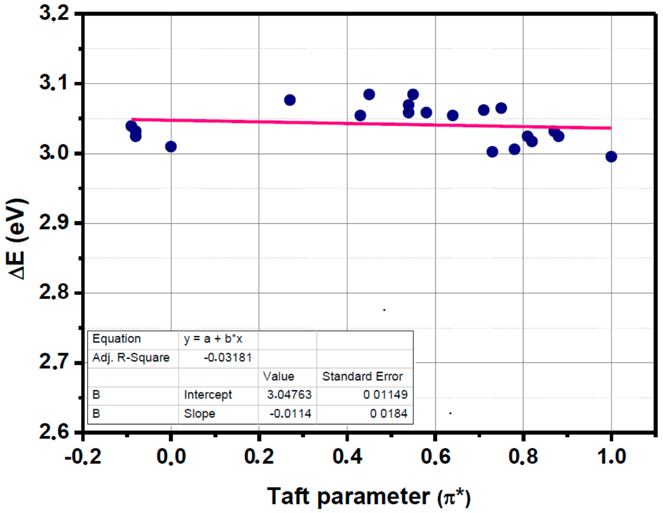

(a) Variation of the positions of the charge transfer band with Kamlet-Taft empirical parameters for **PP1**

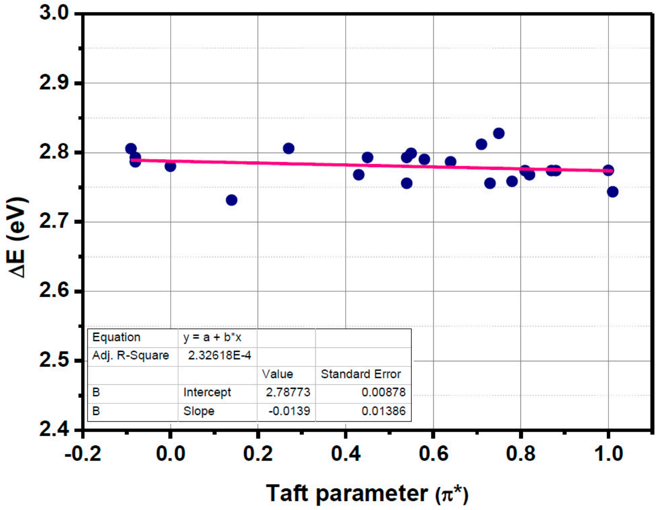

(**b**) Variation of the positions of the charge transfer band with Kamlet-Taft empirical parameters for **PP2**

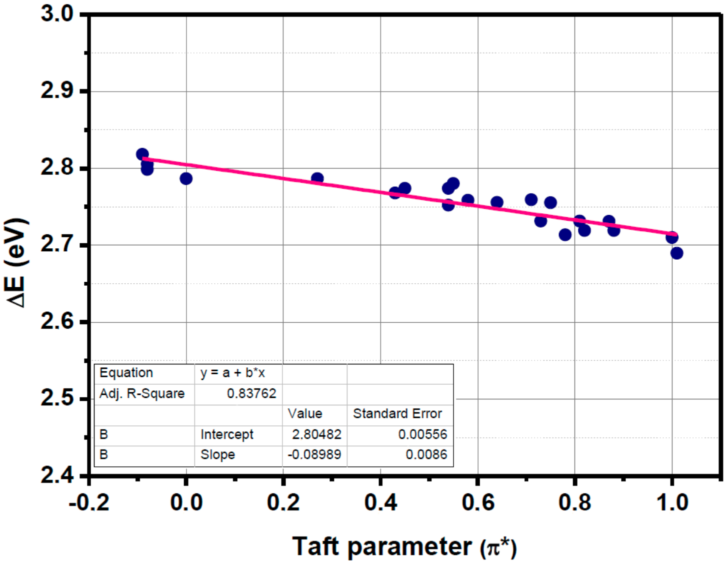

(**c**) Variation of the positions of the charge transfer band with Kamlet-Taft empirical parameters for **PP3**

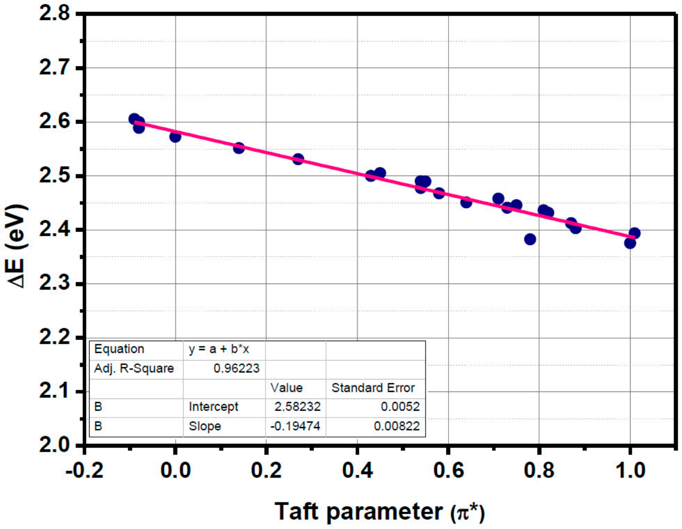

(**d**) Variation of the positions of the charge transfer band with Kamlet-Taft empirical parameters for **PP4**

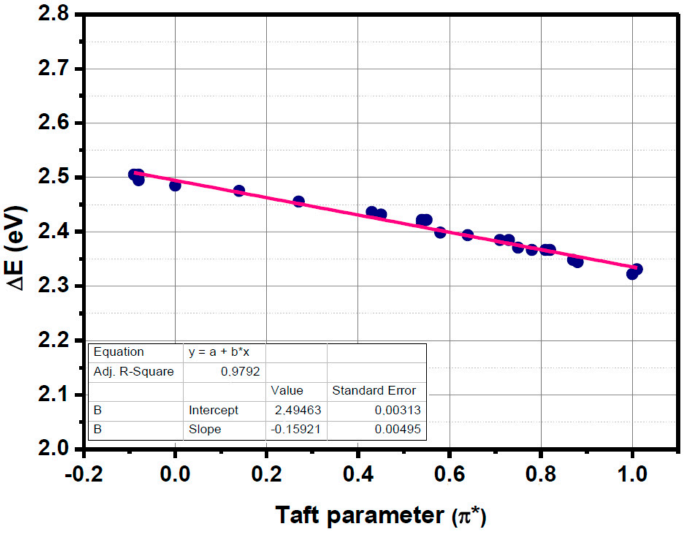

(**e**) Variation of the positions of the charge transfer band with Kamlet-Taft empirical parameters for **PP5**

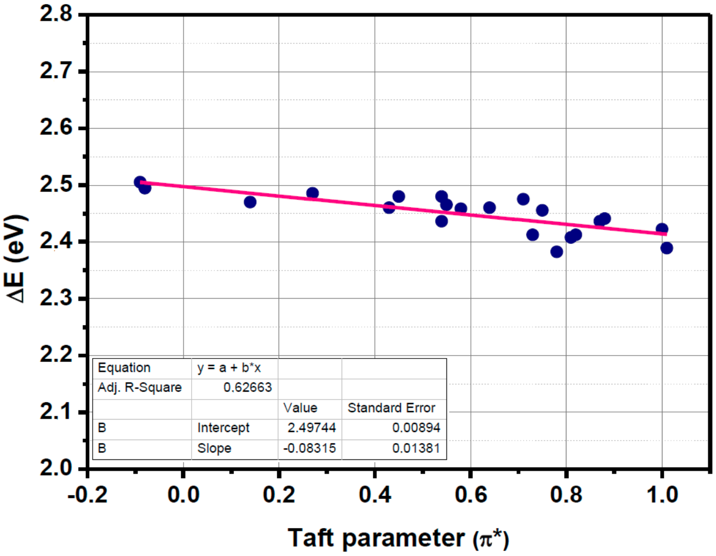

(**f**) Variation of the positions of the charge transfer band with Kamlet-Taft empirical parameters for **PP6**

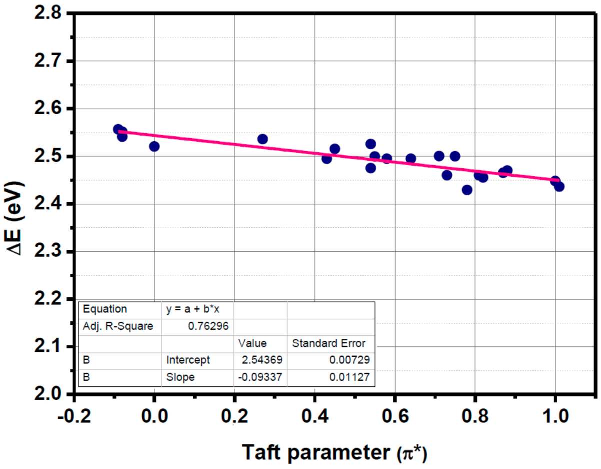

(**g**) Variation of the positions of the charge transfer band with Kamlet-Taft empirical parameters for **PP7**

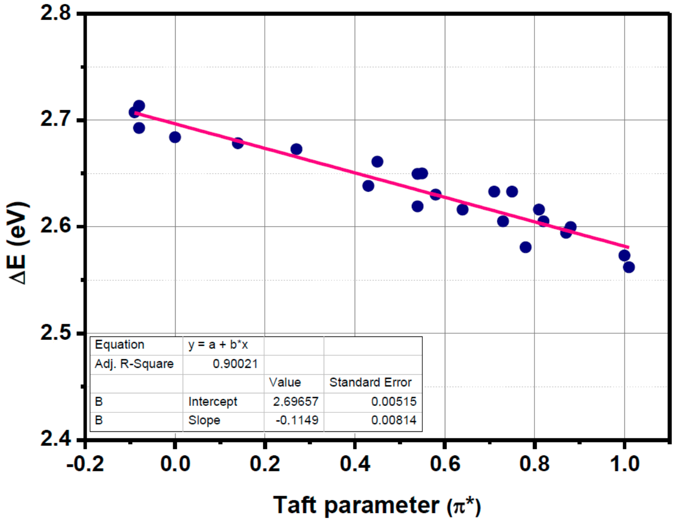

(**h**) Variation of the positions of the charge transfer band with Kamlet-Taft empirical parameters for **PP8**

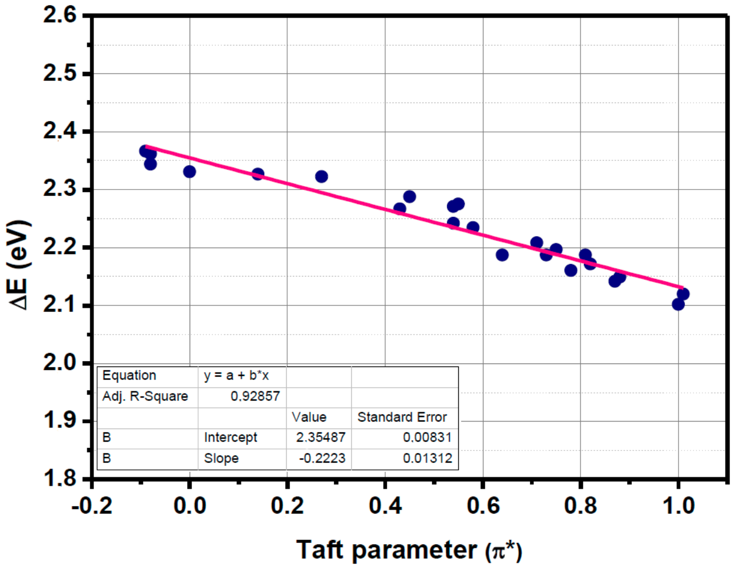

(**i**) Variation of the positions of the charge transfer band with Kamlet-Taft empirical parameters for **PP9**

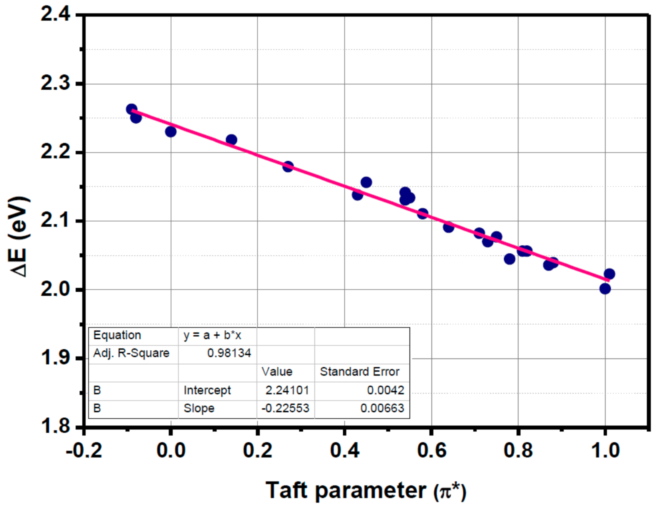

(**j**) Variation of the positions of the charge transfer band with Kamlet-Taft empirical parameters for **PP10**

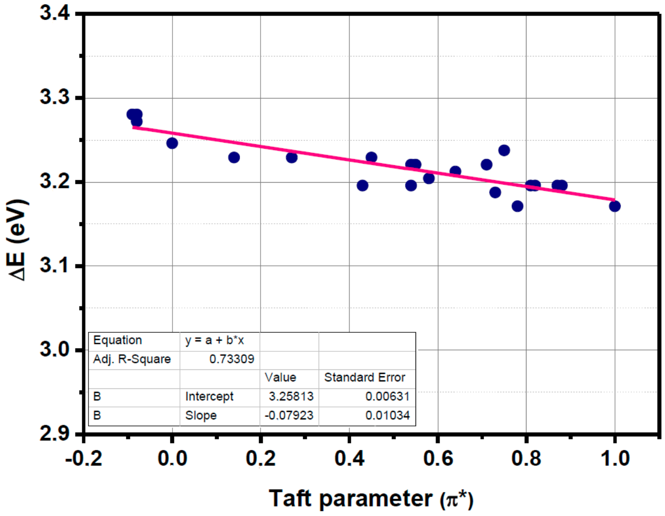

(**k**) Variation of the positions of the charge transfer band with Kamlet-Taft empirical parameters for **PP11**

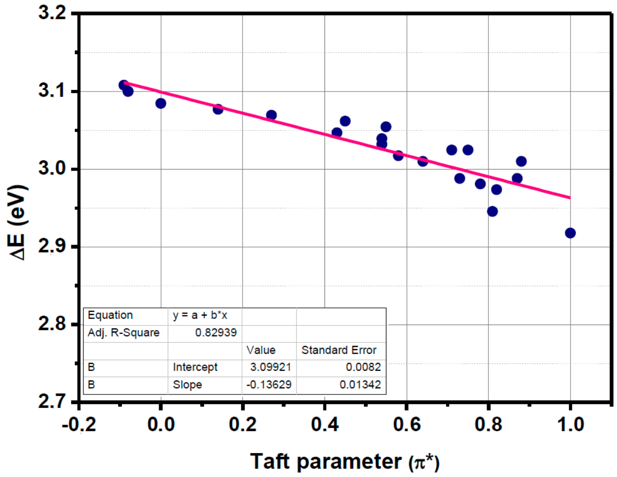

(**l**) Variation of the positions of the charge transfer band with Kamlet-Taft empirical parameters for **PP12**

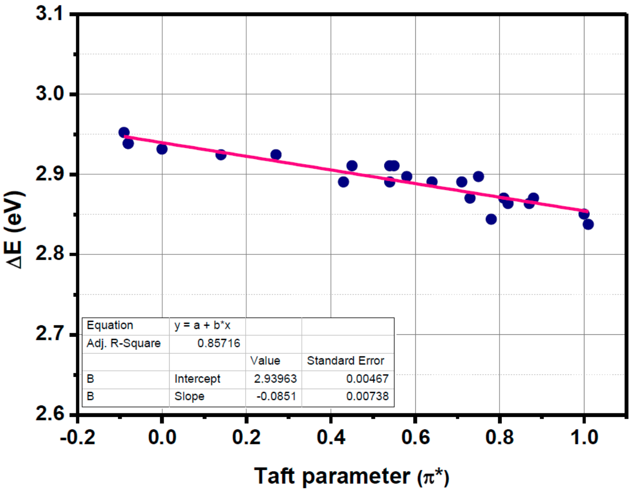

(**m**) Variation of the positions of the charge transfer band with Kamlet-Taft empirical parameters for **PP13**

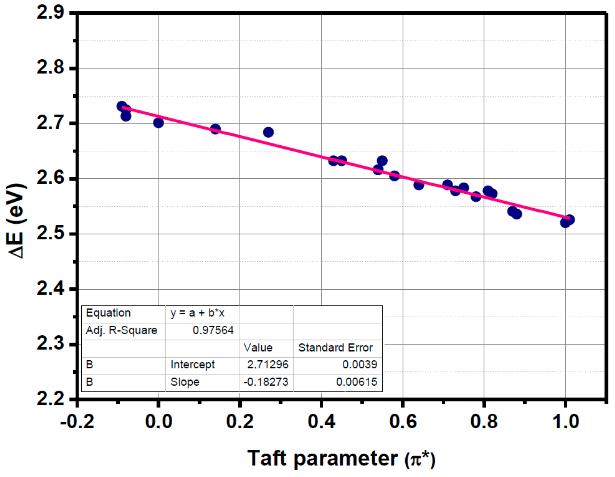

(**n**) Variation of the positions of the charge transfer band with Kamlet-Taft empirical parameters for **PP14**

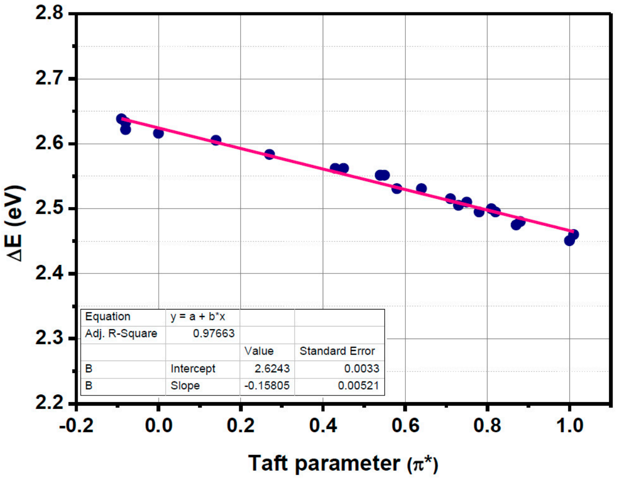

(**o**) Variation of the positions of the charge transfer band with Kamlet-Taft empirical parameters for **PP15**

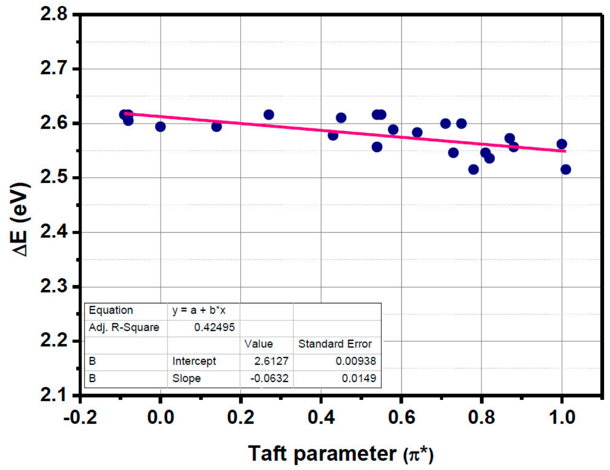

(**p**) Variation of the positions of the charge transfer band with Kamlet-Taft empirical parameters for **PP16**

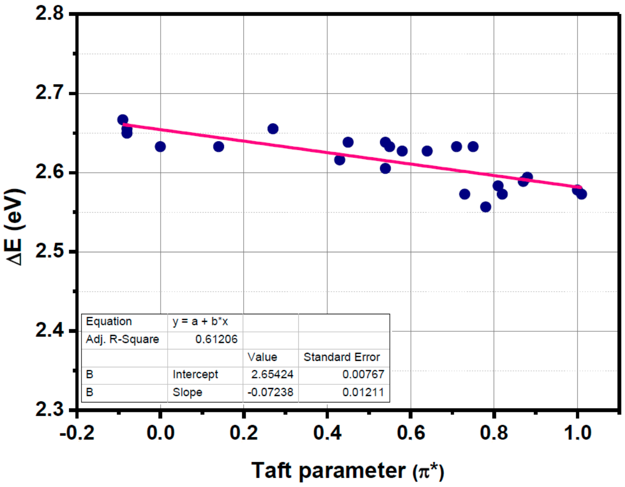

(**q**) Variation of the positions of the charge transfer band with Kamlet-Taft empirical parameters for **PP17**

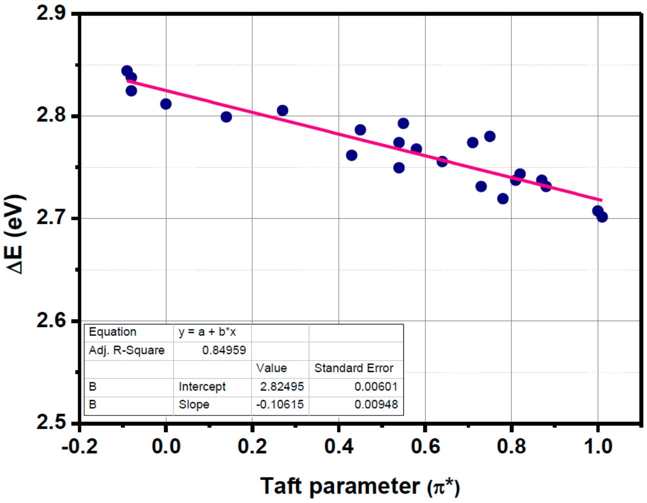

(**r**) Variation of the positions of the charge transfer band with Kamlet-Taft empirical parameters for **PP18**

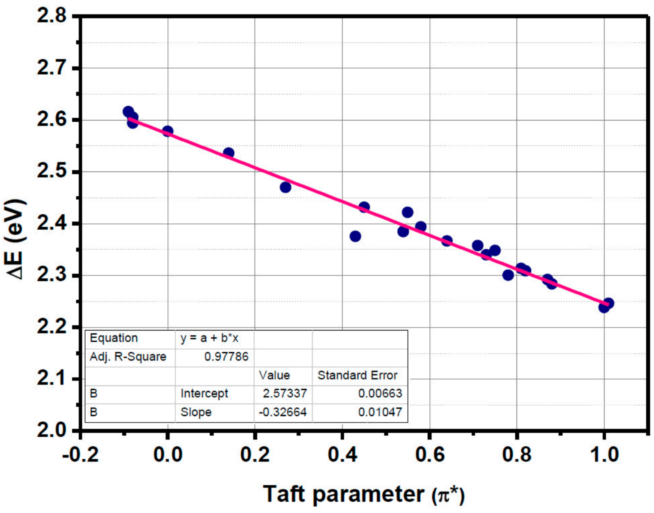

(**s**) Variation of the positions of the charge transfer band with Kamlet-Taft empirical parameters for **PP19**

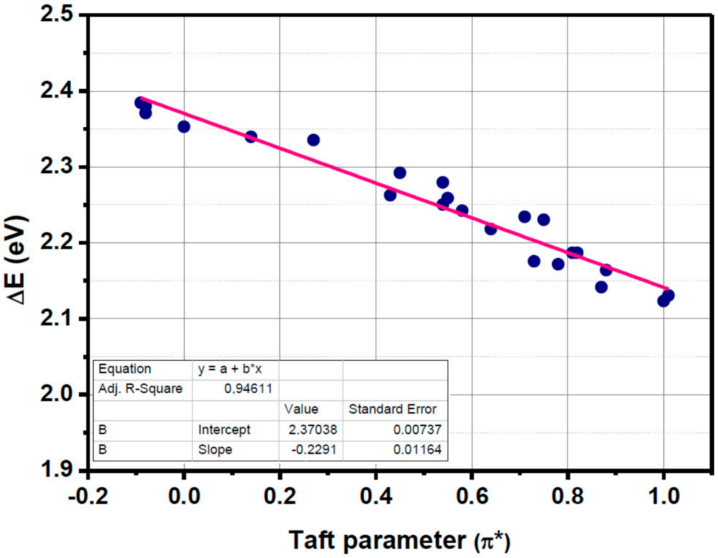

(**t**) Variation of the positions of the charge transfer band with Kamlet-Taft empirical parameters for **PP20**

Figure S67. Position of the absorption maxima of **PP1–PP20** in 23 solvents of different polarities vs. the Kamlet–Taft parameters π^*.

Results of the Multiple Linear Regression Analyses

The position of the UV/Vis absorption maxima with regard to the dipolarity/polarizability π^* and the hydrogen bonding capacity (α and β) of the solvent can be interpreted using the Kamlet–Taft equation:

$$\nu_{max}\,(cm^{-1}) = \nu_{max,0}\,(cm^{-1}) + a\alpha + b\beta + s\pi^*$$

Table S1. Solvent-independent correlation coefficients *a*, *b* and *s* of the Kamlet-Taft parameters α, β and π^* respectively, correlation coefficient (*R*), significance (*F*), standard deviation (*SD*), and number of solvents (*n*) calculated for the solvatochromism.

Compounds	$\nu_{max,0}$	*a*	*b*	*s*	*n*	*F*	R^2	*SD*
**PP1**	24489.934	357.133	−471.288	49.179	23	0.7598	0.25	540.712
**PP2**	22467.831	1108.869	93.375	−215.373	23	0.29054	0.18	183.052
**PP3**	22580.360	168.834	12.122	−699.601	23	1.58769×10^{-6}	0.80	132.105
**PP4**	20833.844	−425.609	−54.685	−1523.040	23	1.96732×10^{-13}	0.97	113.2703
**PP5**	20126.810	−158.867	−67.441	−1251.556	23	1.11022×10^{-15}	0.98	68.897
**PP6**	20052,604	520.692	351.585	−778.606	23	6.72195×10^{-5}	0.69	168.608
**PP7**	20434.795	−5.596	−48.236	−641.140	23	0.00102	0.58	214.138
**PP8**	21730.386	421.561	130.054	−994.633	23	9.46523×10^{-10}	0.91	110.523
**PP9**	18986.803	−20.373	66.150	−1805.218	23	6.07152×10^{-11}	0.92	180.033
**PP10**	18066.829	−164.093	51.189	−1824.052	23	3.33067×10^{-16}	0.98	91.669
**PP11**	26264.952	735.171	94.088	−730.096	23	9.23191×10^{-6}	0.77	133.062
**PP12**	24962.929	898.063	265.116	−1260.387	23	1.82274×10^{-7}	0.86	170.492
**PP13**	23681.457	270.105	180.216	−766.0370	23	1.13931×10^{-8}	0.88	96.726
**PP14**	21884.804	176.225	−34.957	−1470.310	23	3.77476×10^{-15}	0.97	84.769
**PP15**	21166.273	1.088	-29.560	−1267.154	23	3.10862×10^{-15}	0.98	72.688
**PP16**	21014.824	674.333	452.415	−707.986	23	9.48667×10^{-4}	0.59	186.545
**PP17**	23681.457	270.105	180.216	−766.036	23	1.13931×10^{-8}	0.87	96.726
**PP18**	22759.007	887.402	172.603	−969.369	23	1.22813×10^{-8}	0.88	121.412
**PP19**	20735.517	519.307	127.391	−2710.429	23	1.66533×10^{-15}	0.98	144.944
**PP20**	19094.207	887.853	170.044	−1958.662	23	2.49001×10^{-12}	0.95	154.825

Results of the Linear Correlation Analyses

The position of the UV/Vis absorption maxima with regard to the dipolarity/polarizability π^* can be interpreted using a simplified version of the Kamlet–Taft equation:

$$\nu_{max}\,(cm^{-1}) = \nu_{max,0}\,(cm^{-1}) + s\pi^*$$

Table S2. Solvent-independent correlation coefficient *s* of the Kamlet-Taft parameters π^* and number of solvents (*n*) calculated for the solvatochromism.

Compounds	$\nu_{max,0}$	*s*	*n*	R^2
**PP1**	3.04763	−0.0114	23	0.02
**PP2**	2.78773	−0.0139	23	0.04

**PP3**	2.80482	−0.08989	23	0.84
**PP4**	2.58232	−0.19474	23	0.96
**PP5**	2.49463	−0.15921	23	0.99
**PP6**	2.49744	−0.08315	23	0.64
**PP7**	2.54369	−0.09337	23	0.76
**PP8**	2.69657	−0.1149	23	0.90
**PP9**	2.35487	−0.2223	23	0.96
**PP10**	2.24101	−0.22553	23	0.98
**PP11**	3.25813	−0.07923	23	0.74
**PP12**	3.09921	−0.13629	23	0.84
**PP13**	2.93963	−0.0851	23	0.86
**PP14**	2.71296	−0.18273	23	0.98
**PP15**	2.6243	−0.15805	23	0.98
**PP16**	2.6127	−0.0632	23	0.45
**PP17**	2.65424	−0.07238	23	0.63
**PP18**	2.82495	−0.10615	23	0.86
**PP19**	2.57337	−0.32664	23	0.98
**PP20**	2.37038	−0.2291	23	0.95

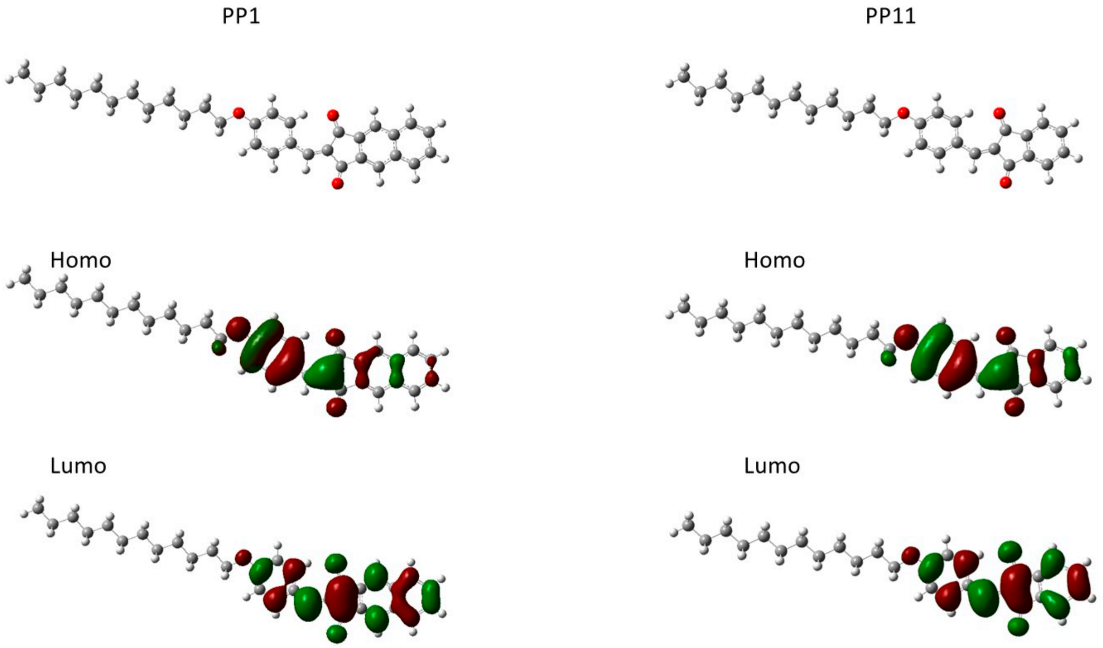

PP2

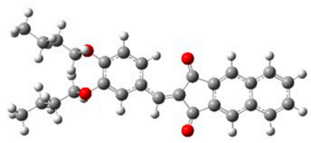

Homo

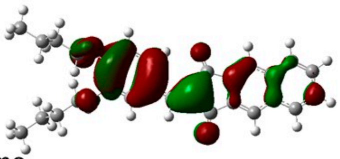

Lumo

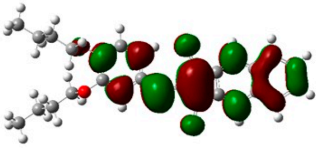

PP12

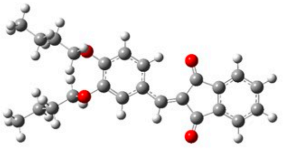

Homo

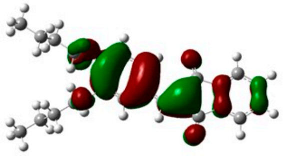

Lumo

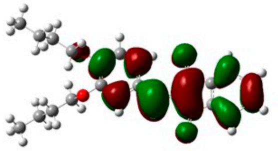

PP3

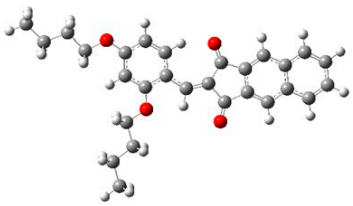

Homo

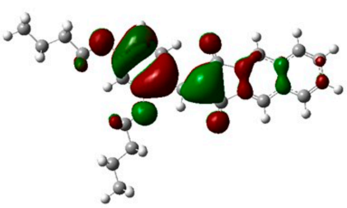

Lumo

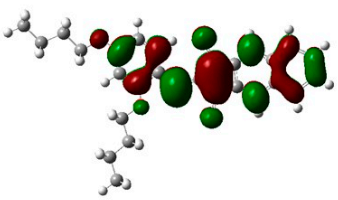

PP13

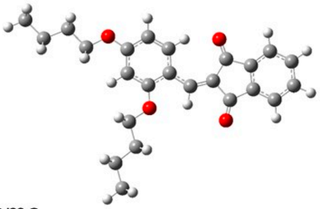

Homo

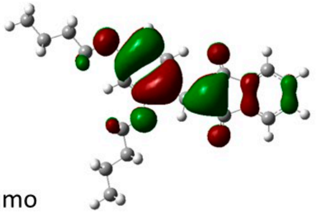

Lumo

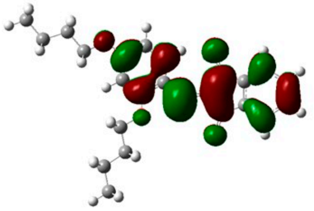

PP4

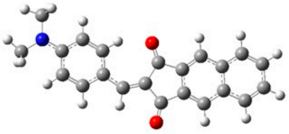

Homo

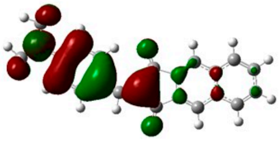

Lumo

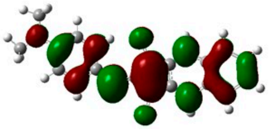

PP14

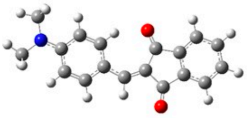

Homo

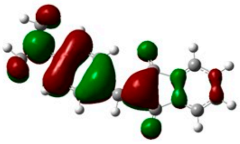

Lumo

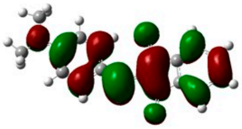

PP5

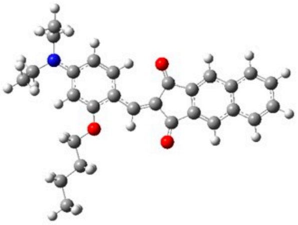

Homo

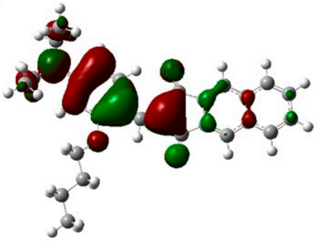

Lumo

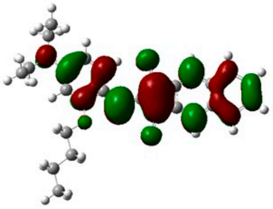

PP15

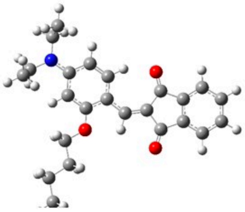

Homo

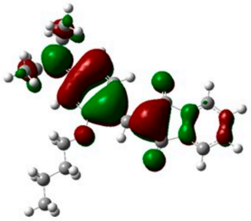

Lumo

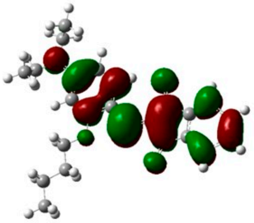

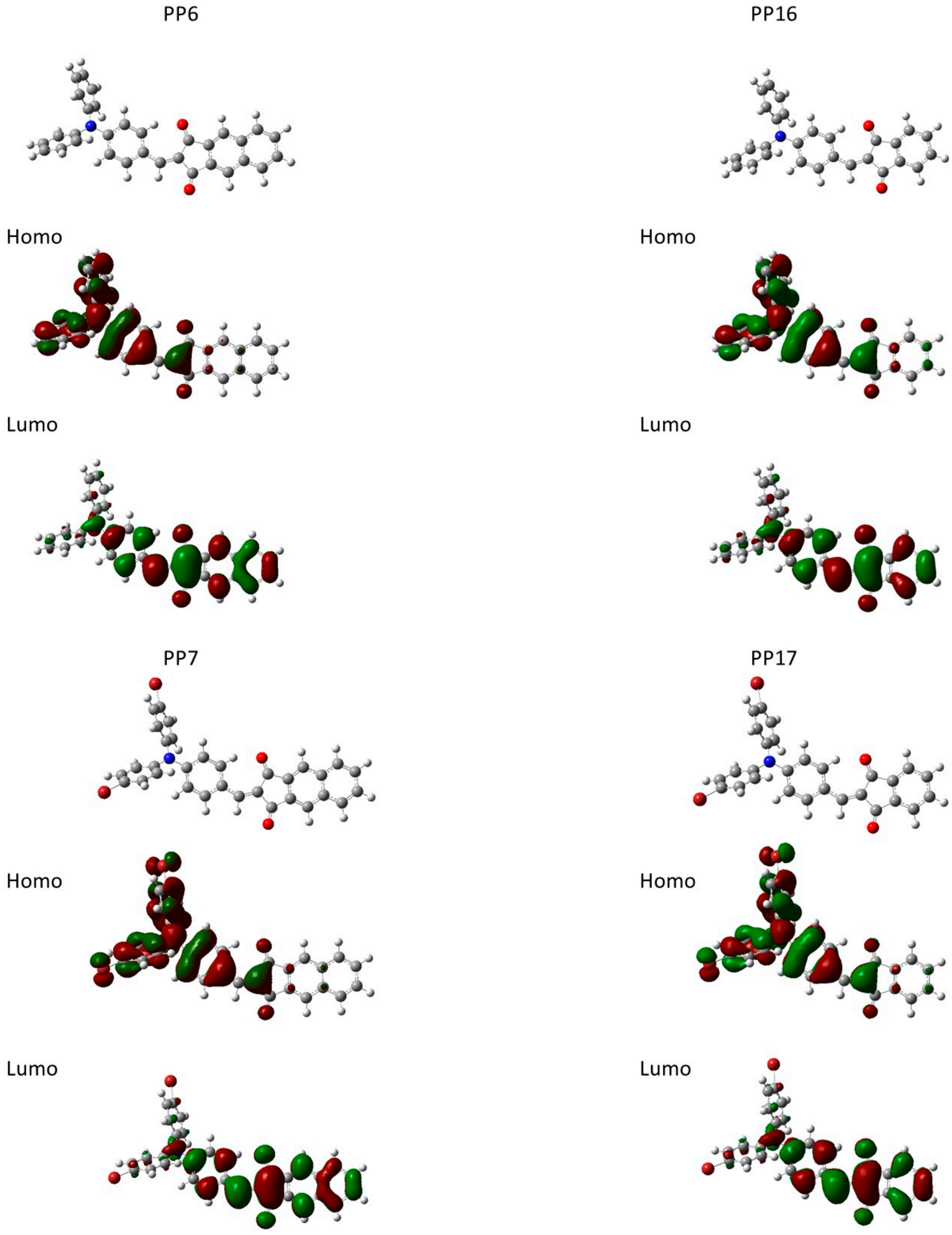

PP6
Homo
Lumo
PP16
Homo
Lumo
PP7
Homo
Lumo
PP17
Homo
Lumo

PP8

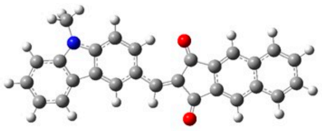

Homo

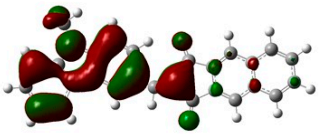

Lumo

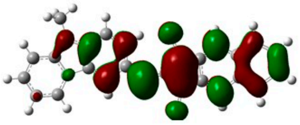

PP18

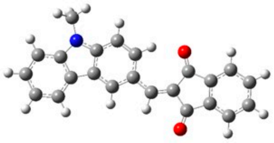

Homo

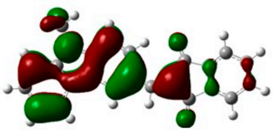

Lumo

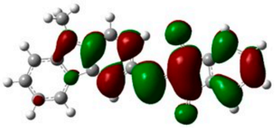

PP9

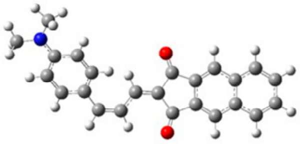

Homo

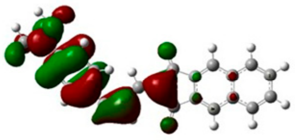

Lumo

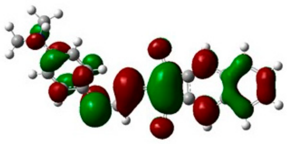

PP19

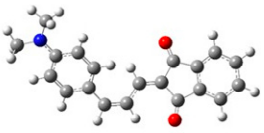

Homo

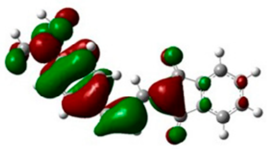

Lumo

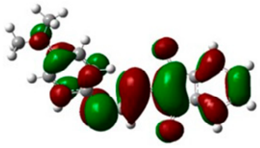

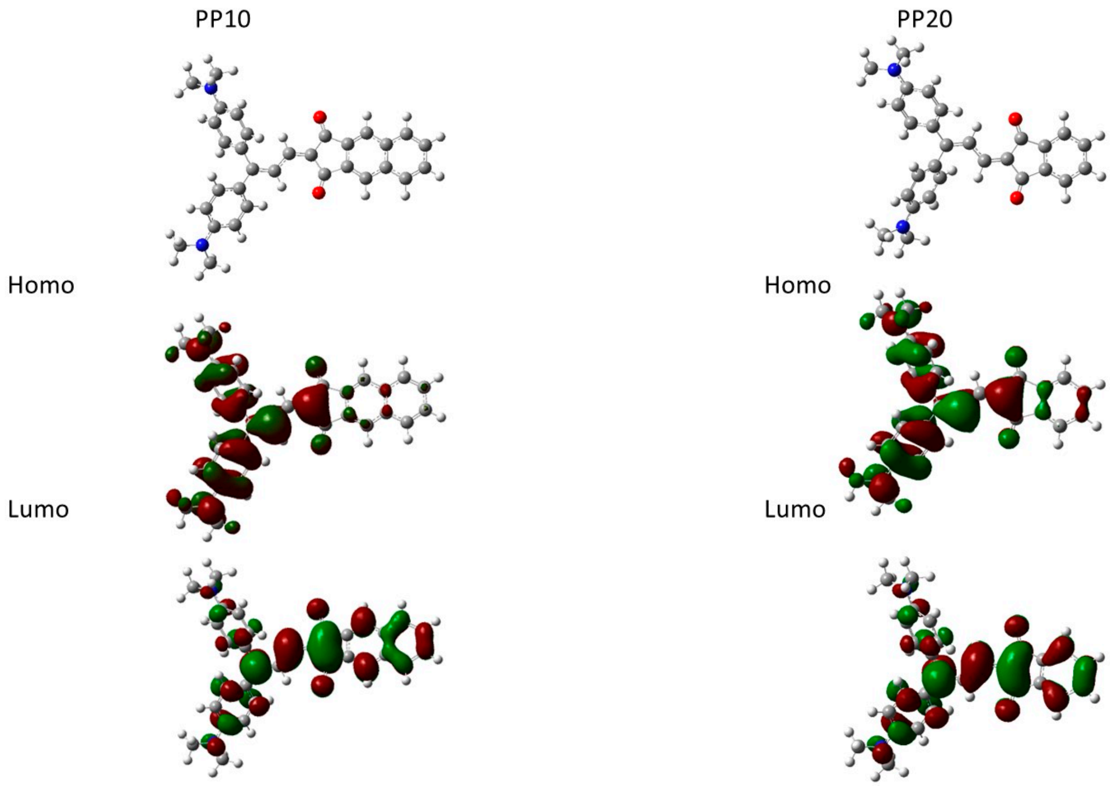

Figure S68. Optimized geometries and HOMO LUMO electronic distribution of all compounds.

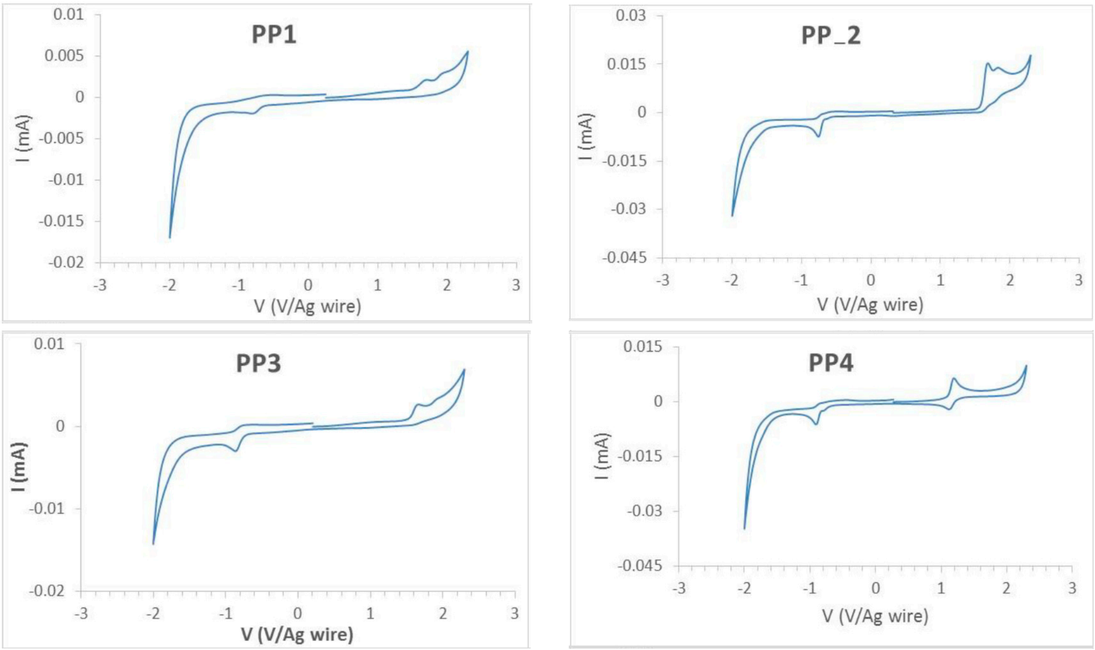

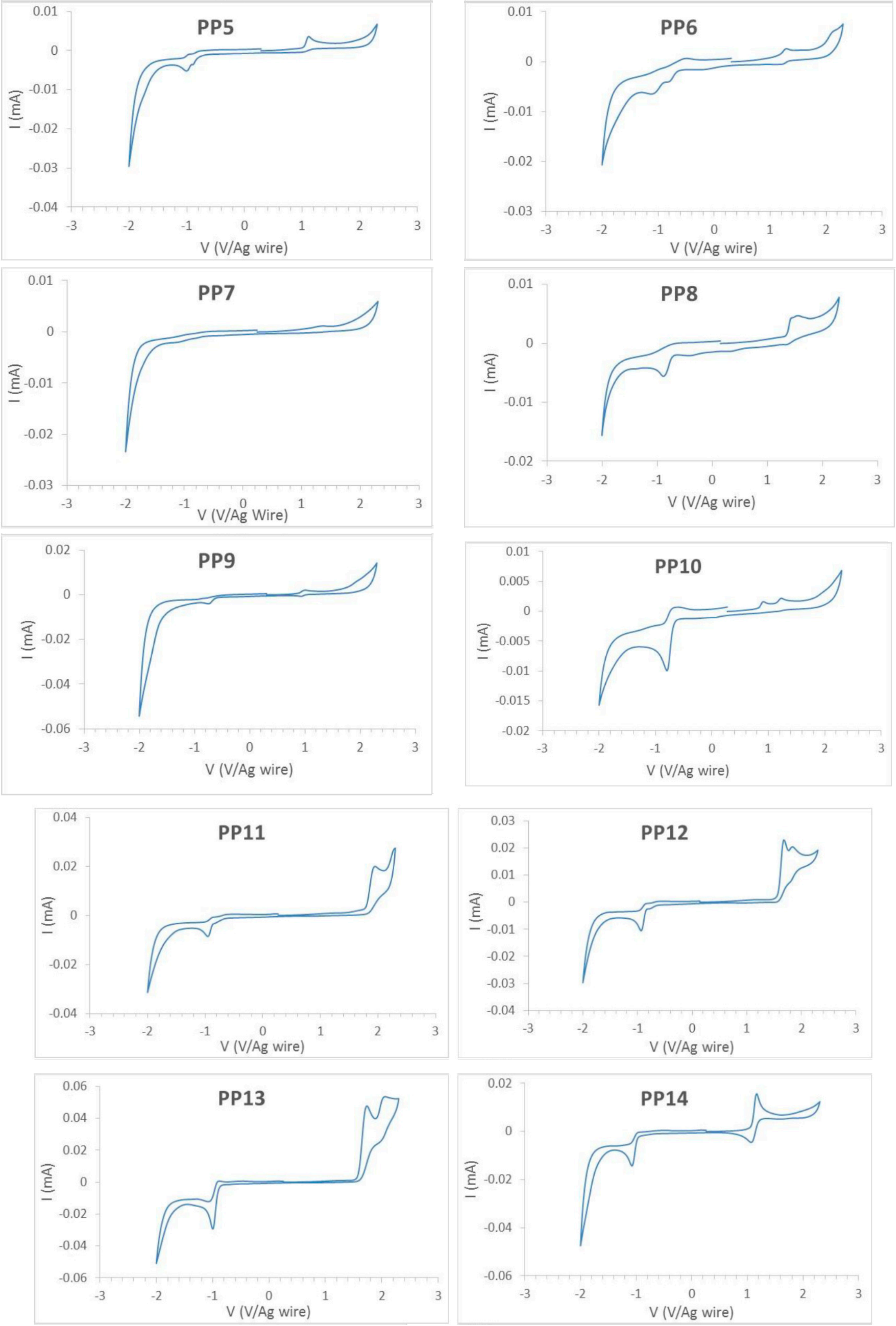
PP5
I (mA)
V (V/Ag wire)
PP6
I (mA)
V (V/Ag wire)
PP7
I (mA)
V (V/Ag Wire)
PP8
I (mA)
V (V/Ag wire)
PP9
I (mA)
V (V/Ag wire)
PP10
I (mA)
V (V/Ag wire)
PP11
I (mA)
V (V/Ag wire)
PP12
I (mA)
V (V/Ag wire)
PP13
I (mA)
V (V/Ag wire)
PP14
I (mA)
V (V/Ag wire)

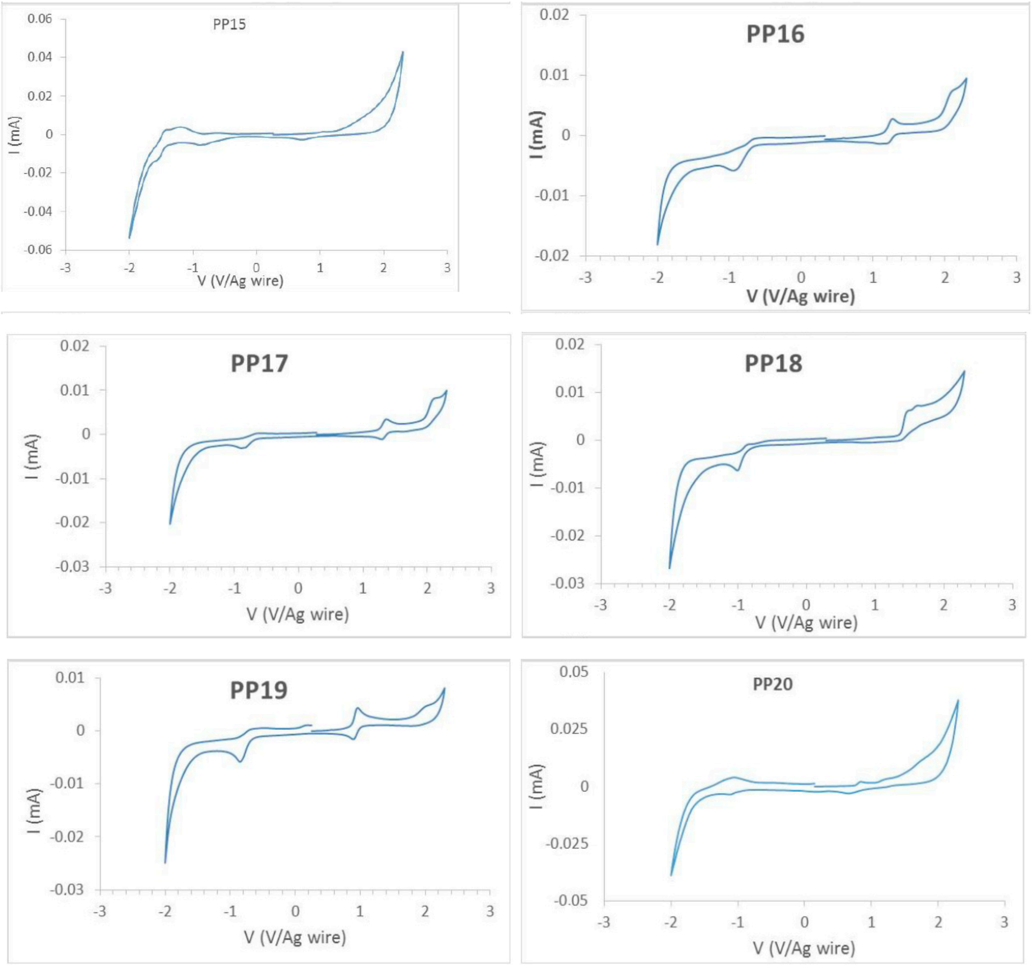

Figure S69. Cyclic voltamograms of push pull compounds (**PP1–PP20**). All cyclic voltamogrammes recorded in 0.1 M $TBABF_4$/ACN, except **PP15** and **PP20** in 0.1 M $TBAClO_4$/CH_2Cl_2.
